# Consensus molecular environment of schizophrenia risk genes in coexpression networks shifting across age and brain regions

**DOI:** 10.1126/sciadv.ade2812

**Published:** 2023-04-14

**Authors:** Giulio Pergola, Madhur Parihar, Leonardo Sportelli, Rahul Bharadwaj, Christopher Borcuk, Eugenia Radulescu, Loredana Bellantuono, Giuseppe Blasi, Qiang Chen, Joel E. Kleinman, Yanhong Wang, Srinidhi Rao Sripathy, Brady J. Maher, Alfonso Monaco, Fabiana Rossi, Joo Heon Shin, Thomas M. Hyde, Alessandro Bertolino, Daniel R. Weinberger

**Affiliations:** ^1^ Lieber Institute for Brain Development, Johns Hopkins Medical Campus, Baltimore, MD, USA.; ^2^ Group of Psychiatric Neuroscience, Department of Translational Biomedicine and Neuroscience, University of Bari Aldo Moro, Bari, Italy.; ^3^ Department of Psychiatry and Behavioral Sciences, Johns Hopkins University School of Medicine, Baltimore, MD, USA.; ^4^ Istituto Nazionale di Fisica Nucleare, Bari, Italy.; ^5^ Azienda Ospedaliero Universitaria Consorziale Policlinico, Bari, Italy.; ^6^ Department of Neuroscience, Johns Hopkins University School of Medicine, Baltimore, MD, USA.; ^7^ Dipartimento Interateneo di Fisica, Università degli Studi di Bari Aldo Moro, Bari, Italy.; ^8^ Department of Neurology, Johns Hopkins University School of Medicine, Baltimore, MD, USA.; ^9^ Department of Genetic Medicine, Johns Hopkins University School of Medicine, Baltimore, MD, USA.

## Abstract

Schizophrenia is a neurodevelopmental brain disorder whose genetic risk is associated with shifting clinical phenomena across the life span. We investigated the convergence of putative schizophrenia risk genes in brain coexpression networks in postmortem human prefrontal cortex (DLPFC), hippocampus, caudate nucleus, and dentate gyrus granule cells, parsed by specific age periods (total *N* = 833). The results support an early prefrontal involvement in the biology underlying schizophrenia and reveal a dynamic interplay of regions in which age parsing explains more variance in schizophrenia risk compared to lumping all age periods together. Across multiple data sources and publications, we identify 28 genes that are the most consistently found partners in modules enriched for schizophrenia risk genes in DLPFC; twenty-three are previously unidentified associations with schizophrenia. In iPSC-derived neurons, the relationship of these genes with schizophrenia risk genes is maintained. The genetic architecture of schizophrenia is embedded in shifting coexpression patterns across brain regions and time, potentially underwriting its shifting clinical presentation.

## Teaser

Putative risk genes for schizophrenia are expressed at early stages and their interactome changes during development.

## INTRODUCTION

Schizophrenia (SCZ) risk increases as a function of genetic relatedness with an affected individual (*1*). The latest rendering of the genome-wide association study (GWAS) approach has identified 287 distinct SCZ-associated loci harboring many more potential risk genes (*2*). Therefore, an important current challenge is to explain how hundreds or perhaps thousands of spatially distant and biologically diverse genes confer risk for SCZ and which biological pathways they affect. Another outstanding question concerns the molecular environment in which genes mapped to GWAS loci exert their function and which other genes play key roles in such a molecular environment. Most studies so far have emphasized the functional characterization of genes mapped to GWAS loci, which likely act upon a molecular environment including genes not GWAS significant. This perspective is particularly intriguing for a disease such as SCZ that has been modeled as a condition with omnigenic heritability (*3*).

As the clinical illness presumably is the emergent manifestation of perturbations in cellular systems biology, not in individual genes of small effect, the study of gene coexpression in the brain is a compelling strategy to identify convergence of SCZ risk in biologically meaningful pathways. Many SCZ risk variants control gene expression (*4*–*9*); thus, gene coexpression may be one mechanism governing the convergence of genes mediating SCZ risk into biological pathways of risk (*10*–*12*). Interindividual genetic variation associated with risk-enriched coexpressed gene sets has also been linked with intermediate phenotypes and clinical traits of SCZ (*13*–*15*).

Although coexpression in the human brain has typically been characterized as a “snapshot” of brains lumped together for a pooled analysis, it stands to reason that brain tissue will present shifting/dynamic coexpression patterns across development and age. A neurodevelopmental perspective of SCZ posits that early developmental dysfunctions in brain regions such as the dorsolateral prefrontal cortex (DLPFC) and the hippocampus may interact with molecular aspects of neurotypical brain development, causing the diagnostic symptoms to emerge later in life (*16*). Brain circuits whose function involves specific aspects of later maturation, such as the surge in dopaminergic innervation of the striatum and cortex in young adulthood (*17*), may contribute to this later onset of clinical symptoms. However, premorbid characteristics of SCZ are well described, including delays in developmental milestones, language, and cognitive capacity (*18*). A provocative yet biologically plausible idea ingrained in the neurodevelopmental perspective is that the same genes, e.g., genes mapped to GWAS loci, may have different effects depending on the molecular environment in which they act at different stages of development.

Notably, the study of gene coexpression related to SCZ risk has focused primarily on postnatal, mostly adult cohorts, thus largely missing a potential neurodevelopmental perspective in which genes mapped to GWAS loci act in concert with partner genes shifting in the course of development. Published reports have considered principally the DLPFC rather than a circuit of brain regions also involved in SCZ risk, including the hippocampus (*19*, *20*), and the associative striatum (*21*, *22*). Although risk convergence is likely more dense in specific cell types (*11*, *23*, *24*), most work has used bulk homogenate tissue data to date. The availability of cell-specific techniques and published datasets now enables a more granular understanding of genetic risk converging into coexpression gene sets within cells, brain circuits, and multiple time points in the life span.

Prior works on autism and SCZ risk have suggested the utility of parsing coexpression into age period–defined cohorts (*25*, *26*). For example, Werling *et al.* (*27*) and others reported remarkable differences between prenatal and postnatal gene expression and expression quantitative trait locus (eQTL) patterns (*28*–*30*). In this and other studies (*31*), however, once coexpression modules had been identified, the set of genes composing a module remained fixed. This procedure is ideal for tracking gene expression trajectories but misses how risk gene sets shift over time, potentially converging and diverging at different ages.

Here, we hypothesized that genes mapped to SCZ GWAS loci (*2*) would show shifting patterns of convergence into gene coexpression pathways at specific time points from early neurodevelopment through aging and that this variation would also have brain region selectivity. We thus defined SCZ gene–enriched modules (named “SCZ risk modules”) based on a stringent consensus between gene lists. We obtained these gene lists by windowing gene proximity to GWAS-significant single-nucleotide polymorphisms (SNPs) detected at *P* = 5 × 10^−8^ (“SCZ risk genes”), as detailed in Materials and Methods and by Pergola *et al.* (*14*). We hypothesized that the placement of a gene within a coexpression gene set is a component of its genetic association with SCZ and that network statistics are also associated with GWAS statistics of a given gene. We tested this hypothesis across ages in a critical circuit implicated in SCZ (DLPFC, hippocampus, and caudate nucleus). By parsing data into age period–specific coexpression networks, we searched for enrichment of coexpression gene sets for SCZ risk, allowing sets to change across age periods. We replicated our main findings using PsychENCODE and other datasets (*32*). Last, we identified a previously unreported set of consistent molecular partners of SCZ risk in these networks and tested whether coexpression relationships of GWAS genes representing the potential molecular environment surrounding SCZ risk genes hold in induced pluripotent stem cells (iPSCs), providing an in vitro platform for mechanistic investigations (*33*).

## RESULTS

Our main dataset consisted of 562 postmortem brains from neurotypical control (NC) European and African American individuals and 186 brains from individuals with a diagnosis of SCZ (Materials and Methods and table S1). We replicated our early developmental main results in DLPFC using three postmortem datasets, including, NC RNA sequencing (RNA-seq) data from (i) 16 individuals aged 6 to 25 years from the UCLA-ASD dataset (*32*), (ii) 21 European and African American ancestry individuals from prenatal age to 25 years old from the BrainSpan collection (www.brainspan.org/static/download.html), and (iii) 48 European and African Americans subjects ranging from prenatal to 25 years old from the Lieber Institute for Brain Development (LIBD) polyA RNA-seq data (*12*, *14*).

Data from the main and replication datasets were preprocessed separately (see Materials and Methods). Demographic information of all individuals (cases and controls) used across studies are shown in table S1. After preprocessing, we used weighted gene coexpression network analysis (WGCNA) to identify coexpressed gene sets (*34*). We included all individuals complying with inclusion criteria (Materials and Methods), regardless of age, for each brain region examined in the benchmark networks that we identified. Following WGCNA nomenclature, throughout this paper, we define as “network” the set of all genes considered, which are clustered into “modules,” i.e., a partition of this graph composed of genes highly connected with each other and not overlapping with any other module. While modules are given arbitrary color names, a particular gene set that includes all genes that remain isolated, i.e., not in any highly connected module, is called the “gray” module.

**Fig. 1.**
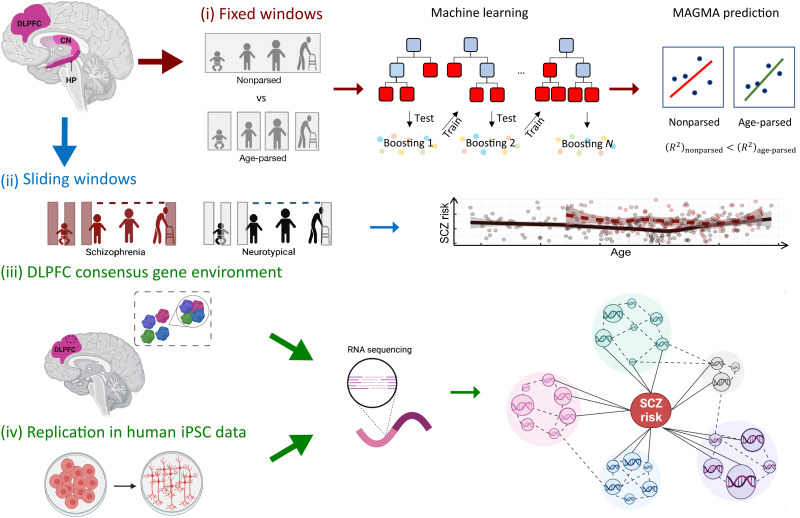
Graphic summary of the study design. In this study, we evaluate four hypotheses: (i) Age-parsed networks in neurotypical brains explain more SCZ genetic risk than the same data not age-parsed. (ii) The course of SCZ enrichment differs between NCs and patients with SCZ. (iii) There is a molecular environment to SCZ risk that can be identified by consensus between networks in terms of genes coexpressed with SCZ risk genes. (iv) The consensus genetic environment surrounding SCZ risk genes can be reproduced in vitro to perform cell system studies of coexpression networks relevant to SCZ. CN, caudate nucleus; HP, hippocampus; MAGMA, multimarker analysis of genomic annotation; SCZ, schizophrenia. The figure was created with BioRender.com and Inkscape.

First, we performed a sensitivity analysis to establish the parameters used to identify SCZ risk modules based on enrichment for SCZ risk genes. To this aim, we used previously published networks (*5*, *10*–*14*, *26*, *27*, *31*, *35*, *36*). We then tested four hypotheses (each outlined in Fig. 1): (i) Age-parsed networks in neurotypical brains explain more SCZ genetic risk than the same data not age-parsed (benchmark networks). To test age parsing effects, we considered ordinal age periods (perinatal, juvenile, adult, and older adult) on the bulk tissue region samples and identified the shifting patterns of module enrichment across the human life span in those age periods (“Age study”). (ii) The course of SCZ enrichment across age differs between NC and patients with SCZ. Here, we used a sliding window approach to age (“Sliding windows”), which, unlike the age-period study, did not model fixed periods but rather the gradual variation of convergence of SCZ risk genes with age across different brain regions and compared coexpression patterns between the NC and the SCZ samples. (iii) There is a molecular environment to SCZ risk that can be identified by consensus between networks in terms of genes coexpressed with SCZ risk genes. We computed the overlap between our networks’ DLPFC SCZ risk–associated modules and previously published networks to obtain a consensus gene set coexpressed with SCZ risk genes (“Consensus genetic environment”). We thus assessed the reproducibility of coexpression gene sets and identified genes that are typically coexpressed with SCZ risk genes, with the assumption that such genes are important for the biology of SCZ risk in the brain. (iv) We tested whether an in vitro biological system reproduces the network features and consensus relationship that we identified in brain in a model of neuronal cells differentiated from human iPSCs (“iPSC study”).

### Parameter setting and regional coexpression

**Fig. 2.**
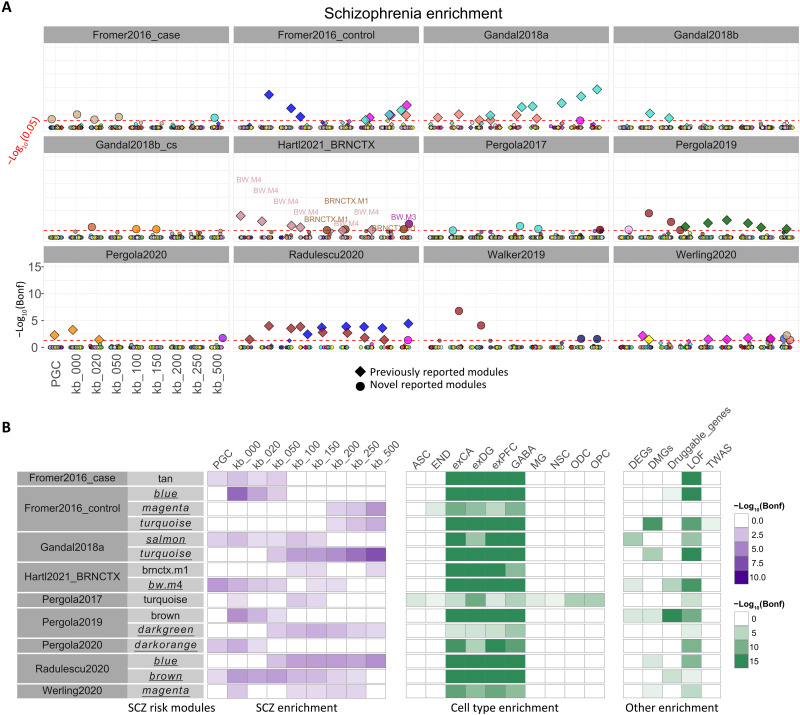
Coexpression modules enriched for SCZ risk. (**A**) SCZ risk gene enrichment in previously published networks. SCZ risk was assessed in the coexpression gene sets in 12 previously published networks for nine reference lists of expanding SCZ risk protein-coding genes [bins: PGC (120 prioritized genes) (*2*), kb_000, kb_020, kb_050, kb_100, kb_200, kb_250, and kb_500]. The *y* axis represents enrichment value −log_10_(Bonferroni-corrected *P* value) for a module in that bin (hypergeometric test; see Materials and Methods). The dotted red line denotes −log_10_(0.05) threshold. Significant modules (Bonferroni-corrected *P* value < 0.05) are magnified. Colors correspond to the name assigned by WGCNA to the coexpression module except in the Hartl2021_BRNCTX network. The gene sets and reference lists were restricted to protein-coding genes. Diamonds (♦) represent modules previously highlighted for SCZ risk enrichment in the respective publications. (**B**) Risk module functional characterization. We identified 15 SCZ risk modules across nine published networks as significantly enriched for SCZ risk in at least three of the nine lists of protein-coding SCZ risk genes. Risk modules were characterized for different enrichments (SCZ, cell type, and other; see Materials and Methods). Only significant enrichments (Bonferroni-corrected *P* value < 0.05) are shown. Module names with underline represent previously reported modules. ASC, astrocytes; DEGS, SCZ differentially expressed genes between patients and controls derived from separate caudate, DLPFC, and HP studies; DMGS, genes proximal to differentially methylated CpG islands in SCZ; Druggable_genes, SCZ drug target genes; END, endothelial cells; exCA, pyramidal neurons from the hippocampal CA region; exDG, granule neurons from the hippocampal dentate gyrus; exPFC, pyramidal neurons from the prefrontal cortex; GABA, GABAergic interneurons; LOF, loss-of-function intolerant genes; MG, microglia; NSC, neuronal stem cells; ODC, oligodendrocytes; OPC, oligodendrocyte precursor cells; TWAS, SCZ transcriptome-wide association study genes derived from separate caudate, DLPFC, and HP studies.

We aimed to identify modules enriched for genes in the proximity of genome-wide significant SNPs, hence limiting our search to loci whose association with SCZ was corrected for multiple comparisons. As the loci defined by Trubetskoy *et al.* (*2*) comprised 2194 genes, representing a sizable fraction of the transcriptome, we evaluated several sets of genes at multiple genomic distances from the tagging SNP (*35*), plus the set of 120 genes prioritized by the authors of the third wave of the Psychiatric Genetics Consortium (PGC3) manuscript (see Materials and Methods). We hypothesized that the distance of coexpressed genes from SNPs varied across different modules, and these data could be informative about the particular SNPs and genes involved. We leveraged data from published networks to establish an appropriate tradeoff between sensitivity and specificity. Table S2 shows the Bonferroni-corrected significant module count for each network assessed for enrichment for protein-coding genes in the proximity of SCZ-significant SNPs. There was remarkable variability in terms of distance of the significant gene set from the SCZ-significant SNPs (Fig. 2A), as indexed by a coefficient of variation of 1.14. The coefficient of variation was computed as SD/mean of the peak enrichment window genomic distance from the index SNP. The value above 1 indicates that the SD was larger than the mean [SD = 214 kilo–base pair (kbp); mean = 186 kbp]. We prioritized modules enriched in at least three genomic distance windows (note S1.1). Figure 2B shows the characterization of these modules in published networks from the DLPFC. By and large, we rediscovered modules already prioritized in the original reports, but the number of modules that we identified with this criterion was generally lower than the original reports, hence reflecting greater stringency. We also identified some modules not previously reported in these articles preceding the PGC3 publication (*2*). The biological characterization of these modules reflected neuronal biology and enrichment for loss-of-function intolerant genes (Fig. 2B). Following this calibration step, we used the same criterion and prioritized modules enriched in three genomic distance windows in our networks.

### Study 1: SCZ risk gene enrichment across brain regions and age

#### Age parsing improves gene importance prediction models

Most previous studies along with the bulk tissue networks that we identified here across all ages embedded age information in network identification. Here, we generated age period–specific networks. Age periods were defined as a trade-off between sample size and biological meaningfulness: (i) perinatal, fetal life to 6 years; (ii) juvenile, 6 to 25 years (birth to 25 years for caudate nucleus); (iii) adult, 25 to 50 years; and (iv) older adult, over 50 years.

We obtained 13 age period–parsed gene coexpression networks (four age periods × three brain regions without perinatal caudate and two replication networks). Table S3 summarizes the descriptors of these networks. Given the evidence implicating early neurodevelopmental components of SCZ (*26*, *37*), our central hypothesis in the age study was that age-parsed coexpression explained SCZ genetic risk better than nonparsed coexpression. To assess the explanatory power of parsed networks, we investigated the genetic convergence of the SCZ genetic signal by a continuous measure of that signal across all genes. To this end, we used MAGMA (multimarker analysis of genomic annotation) in its revised version (*38*, *39*) to compute for each gene a *Z* score of association with SCZ based on GWAS effect size (*2*) of SNPs in the GWAS loci. First, we assessed whether splitting the datasets into age periods with a smaller sample size enhanced the accessible biological information. We computed linear models to associate gene importance with coexpression, in which the outcome variables were MAGMA scores obtained for each gene and predictors were module assignment (categorical) and total connectivity of each gene in each network. We compared linear models obtained with the entire cohort (no parsing in three brain regions: DLPFC, hippocampus, and caudate nucleus) versus the 11 age period–parsed networks.

We first evaluated the direct relationship between total gene connectivity and MAGMA score (an example scatter plot can be seen in fig. S1). We found a significant positive correlation in all networks except the perinatal (all other Bonferroni-corrected *P* < 0.001). This result suggests that genes with higher SCZ MAGMA scores are generally more strongly connected with all other genes, although the effect size was small for all (all Kendall’s tau < 0.054).

**Fig. 3.**
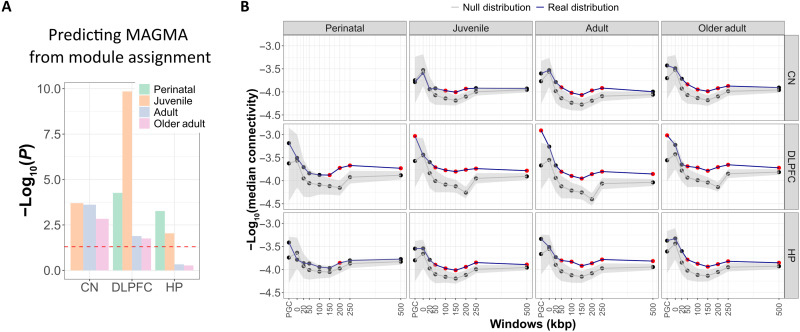
SCZ risk is better explained in age-parsed networks. (**A**) A linear model was used to predict MAGMA score from module assignment and total connectivity predictors considering all 11 age-parsed networks. The graph shows the −log_10_(*P*) for each age-parsed network to predict MAGMA scores from module assignment within this linear model. (**B**) Within-set gene connectivity (coexpression) for different SCZ gene sets, across tissue and age. On the *y* axis is the median of the gene-wise connectivity for each set, with each SCZ gene set represented on the *x* axis. Connectivity values were compared to a null distribution of gene sets of equal size, length, GC content and average expression. The null 95% confidence interval (CI) is in gray shade; the mean value is the gray line. Red dots represent SCZ gene sets above the null 95% CI.

Both nonparsed and age-parsed gene module assignments were significantly associated with MAGMA scores (complete statistics in note S1.3). In age-parsed networks, we found that perinatal and juvenile networks consistently, across all tissues, showed a stronger association between SCZ importance and module assignment than adult networks (Fig. 3A). This finding suggests that genetic risk for SCZ is more aggregated in networks during earlier development than in adulthood, hence pointing to a functional role of SCZ risk genes in early development. Thus, evaluating gene assignments and trajectories during these early stages is relevant for understanding the biology of SCZ risk. In addition, perinatal hippocampus total gene connectivity was negatively associated with MAGMA, which was not the case in DLPFC networks. This result means that genes most associated with SCZ were overall less connected with all other genes (and thus less central in the network) in hippocampal in contrast to DLPFC networks, independently of their module assignment.

**Fig. 4.**
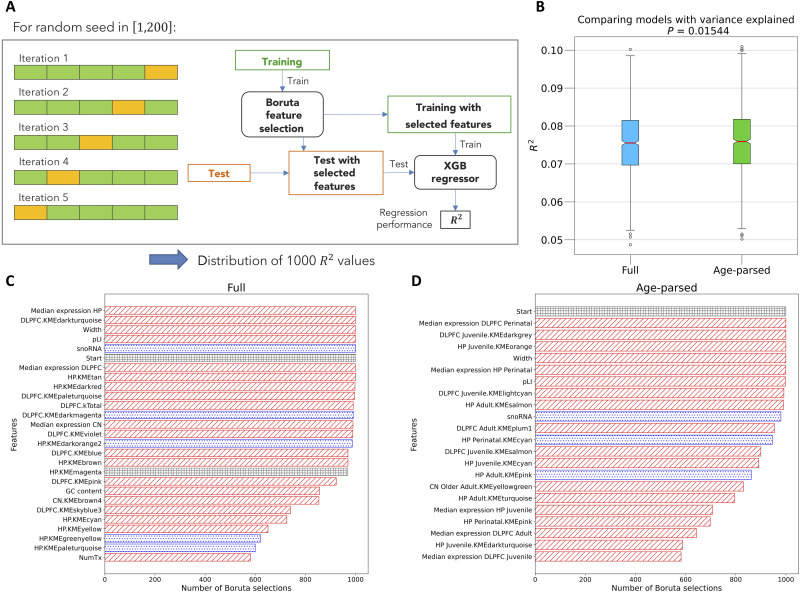
The proposed machine learning model is more effective at predicting MAGMA score when trained on age-parsed rather than age-aggregated network features. (**A**) Workflow of the analysis: Using a random number generator, the 21,751 genes are randomly assigned to five different partitions, and for each partition, the algorithm performs a round of a fivefold cross-validation, stratified with respect to the chromosomes to which genes belong; then, it applies the Boruta feature selection algorithm to the training set and trains an XGBoost regressor on the same set, using only the selected features. XGBoost predicts the MAGMA score of genes in the test set; One thousand values of *R*^2^ are collected during the analysis, corresponding to different random seeds and different iterations of fivefold cross validation. (**B**) Distributions of the 1000 *R*^2^ values obtained in the MAGMA predictions based on the Full dataset (light blue), containing variables related to expression and connectivity in the three age-aggregated networks, and on the Age-parsed dataset (green), containing analogous variables for the 11 age-parsed networks. The Wilcoxon test reveals a statistically significant (*P* = 0.01544) improvement of performance in the Age-parsed dataset with respect to the Full dataset. (**C**) Features of the Full dataset selected by the Boruta algorithm in more than 500 runs. The bars are filled with red lines, blue dots, or gray squares, depending on whether the Pearson correlation of the corresponding feature with the MAGMA score is, respectively, positive and statistically significant (*P* < 0.05), negative and statistically significant (*P* < 0.05), or not significant. (**D**) Features of the Age-parsed dataset selected by the Boruta algorithm in more than 500 runs. The meaning of the patterns used to fill the bars is the same as for (C).

Computing age-parsed networks explained significantly more variance in MAGMA scores for SCZ than using the same samples without age parsing to compute a single network per tissue (Vuong test for non-nested models, *z* = 8.4, *P* < = 2.2 × 10^−16^). To validate this result, we implemented a regression algorithm to predict MAGMA scores to compare the prediction performance of our machine learning workflow (Fig. 4A; see Materials and Methods and method S2.2) between age-parsed and nonparsed datasets (see Fig. 4). Both datasets contained 21,751 genes and the same intrinsic attributes but different expression and coexpression properties.

The *R^2^* distributions obtained for the Full and Age-parsed datasets are shown in Fig. 4B. A Wilcoxon test indicated a significant improvement (*P* = 0.015) in the performance obtained with the Age-parsed dataset. Among the most relevant features in predicting the MAGMA score were intrinsic attributes such as gene width and GC content, the median expression variables, and the module membership metrics (KME) associated with the modules of the coexpression network (Fig. 4, C and D). Explanatory KME variables for modules that are not significantly enriched for SCZ are related to predicting the entire range of MAGMA score values, also including low-score genes (some module membership variables have a significant negative correlation with the MAGMA score). In the age-parsed prediction models, the features selected in each and every run included the median gene expression in perinatal DLPFC/hippocampus and juvenile DLPFC/hippocampus module membership but not in adult age stages.

Together, these analyses suggest that (i) genes with high MAGMA score for SCZ tend to be coexpressed, as module membership contributes to SCZ importance prediction into an unseen testing set, and (ii) parsing genes into age periods, data being equal, reveals stronger associations between networks and a continuous measure of gene association with SCZ beyond GWAS-significant loci.

#### SCZ top loci genes converge into coexpression modules in multiple brain regions, though especially in early-stage DLPFC

The publications we used to perform the sensitivity analysis for module characterization focused on cortical samples. We then compared these published results with our networks from DLPFC, hippocampus, and caudate nucleus with and without parsing for age. We interrogated the properties of SCZ risk genes across age-parsed networks, asking whether sets of putative SCZ risk genes (defined from PGC3 and at varying genomic windows from PGC3 SNPs) were highly coexpressed with each other regardless of module membership. We found that SCZ risk genes are more coexpressed with each other as compared to a null distribution of gene sets of equal set size, gene length, GC content, and average expression. Results vary across brain regions and age, with peak statistics around 200 kbp from GWAS-significant SNPs (Fig. 3B). The perinatal results were not principally driven by postnatal samples, as shown by significant departure of connectivity estimates from the null distribution (windows: 150 to 500 kbp; *P*[empirical] < 0.001).

**Fig. 5.**
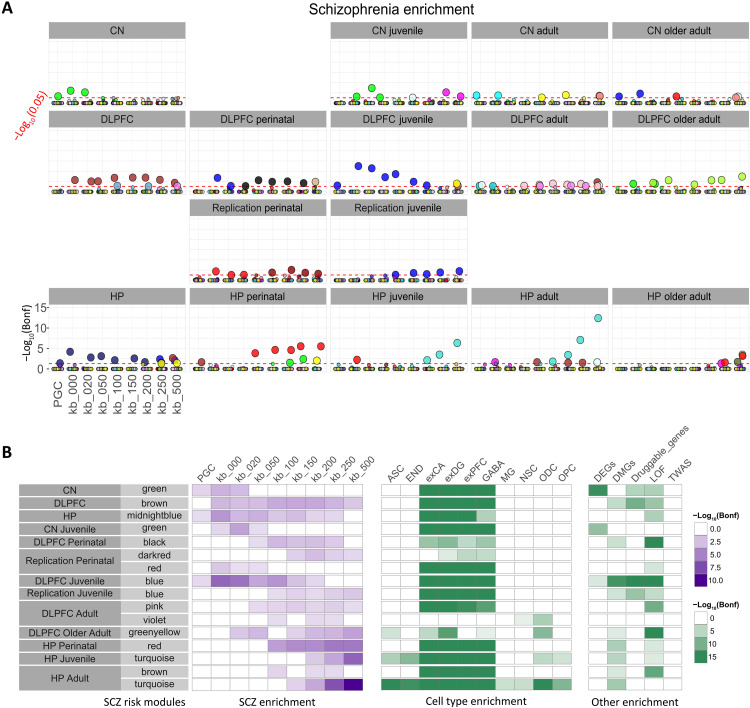
SCZ risk modules in networks parsed by age. (**A**) SCZ risk genes enrichment in the generated networks. Only networks with modules significantly enriched for SCZ top protein-coding risk genes are shown in (**B**). See Fig. 2 caption for details on the tests computed and abbreviations in (A) and (B).

In most networks, SCZ risk genes were depleted, i.e., underrepresented in hypergeometric tests, in the gray module, more extensively than control gene sets negative for SCZ. Because gray modules in WGCNA are composed of nonclustered genes, these results suggest that SCZ risk genes tend to be coexpressed in modules with other genes, rather than not (note S1.4 and fig. S2). This finding is consistent with the correlation between MAGMA scores and connectivity shown above. The underrepresentation in gray, however, may relate at least in part to the fact that SCZ risk genes tend to be more expressed in brain tissue and highly expressed genes are less likely to be in the gray module (note S1.1 and fig. S3). Eight of 11 age-parsed networks included at least one SCZ risk-enriched module (Fig. 5A). The genomic window in which most of these modules were significant included genes localized up to 200 kbp from the SCZ index SNP. SCZ risk modules were generally selective for SCZ genes and were not enriched for other disorders (fig. S4), although hippocampal modules were an exception, representing enrichment for multiple pathologies. DLPFC enrichments were not driven by many genes localized in few loci but were characterized by the convergence of many loci, while the opposite case was seen in the hippocampus (note S1.4). Considering that all DLPFC networks identified coexpression convergence in at least one module, DLPFC aggregation of SCZ risk genes appears to endure across multiple age periods in contrast to caudate or hippocampus, which are relatively age restricted. In summary, the coexpression of genes in the proximity of GWAS-significant SNPs associated with SCZ is found most convincingly in the DLPFC.

SCZ risk modules had predominantly neuronal cell specificity (Fig. 5B). Accordingly, a study of dentate gyrus tissue enriched for granule cells via laser caption microdissection (*23*) showed a greater degree of SCZ risk gene convergence on coexpression patterns compared to bulk hippocampus RNA-seq and insight into the context-dependent functions of these genes otherwise unavailable (note S1.2, methods S2.1, and fig. S5). In addition, several of the age-parsed modules were enriched in genes proximal to CpG islands differentially methylated in SCZ, which was not the case in nonparsed networks, suggesting a different gene aggregation and consistent with the expected epigenetic variation associated with age. Note how this property contrasts with the sparse other enrichments found in previously published enriched modules, which were not parsed by age and mainly relied on adult brain samples (Fig. 2B).

**Fig. 6.**
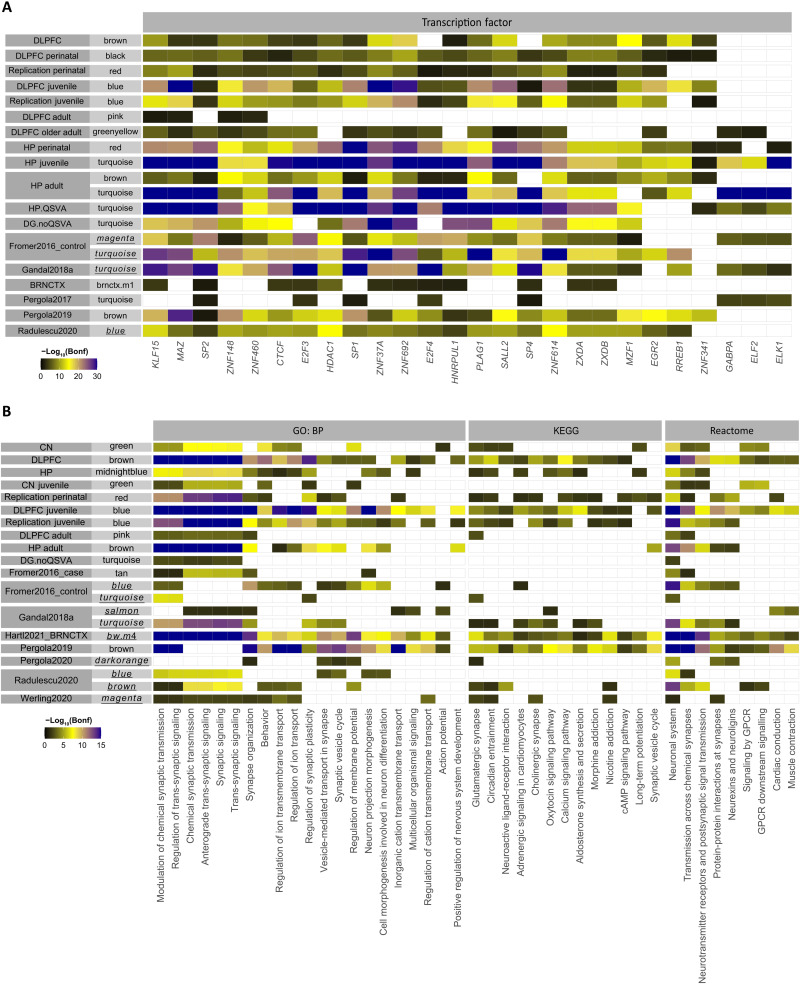
Regulome enrichment and functional characterization of schizophrenia risk modules. To obtain this figure, transcription factor target enrichments were first restricted to corrected *P* < 0.05. Then, for modules significant for SCZ in at least three extension windows (shown in Figs. 2 and 5 and fig. S5), a union of top three most significant transcription factors for each module is considered for plotting. A white block for a module indicates that the targetome of the transcription factor is not significantly overrepresented in that module. (**A**) Transcription factor targetome enrichment. (**B**) Gene ontology: biological processes (GO: BP), Kyoto Encyclopedia of Genes and Genomes (KEGG), and Reactome pathways. DG, dentate gyrus granule cell layer. For both (A) and (B), processes/transcription factors significant in less than four risk modules and risk modules with less than two significant processes/transcription factors are not shown. Underlined module names represent modules previously reported as associated with SCZ.

Regulomic analyses identified several transcription factors whose targets were overrepresented across multiple networks (Fig. 6A), with the most evidence favoring *KLF15*, *MAZ*, and *SP2*. Other transcription factors of the *SP* family with a targetome profile similar to *SP2* were also included, such as *SP4*, a gene prioritized by the PGC3 GWAS (*2*) and enriched for rare damaging coding variants in SCZ at exome-wide significance (*40*). SCZ risk modules were similar across regions in terms of top gene ontologies (e.g., involving synapses; Fig. 6B).

#### Risk convergence trajectories point to specific and shared gene functional profiles in the DLPFC and hippocampus across age

**Fig. 7.**
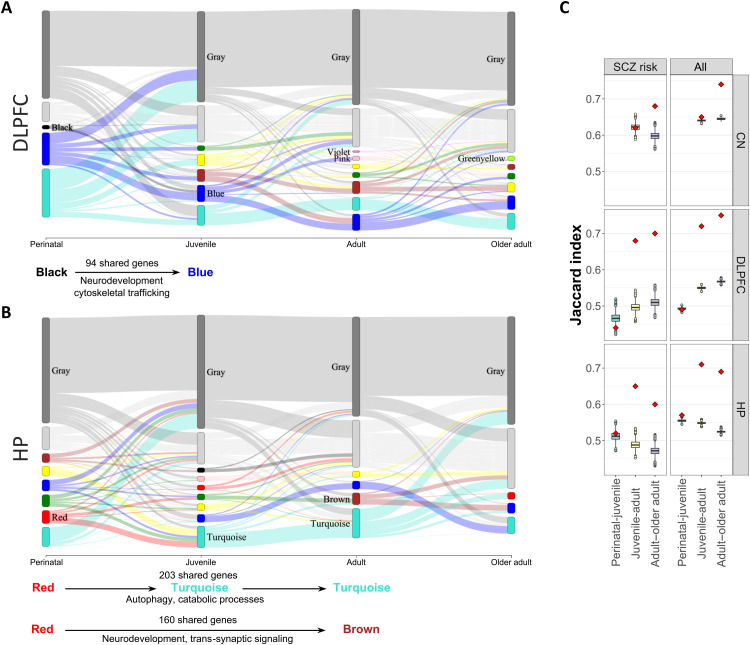
Coexpression transitions of SCZ risk genes at various age periods. Module membership of SCZ risk genes (200-kbp list; see Materials and Methods) for age-parsed networks arranged ordinally. Modules for a network are represented as nodes. The size of node corresponds to the number of SCZ genes in the module. The link width represents the number of shared SCZ genes between modules from two age-parsed networks. Only the risk modules (from Fig. 5) are labelled. The bottom text highlights select conserved “streams” between risk modules across age. (**A**) DLPFC age-parsed networks. (**B**) HP age-parsed networks. In (A) and (B), a general discontinuity from perinatal to postnatal ages is evident, but some risk gene sets continue to be expressed in the perinatal-to-juvenile transition. For clarity of illustration, modules with less than 30 SCZ risk genes for the 200-kbp list that are not enriched for SCZ are combined into a hypothetical “lightgray module.” (**C**) Gray composition age transitions in JI across brain regions, where box plots depict the null distribution, and the red diamond represents the actual JI measured. The left panels considered only SCZ risk genes, whereas the right panels considered all genes. Notice a transition with low JI between the gray modules of the perinatal and juvenile period in DLPFC. The gray module represents the unclustered genes not belonging to any modules in the network.

The premise of this work was that SCZ risk gene coexpression might change across ages. We measured the overlap between modules in sequential age periods in terms of Jaccard index (JI) at 200 kbp (as reported above, the extension window with the greatest number of significant SCZ risk modules). In the DLPFC, most perinatally coexpressed SCZ risk genes (module black) continue to be coexpressed in juvenile samples (module blue), along with many of their coexpression partners (Fig. 7A), a pathway strongly enriched for neurodevelopment (note S1.5 for additional details and statistics). In the context of a prominent overlap between SCZ risk modules across age periods, the coexpression of a cohesive subset of SCZ risk genes remained high (67% still coexpressed and 33% shifted), while most of their module partners shifted (52% partner genes). As the perinatal window included individuals aged from fetal life to 6 years old, we performed a sensitivity analysis to assess whether this difference in continuity of coexpression after birth was driven by postnatal samples (note S1.5). We found that the prenatal module red including only fetal samples recapitulated the perinatal black enriched module (preservation *Z* score = 41; see fig. S6); also in this case, the proportion of SCZ risk genes coexpressed in the juvenile age stage was higher (46% prenatal genes still coexpressed in the juvenile samples and 54% shifted) than the proportion of non-SCZ risk genes (68% partner genes shifted). We further reproduced this finding in our replication datasets, where we identified another perinatal and another juvenile DLPFC network, respectively. In the replication perinatal DLPFC, module red included 21 SCZ risk genes, of which 12 (57%) were also coexpressed in the replication juvenile blue module, whereas 43% shifted module membership. The 66 genes overlapping between perinatal and juvenile SCZ risk modules were strongly enriched for modulation of chemical synaptic transmission (17 genes, *P*_adjust_ = 2.46 × 10^−7^) and regulation of synaptic plasticity (9 genes, *P*_adjust_ = 6.64 × 10^−6^). Once again, while most SCZ risk genes were still coexpressed, their non-SCZ partners shifted (64%). Accordingly, table S4 shows that all SCZ risk genes most consistently coexpressed into SCZ risk modules, even in adult age, were already coexpressed in perinatal age.

In the hippocampus, the SCZ risk module in the perinatal age period split into two separate developmental coexpression pathways: One mirrored early DLPFC age period coexpression related to neurodevelopment, and another involved autophagy functions. In the DLPFC, autophagy was associated with SCZ risk genes only in the perinatal age period, suggesting that the hippocampus continues coexpressing autophagy-related genes with SCZ risk genes in adulthood (Fig. 7B). The pattern of continuity and shift in coexpression that we observed in perinatal and juvenile DLPFC was not apparent in the hippocampus, neither in the main dataset nor in the replication (note S1.5). Caudate age-parsed networks were not considered because we only found an enriched module in the juvenile network (Fig. 5).

Sankey plots (Fig. 5, A and B) further show that gray module composition changes between perinatal to juvenile periods (juvenile to adult in the caudate nucleus, Sankey plot not shown) and later transitions. These changes were quantified by computing a JI across age adjacent gray modules. Across early life transitions, the JI measured between gray modules did not significantly differ from the null distribution (gray modules were no more similar than two randomly selected gene sets), indicating that gray module composition changes between early life periods. Instead, the JI was significantly higher across later life transitions than the null distribution, indicating that gray composition remains stable in later life periods (Fig. 7C). For additional results, along with Sankey plots for all genes, not just SCZ risk genes, in the networks, see note S1.5. Interactive versions of full Sankey plots are available online at https://doi.org/10.5281/zenodo.5676480.

### Study 2: Differences between neurotypical and patient-derived networks

#### Lifespan trajectories of coexpression show early SCZ risk convergence and differences between patients with SCZ and NCs

**Fig. 8.**
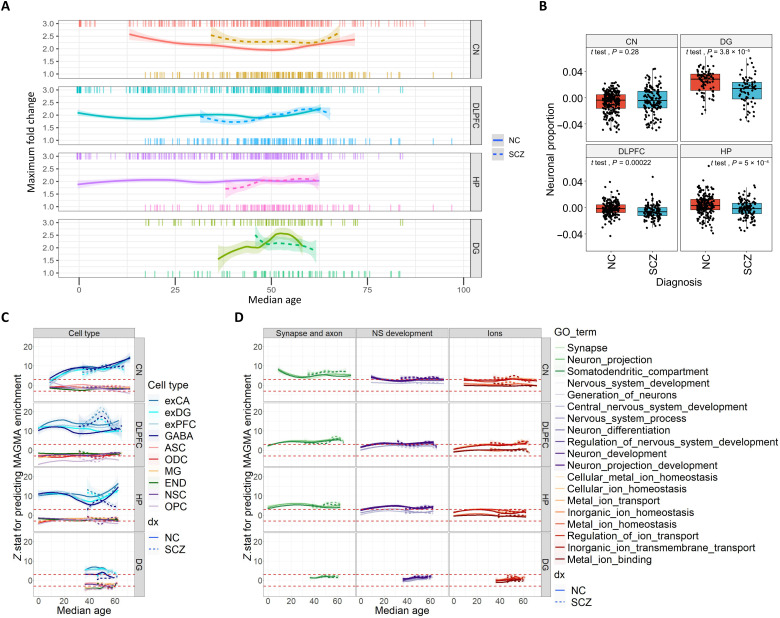
Across module association of cell type and GO with MAGMA enrichment in sliding window networks. (**A**) Overrepresentation of SCZ top loci genes in age-varying sliding windows. Multiple coexpression networks were identified with samples first chronologically arranged and then selected via a sliding window of width 40 samples, shifting one sample at a time. The *x* axis represents the median age of individuals in the window. The *y* axis represents the maximum SCZ fold enrichment (200-kbp list) among all modules in that window. Only the smoothened line connecting maximum fold enrichments is shown. The shaded area represents the 95% CI of smoothened line. The top rug and bottom rug show the individual NC and SCZ sample ages, respectively. (**B**) Neuronal proportion in age-matched samples. Estimated neuronal proportion was significantly greater in neurotypical individuals than patients with SCZ in DLPFC, HP, and DG (*t* test; *P < 0.05). (**C**) We derived *P* values for cell type and MAGMA enrichment for all modules in all sliding window networks. A robust linear model was constructed for each network to evaluate the association between cell type enrichment and MAGMA enrichment. (**D**) The gene ratios of GO term lists were also associated with MAGMA enrichment scores across modules. For (C) and (D), the *y* axis shows the *Z*-stat for cell type or GO term enrichment predicting MAGMA enrichment; the *x* axis shows the median age of the sliding window network. The red dashed lines indicate a nominal *P* = 0.05. SCZ, patients with schizophrenia; NS, nervous system; NSC, neural stem cells.

Given that preassigned age periods do not capture the gradual changes intrinsic to neurodevelopment and results may be affected by the sampling density of subjects along the age dimension, we identified networks in sliding windows of 40 subjects with steps of one subject and compared NC with patients with SCZ. The same samples analyzed in the fixed age period study were used here, with the addition of cohorts of patients with SCZ. We thus generated 316 networks from the DLPFC (221 from NC and 95 from patients with SCZ), 306 networks from the hippocampus (234 from NC and 72 from patients with SCZ), 329 networks from the caudate (219 from NC and 110 from patients with SCZ), and 77 networks from dentate gyrus (46 from NC and 31 from patients with SCZ). Figure 8A shows early SCZ enrichment in neurotypical DLPFC that is superseded later, first by the hippocampus and then by the caudate. The most notable finding is that networks derived from patients with SCZ persistently show greater enrichment for SCZ in caudate nucleus. In contrast, the dentate gyrus data, enriched for neuronal cell type, mainly showed higher enrichment in controls at the age range overlapping with patient-derived networks, reaching a higher peak of enrichment than most other brain regions across age. Although different age trends may reflect age-related changes in cell composition in patients with SCZ or the effect of drugs, stress, chronicity, etc., it is noteworthy that estimated neuronal proportion based on cell decomposition did not explain the difference in the caudate observed between patients and controls (Fig. 8B).

To further characterize these networks biologically, we correlated MAGMA gene scores representing the association with SCZ with gene ontology enrichments across modules within each sliding window network (Fig. 8, C and D). There is a strong association with neuronal cell types in both cases and NC at all ages, except in young caudate NC networks (Fig. 8C). This association suggests a large overlap of neuronal and SCZ enrichment across modules. Gene ontology term associations also show consistency between NC and case networks. The most significant gene ontology terms are those related to synapse and axon, which are above nominal significance across all ages in the caudate, DLPFC, and hippocampus (Fig. 8D, green lines). The only differences across diagnoses are found in the caudate with synapse- and axon-related terms, suggesting a greater overlap in modules between enrichment for SCZ risk and for synaptic function in patients. This result also suggests that the SCZ risk genes responsible for the greater convergence found in networks derived from patients (Fig. 8A) are preferentially synaptic genes. In the hippocampus, modules derived from patients with SCZ are relatively less enriched for GABAergic neurons compared to excitatory neuronal types (Fig. 8C). This brain region also shows the strongest association to nervous system development (Fig. 8D, violet lines), found at the same level of hippocampus synapse and axon terms, which is interesting given the potential role of adult neurogenesis in this region.

### Study 3: Consensus genetic environment

#### Identification of reliable coexpression partners of SCZ risk genes in perinatal and later postnatal prefrontal cortex

Last, we performed a consensus analysis to test the hypothesis that a consistent molecular environment surrounds SCZ risk genes, i.e., a potential locus of molecular convergence involved in how diverse small effect genes might translate toward a common phenotype. Across multiple analyses, SCZ risk genes were most robustly coexpressed in neurotypical DLPFC modules, as outlined above. Considering that most previous studies of gene coexpression networks investigated the DLPFC, we adopted a consensus approach to identify the genes most often included in coexpression modules overrepresenting top SCZ loci genes in the current and these prior studies.

**Fig. 9.**
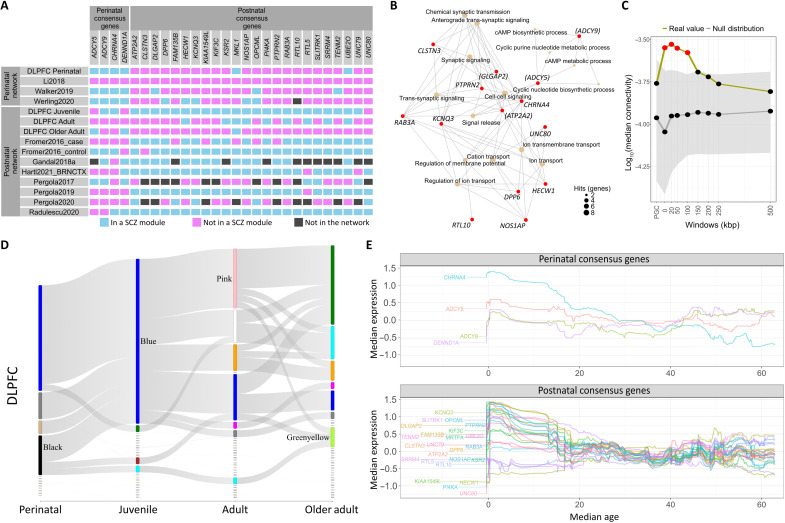
Consensus genes and their relationship with PGC3 prioritized genes. (**A**) Overlap of four perinatal and 24 postnatal consensus genes in the prefrontal networks according to their membership to a SCZ risk module across at least two-thirds of 11 networks. Cyan boxes represent genes in SCZ risk modules. Magenta boxes represent membership to modules not significantly enriched for SCZ. Dark gray boxes represent genes not annotated in that specific network. The figure illustrates how coexpression partners of SCZ risk genes change from perinatal to postnatal stages. (**B**) Gene-Concept Network (CNet) plot of the top 15 most significant (*P*_adjust_ < 0.05) associated GO biological processes to postnatal (*n* = 24) and perinatal (*n* = 4) consensus genes. Nodes for processes are colored beige. Node size represents the number of hits. Nodes for genes are colored red. Genes also present in the GWAS are marked with round brackets. Only genes that are involved with the top 15 GO biological processes are shown (14 of 28 genes). (**C**) Connectivity between SCZ risk genes defined at various windows of genomic extension and 22 non-GWAS postnatal consensus genes in an iPSC dataset. Plots show −log_10_(median gene-wise connectivity) (*y* axis) of consensus genes to each SCZ gene set (*x* axis) from an iPSC-derived gene coexpression network. A null distribution of −log_10_(median gene-wise connectivity) was computed from randomly permuted non-SCZ and nonconsensus genes, after matching for gene set size, GC content, gene length, and average expression (95% CI for the mean in shaded gray). (**D**) Sankey plot of consensus gene membership across age stages in the DLPFC. Thin nodes not connected by links represent modules containing 200-kbp SCZ risk genes but not consensus genes. (**E**) Median rank-normalized expression across sliding window samples by age for the four prenatal (top) and 24 postnatal (bottom) consensus genes in the NC DLPFC.

Table 1 shows genes coexpressed in SCZ risk modules in at least two-thirds of the networks that had at least one module enriched with SCZ risk genes in (i) cortical fetal samples [the perinatal age period–parsed network and the networks published in (*26*, *27*, *31*)] and (ii) postnatal networks [juvenile, adult, older adult DLPFC networks presented here, plus those reported by others (*5*, *10*–*14*, *35*)] (Fig. 9A). These “consensus” GWAS partner genes include four from the perinatal consensus (whereas 50 genes were found using the SCZ-negative gene list) and 24 genes in the SCZ list in the postnatal consensus (with none above the threshold in the negative list), all protein-coding genes. This finding suggests that including only four networks, as in the perinatal study, may be insufficient to disentangle biological signal from noise in a consensus analysis. The perinatal findings should thus be taken as exploratory.

**Table 1.** Consensus coexpression gene sets. Perinatal consensus considered four studies including fetal samples. Postnatal consensus considered 11 networks only including postnatal samples. Hits, the number of enriched modules each gene appeared in; total, the number of networks each gene was annotated in. Chr, chromosome; NA, not assessed; SCZ, lists of genomic extension windows including the gene among the top SCZ loci genes (distances in kbp); pLI, probability of loss-of-function intolerance (loss of function both with homozygous and heterozygous deleterious mutations); DEGs, SCZ differentially expressed genes ([+/−], over/underexpressed in patients with SCZ relative to NCs); DMGs, differentially methylated genes; *P* value, empirical *P* value obtained from permutation of consensus genes. References in the table: *[1]* (*5*), *[2]* (*91*), *[3]* (*94*), *[4]* (*90*), *[5]* (*93*), and *[6]* (*89*).

	Ensembl ID	Symbol	Chr	SCZ	pLI	MAGMA	H-MAGMA adult	H-MAGMA fetal	DEGs	DMGs	Hits/total (ratio)	*P* value
**Perinatal consensus**	**ENSG00000101204**	*CHRNA4*	20		0.021	2.13	1.49	4.08			3/4 (0.75)	1 × 10^−4^
	**ENSG00000119522**	*DENND1A*	9		0.90	1.41	1.11	1.45			3/4 (0.75)	1 × 10^−4^
	**ENSG00000162104**	*ADCY9*	16	500	0.97	2.15	0.96	1.44	*[1][−]*	*[2,3]*	3/4 (0.75)	2 × 10^−4^
	**ENSG00000173175**	*ADCY5*	3		0.99	−0.2	−0.26	−0.32		*[2]*	3/4 (0.75)	2 × 10^−4^
**Postnatal consensus**	**ENSG00000196588**	*MKL1*	22	0–500	0.54	2.59	4.76	4.58			8/10 (0.80)	2 × 10^−4^
	**ENSG00000198010**	*DLGAP2*	8	PGC	0.95	4.88	2.67	4.5		*[3,4]*	7/9 (0.78)	9 × 10^−4^
	**ENSG00000215012**	*RTL10*	22		5.8 × 10^−6^	0.88	1.11	1		*[2]*	6/8 (0.75)	5 × 10^−4^
	**ENSG00000002746**	*HECW1*	7		0.99	0.4	0.36	1.69		*[3,5]*	8/11 (0.73)	1 × 10^−4^
	**ENSG00000105649**	*RAB3A*	19		0.87	0.57	−0.1	−0.31		*[3]*	8/11 (0.73)	1 × 10^−4^
	**ENSG00000174437**	*ATP2A2*	12	PGC,0–500	0.99	6.02	6.05	6.22			8/11 (0.73)	2 × 10^−4^
	**ENSG00000175931**	*UBE2O*	17		0.99	2.83	0.99	0.79			8/11 (0.73)	2 × 10^−4^
	**ENSG00000184156**	*KCNQ3*	8		0.98	4.64	3.22	1.98		*[3]*	8/11 (0.73)	1 × 10^−4^
	**ENSG00000198929**	*NOS1AP*	1		0.12	0.28	1.01	0.67			8/11 (0.73)	2 × 10^−4^
	**ENSG00000084731**	*KIF3C*	2		0.91	1.31	3.24	1.9			7/10 (0.70)	4 × 10^−4^
	**ENSG00000130226**	*DPP6*	7		0.97	2.64	1.94	3.38		*[3,4]*	7/10 (0.70)	3 × 10^−4^
	**ENSG00000139767**	*SRRM4*	12		0.41	2.72	2.91	1.63		*[3,4]*	7/10 (0.70)	2 × 10^−4^
	**ENSG00000171435**	*KSR2*	12		0.99	2.62	4.23	4.31		*[3]*	7/10 (0.70)	2 × 10^−4^
	**ENSG00000178235**	*SLITRK1*	13		0.99	0.24	0.92	2.54			7/10 (0.70)	4 × 10^−4^
	**ENSG00000183715**	*OPCML*	11	PGC,0–500	0.58	6.3	2.78	0.03		*[2,3,4]*	7/10 (0.70)	3 × 10^−4^
	**ENSG00000241973**	*PI4KA*	22		0.0008	1.76	1.53	2.32	*[1][−]*		7/10 (0.70)	2 × 10^−4^
	**ENSG00000110427**	*KIAA1549L*	11		0.0037	0.8	1.4	−0.92			6/9 (0.67)	0.0012
	**ENSG00000133958**	*UNC79*	14		0.99	3.52	3.63	3.08			6/9 (0.67)	9 × 10^−4^
	**ENSG00000139182**	*CLSTN3*	12		0.99	2.35	0.7	1.87			6/9 (0.67)	0.0014
	**ENSG00000144406**	*UNC80*	2		0.14	−0.65	−0.14	0.05			6/9 (0.67)	6 × 10^−4^
	**ENSG00000145934**	*TENM2*	5		0.99	3.41	1.63	1.5			6/9 (0.67)	8 × 10^−4^
	**ENSG00000147724**	*FAM135B*	8		0.99	2.66	4.81	4.72			6/9 (0.67)	4 × 10^−4^
	**ENSG00000155093**	*PTPRN2*	7		0.017	2.41	0.64	0.17		*[2,3,4,6]*	6/9 (0.67)	0.0011
	**ENSG00000242732**	*RGAG4*	X		0.015	NA	NA	NA			6/9 (0.67)	0.001

In this full list of consensus genes, SCZ risk genes defined on the basis of PGC3 loci were a minority (18%). Only three genes of the 28 appeared among the 120 PGC3 prioritized genes (*DLGAP2*, *ATP2A2*, and *OPCML*). Therefore, genes not located in GWAS-significant loci are often coexpressed with PGC3 loci genes, more often than most PGC3 genes themselves. The lack of overlap between the consensus gene lists and GWAS loci genes aligns with the finding that SCZ risk genes shift coexpression partners over time. Although these genes were found coexpressed with SCZ risk genes only postnatally, most of them are expressed at higher levels in perinatal and juvenile than in adult life (Fig. 9E). We used H-MAGMA (*28*, *39*), which considers contact points in chromatin organization to map variants to genes within MAGMA, to characterize the fetal and adult risk score of each gene (Table 1). Gene ontology analyses on this list of 28 perinatal/postnatal genes point to the functions “chemical synaptic transmission” (seven hits, 5.6-fold enrichment, and *q*_FDR_ = 0.02), “cation transport” (eight hits, 4.7-fold enrichment, and *q*_FDR_ = 0.02), and “cAMP biosynthetic process” (two hits, 125.5-fold enrichment, and *q*_FDR_ = 0.02). The top associations of consensus genes with gene ontologies are represented in Fig. 9B.

We noticed that most of the 28 consensus genes have high probability of being loss-of-function intolerant. Loss-of-function intolerance is related to the depletion of disruptive variants. The most frequent coexpression partners of SCZ risk genes are more loss-of-function intolerant than background genes: *pLI* for SCZ risk genes averaged 0.49, whereas for consensus non-GWAS genes, it averaged 0.61; both estimates surpass those of all other protein-coding genes in the genome expressed across at least four perinatal or six postnatal networks (*N* = 9,552; background median *pLI* = 0.22; one-sample Wilcoxon signed-ranked test, *t*_30_ = 8.6, one-sided *P* = 0.006). High *pLI* is thought to characterize potential SCZ core genes (*11*), and frequent coexpression partners of SCZ risk genes share this feature. No transcription factors or microRNA (miRNA) targets were significantly overrepresented in the consensus sets.

We derived empirical *P* values for each consensus gene by permuting module membership 10,000 times and identifying a null distribution of consensus genes (see Table 1 and Materials and Methods). Each consensus gene had a higher probability of being coexpressed with SCZ risk genes across both perinatal and postnatal networks than expected by chance. Moreover, obtaining ≥4 (perinatal) or ≥24 (postnatal) consensus genes as output of this analysis by chance is unlikely for randomly selected genes (perinatal empirical *P* = 0.03; postnatal empirical *P* = 1 × 10^−4^).

We looked at the expression of consensus genes in DLPFC 10× single-cell sequencing data taken from (*41*) (https://github.com/LieberInstitute/10xPilot_snRNAseq-human), which has preannotated cell type and neuronal subtype clusters. Expression levels of consensus genes were consistently higher in neuronal cell types versus others (fig. S7). We then evaluated the environment around consensus genes for each network. For each network, we generated gene sets from the union of all module partners of consensus genes (even if that module was not SCZ enriched). Across all networks, consensus partners were very strongly enriched for SCZ risk genes, whereas the union of modules not including consensus genes was never enriched for SCZ risk genes despite this meta-set including more genes (meta_set_with_consensus median = 3086, SD = 1467; meta_set_without_consensus median = 6653, SD = 3470; fig. S8), indicating that consensus SCZ risk pathways may be dispersed across multiple SCZ risk modules and may not come out as statistically significant when compared to each other. The consensus partner set showed enrichments related to synapse and neuron (fig. S9A). Kyoto Encyclopedia of Genes and Genomes (KEGG) annotations revealed additional enrichments in circadian entrainment, oxytocin signaling, and addiction (fig. S9B). The flow of consensus genes across age mirrored closely the flow of SCZ risk genes, with several overlapping modules (Fig. 9D).

### Study 4: Consensus genes in iPSC-derived neurons

Last, we explored whether this consensus set of SCZ coexpression partners is coexpressed with SCZ risk genes in a cell model of early brain development. We analyzed RNA-seq from neurons derived from human iPSCs designed to have a wide separation of individual genetic risk for patients with SCZ compared with control samples (*33*). We found that 22 of the 23 non-GWAS consensus genes reported in Table 1 were also expressed in iPSC-derived neurons. We verified whether sets of putative SCZ risk genes (defined from PGC3 and at varying genomic windows from PGC3 SNPs) were highly connected with consensus genes (not including those already labeled as SCZ risk genes). We found that, even in these stem cell–derived neurons, the preferential connectivity pattern of consensus genes with putative SCZ risk genes is preserved and remains significantly higher than background genes matched for length, GC content, and average expression (Fig. 9C).

## DISCUSSION

We investigated the convergence of genetic risk for SCZ into coexpression networks at different periods of the neurotypical life span across different brain regions to address how SCZ risk genes change their coexpression partners across age and to identify a molecular environment that might be particularly relevant to enabling the pathogenic effects of genetic risk. The evaluation of GWAS top loci and whole-genome risk yielded consistent results regarding a profile of convergence of SCZ risk on early development coexpression patterns in the DLPFC, followed by the juvenile hippocampus and caudate nucleus convergence. The key findings of this report are as follows:

1) Parsing by age periods explained more variance in a score of gene importance for SCZ compared to lumping all age periods together in a single network.

2) SCZ risk genes tend to be more coexpressed than random genes matched for potential confounders, and cluster into specific modules, especially in early-stage DLPFC. The finding that perinatal and juvenile networks showed greater maximum overrepresentation relative to adult networks suggests that SCZ risk genes cluster best into coexpression networks in younger neurotypical brain tissue.

3) SCZ risk genes tend to continue to be clustered together from perinatal to juvenile life in DLPFC, but most of their coexpression partners change. This finding was replicated when using exclusively prenatal samples and was further replicated in external datasets, suggesting that these SCZ risk genes continue to exert combined effects while part of their molecular environment changes across development, potentially underlying the age-associated shifts in clinical symptomatology.

4) Networks derived from the caudate nucleus of patients with SCZ show greater convergence of SCZ risk genes than networks from age-matched NC. This effect is not explained by cell type abundance inferred via deconvolution.

5) Despite age changes in coexpression, a set of 28 genes is reliably found in modules enriched for SCZ risk genes in the DLPFC across multiple publications, yet 82% of these genes are not in GWAS-significant SCZ loci. These non-GWAS enrichment-related genes are at least as intolerant to loss-of-function mutations as GWAS genes and significantly more so than the background and may represent a consistent molecular environment that mediates SCZ risk.

6) Most of these consensus genes are highly expressed in perinatal life and are also expressed and correlated with SCZ risk genes in iPSC-derived human neurons. This evidence suggests an in vitro platform for experimental investigation of their role in enabling SCZ biological risk.

Together, these findings suggest that mRNA coexpression shows a convergence of SCZ genetic risk situated in the context of age period and brain regions specificity, revealing potentially important biological signal beyond what has been identified with GWAS.

### SCZ gene coexpression changes across the neurotypical life span

The aim to identify when and where in the brain SCZ risk genes converge is based on the putative role of neurodevelopment in SCZ. These results emphasize early convergence of SCZ risk and potentially shed light on its shifting clinical presentation (*42*–*44*). Recent evidence supports this view. SCZ candidate risk genes are expressed earlier in humans than nonhuman primates, especially during prenatal development (*45*). Early life gene expression and chromatin accessibility are associated with SCZ genetic risk (*26*, *46*, *47*); SCZ genetic risk shows higher penetrance in the presence of perinatal environmental risk (*48*); combined GWAS/chromatin contact analyses support early life genetic risk convergence for psychiatric disorders (*28*). Most recently, Cameron *et al.* (*24*) have shown that genes with high PGC3 associations, as estimated via MAGMA, are preferentially expressed in developing neurons of the frontal cortex during the second trimester of gestation.

Our data suggest that assessing coexpression changes across the life span identifies brain region specificity, which is not equally accessible with “nonparsed” coexpression approaches. For example, SCZ risk modules enriched for the targetomes of *SP4*, a transcription factor prioritized in genetic associations with SCZ (*2*, *40*), are found in the perinatal and juvenile DLPFC but not in any nonparsed network (Fig. 6A). Consistent with the privileged role of the DLPFC in genetic risk for SCZ, we found that in this region and not in the hippocampus, SCZ risk genes in perinatal life were still coexpressed in juvenile individuals, while changing their coexpression environment partners. SCZ risk genes were peripheral in hippocampal perinatal networks, and the enriched modules overrepresented risk for other disorders and not just SCZ, effects not observed in early-stage DLPFC. Note that our benchmark networks identified using the same samples but without parsing did incorporate age information. As in prior studies, age could be traced back to the first principal component of module gene expression (*27*, *31*). However, the aggregation of genes in prior studies and in our benchmark networks was likely driven by gene expression changes during development, without the opportunity to identify how coexpression partners change during the life span. In particular, the neurotypical function of gene modules associated with SCZ appears more fundamental during perinatal and early developmental periods. As most of the evidence currently available about SCZ genetic risk in coexpression networks derives from adults, the developmental perspective that we provide here has likely been missed in previous work. The network resource developed in this work is freely available and can be used for further studies.

Several of our findings are relevant to a system neuroscience approach to SCZ. The privileged position of the DLPFC in the functional genomics of SCZ agrees with many prior postmortem DLPFC and imaging studies of living individuals (*49*–*51*). As previous studies established for gene expression (*26*, *27*, *29*, *30*), also for coexpression, there are differences between prenatal and postnatal DLPFC. Our results support this idea as well, but to a lesser degree, for hippocampus. However, we also found that RNA-seq from the dentate gyrus of the hippocampus showed greater coexpression of SCZ risk genes than bulk tissue and peak coexpression in NC in the sliding window analysis, supporting the relevance of SCZ risk gene convergence for neurons and suggesting cell type beyond brain region specificity. A convergence of SCZ risk genes on neuronal and especially synaptic biology is the same conclusion reached in the PGC3 report (*2*). To the extent that coexpression recapitulates cell types, cellular districts, and functional pathways, the genes most often coexpressed with SCZ risk genes should share their main functions and, in fact, do so. The additional enrichment analyses also highlight spatiotemporal aspects of SCZ risk gene coexpression. For instance, differentially methylated CpG islands (DMGs) are coexpressed principally in perinatal or juvenile networks; and differentially expressed genes (DEGs) are coexpressed in the caudate nucleus. The caudate nucleus SCZ enrichment time course aligns with dopaminergic maturation (*37*, *44*). In the sliding window analysis, patients with SCZ generally showed greater enrichment for SCZ risk genes in caudate nucleus networks, the brain region with maximum dopaminergic innervation. This finding may be associated with D_2_ receptor–targeting antipsychotic treatment in patients with SCZ or with the impact of variants in genes relevant to dopaminergic transmission (*52*). Notably, our results based on a connectivity match between networks across brain regions and age failed to support previous findings of less connected coexpression networks in the DLPFC of patients with SCZ (*5*). We hypothesize that the networks available with the present work may afford greater explanatory power in the translation of postmortem brain insights into neuroscience and clinical predictions in living patients with the latest available approaches (*13*–*15*, *53*–*60*).

### The developmental milieu of SCZ risk genes

Arguably, our most unexpected and novel result is the identification of a set of mutation intolerant consensus genes that are especially likely to be coexpressed with SCZ risk genes. This set of genes is coexpressed with SCZ genes in the large majority of the networks that we examined, regardless of often emphasized differences across datasets, preprocessing pipelines, and parameter setting. The method that we used to derive this consensus is very conservative, as confirmed by the statistical significance of finding very large consensus sets. The gene-wise *P* values that we derived control for bias related to module size of the consensus genes and are highly significant for all genes.

We successfully predicted on the basis of the postmortem data that these genes would be more coexpressed than matched background genes with the set of 120 PGC prioritized genes in iPSC-derived human neurons. There is no obvious clustering of these genes by chromosome, hence discounting the possibility of locus-related artifacts. By merging modules containing consensus genes, we showed strong enrichment for SCZ risk genes, which was not found when merging modules without consensus genes. Gene ontologies for the consensus gene set and their modules once again emphasized neurons, synapses, and ion transport. It is tempting to speculate that these genes mediate at least in part the read-out of SCZ risk at the level of biological systems and that they are involved in translating SCZ genetic risk into SCZ biological risk.

There are multiple nonexclusive explanations as to why genetic variants relevant to the biology of a heritable disease might not show GWAS-associated signal, which may be related to their high loss-of-function intolerance. For example, they might be depleted in common cis-functional polymorphisms; their common variants may be associated with other disorders that represent exclusion criteria for SCZ genetic studies, e.g., other central nervous system disorders; their effect may be nonadditive, for instance, because of epistasis with other variants masking their effect in GWAS, or their expression may be principally regulated by trans-eQTLs. Gene coexpression provides a handle on the role of potentially relevant genes undetected by GWAS, thus highlighting previously unreported targets “guilty by association” and related regulatory elements. These non-GWAS genes thus may be part of the cellular environment in which GWAS-positive genes exert their pathogenetic function, potentially accounting at least in part for how diverse GWAS risk genes of small clinical effect converge on a common phenotype that shows varying expressivity over the life span.

In terms of limitations of our methods, although we analyzed the largest developmental series available to date, the absolute cohort sizes are still limited, especially for perinatal and juvenile networks. We endeavored to mitigate this shortcoming by including a minimum of 40 samples per network; however, this choice came at the cost of diluting biological specificity associated with age by extending the age intervals beyond the desirable limits, e.g., up to 6 years of age in the perinatal window. The networks identified also depend on the nonuniform age sampling with fewer samples between 1 and 20 years. For these reasons, we confirmed findings pertaining to perinatal and juvenile windows in independent RNA-seq data and also included a sliding window approach, which supported the conclusions of the fixed age period study.

A relevant methodological aspect of this work is that we obtained networks comparable across the life span via statistical corrections for cell type abundance and by matching connectivity between different brain regions and age periods. These operations reduce the possibility that these potential confounding variables drive the findings. Cell specificity enrichments computed on SCZ-enriched modules showed that removing interindividual variation in cell abundance estimates did not compromise module specificity, which appears equal to or greater than those of published reports. Despite our efforts, some confounding effects may still affect the results, particularly regarding ancestry and sex. Statistical correction of the effects of these variables on expression data cannot address the potential different genetic architecture underlying the expression of SCZ risk genes across ancestry groups and sex. The population in our cohort differed from the reference PGC3 GWAS that we used to identify SCZ risk genes. It is thus possible that these variables still affect our findings, although no evidence has been reported of differential genome-wide association with SCZ in males and females (*2*).

In conclusion, while the convergence of SCZ risk genes into coexpression pathways has been observed before, here, we identify spatiotemporal dimensions of this convergence such that the relationships between genes change along the life span, especially between perinatal and postnatal life, perhaps in parallel to the seminal transition from the preclinical to the manifest illness. Age-parsed networks afford superior predictions in terms of potential gene association with SCZ. The granular quantification of SCZ risk gene convergence across age reveals a difference between neurotypical adults and patients with SCZ specifically in caudate nucleus data. Most notably, SCZ risk genes converge especially in perinatal and juvenile DLPFC, as shown by enrichment and prediction analyses, and keep being coexpressed in a shifting context. Together, these findings highlight that the genetic architecture of SCZ is embedded in shifting coexpression patterns across brain regions over the life span. Leveraging this shifting pattern of coexpression, we identified novel associations with SCZ in a set of genes consistently coexpressed with GWAS-positive genes that may be part of the cellular environment that mediates the translation of genetic risk into biological risk for SCZ, an environment that can be readily studied in an in vitro model to investigate the potential contribution of these genes for the development of future treatments.

## MATERIALS AND METHODS

### Subjects and materials

This study was based on brain tissue of NC and individuals diagnosed with SCZ of European or African American ancestry, all with RNA integrity number (RIN) ≥ 6 (table S1). All tissue donations were made with informed consent from next of kin. A number of brains in the LIBD Human Brain Repository were transferred from the National Institute of Mental Health (NIMH) Clinical Brain Disorders Branch under a material transfer agreement, after having been collected under NIMH Protocol 90-M-0142 and processed and approved by the NIMH/National Institutes of Health Institutional Review Board. Additional cases in the LIBD repository were collected from the Office of the Chief Medical Examiner for the State of Maryland under State of Maryland Department of Health and Mental Hygiene Protocol 12-24 and from the Kalamazoo County Michigan Medical Examiners’ Office under Western Institutional Review Board Protocol 20111080. All samples were collected and processed using a standardized protocol specifically developed to minimize sample heterogeneity and technical artifacts. All included NC subjects had minimal age-associated neuropathology (determined from postmortem histopathological examination) and no substance or drug use from toxicology and were free from any psychiatric or neurological disorder from clinical histories. Postmortem clinical information was gathered by conducting family interviews with the next of kin, as previously described (*61*). After psychiatric record reviews and postmortem family interviews were completed, brief psychiatric narratives were prepared on each case, summarizing the demographic, clinical, medical, and death information obtained from as many sources as possible (i.e., multiple psychiatric records, police reports, neuropathology reports, medical examiner’s information, toxicology screen, and postmortem family interview). Each case was then independently reviewed by two board-certified psychiatrists, who arrived at consensus DSM-IV Axis I lifetime diagnoses or consulted with a third reviewer to reach a final diagnosis.

DLPFC samples were derived from Brodmann area 9/46 at the level of the rostrum of corpus callosum. Hippocampus samples were derived from the mid-hippocampus proper (all dissections included the dentate gyrus, CA3, CA2, and CA1) plus the subicular complex (*61*). For caudate nucleus, samples were taken from the anterior “head” portion, which is the caudate part most tightly connected with the prefrontal cortex. Dentate gyrus samples were obtained from the granule cell layer of the dentate gyrus (*DG-GCL*). *DG-GCL* samples were isolated from neighboring polymorphic and molecular layers by using laser capture microdissection (*23*). For all tissues, RNA-seq was performed via the Illumina Ribo-Zero Kit. In the Age study, we determined the age periods as a trade-off between achieving a minimum sample size for each age period to identify networks (minimum of 40 subjects) and the identification of biologically meaningful age windows.

#### Replication datasets

The replication study was carried out using the (i) UCLA-ASD data from the PsychENCODE collection (*36*); here, we selected 16 NC ranging in age from 6 to 25 years; (ii) BrainSpan data from 21 perinatal and 9 juvenile samples; and (iii) PolyA data from the LIBD repository, not overlapping the LIBD samples already used in the main analysis, from 29 perinatal and 19 juvenile individuals.

### Quantification and statistical analysis

#### Data preprocessing

All downstream analyses have been performed primarily using the R statistical software (version 4.0.0+). Specific R packages used are also listed in the Key Resource Table in the Supplementary Materials.

#### Regional coexpression and age studies

We performed a common preprocessing for non–age-parsed tissue samples to ensure the use of the same samples and settings across ages (the only difference is, thus, the subsequent age parsing). Computing preprocessing parameters on the entire cohorts also allowed the best-informed estimation of confounding effects on a large sample. Specific challenges in this approach concern the preservation of age effects during preprocessing, the comparability between the networks obtained, and the variation of cell population abundance between early development and adulthood.

Gene-level mRNA expression was quantified as reads per kilobase per million mapped reads (RPKMs) and annotated as total gene expression separately for each brain region, regardless of alternative transcript quantification (*61*) using GENCODE release 25 (GRCh38.p7). We selected genes above median RPKM ≥ 0.1 and free of floor effects (maximum 20% of zeroes per gene); we log-transformed RPKM values with an offset of 1, i.e., log_2_(RPKM + 1). We used inter-array distance to identify outlier subjects deviating more than 3 SDs from the mean (*14*). Outliers were identified relative to their age period in the fixed period analysis. Outliers identified in this step were also excluded from the subsequent sliding windows analysis. After removing mitochondrial genes that were highly expressed in some samples, datasets included a variable number of genes for different regions (table S1).

Comparing networks across different age periods requires the preservation of the biological effects of age while removing unwanted confounding effects. Standard noise removal techniques, such as principal components analysis, are effective at modeling noise into latent factors while preserving biological effects (*62*, *63*). However, these latent variables were strongly associated with age in our samples (>80% shared variance), likely because of the inclusion of prenatal samples. This finding extended to latent variables derived from experimental quantification of degradation effects conducted on adult samples, i.e., qSVA (quantitative surrogate variable analysis) (*64*). To reduce the risk of removing age-related signal in the Age study, we used correction for explicit confounders, a practice that has been suggested as optimal for gene coexpression networks identified in brain tissue (*11*). In particular, we regressed out the effect of the following variables from each brain region cohort separately on the basis of availability: Confounding variables regressed out for BrainSpan were sex, self-reported ancestry, and RIN. Confounding variables regressed out for UCLA-ASD were sex and RIN. Confounders used for PolyA were the same as those used for the primary LIBD networks, namely, sex, mitochondrial mapping rate, ribosomal RNA (rRNA) rate, gene mapping rate, RIN, ancestry estimated via the first 10 genomic eigenvariates [computed as in the work of Collado-Torres *et al.* (*61*)], and estimated individual neuronal proportion; the latter confounder was included to obtain an appropriate comparison between subjects of different age. Individual-level estimated neuronal proportion was computed using the R package BRETIGEA (species: human, scaling: FALSE, dataset: kelley) (*65*) and regressed out because it could bias estimated gene-gene connectivity due to interindividual variation in cell abundance across age periods. We estimated and protected linear and quadratic terms of age using the function cleaningY from the Jaffelab package (*66*). We rank-normalized residuals using Blom formula (*13*, *54*, *56*, *58*) to limit the impact of deviations from normality in expression data (*67*).

#### Network identification across all studies

Blom-normalized residuals obtained using the linear models described above were entered as input in the blockwiseModules function from the package WGCNA to construct “signed hybrid” network(s), i.e., negative correlation were set to zero and positive correlations were soft-thresholded. We obtained the similarity matrix using Pearson’s R correlation index (minModuleSize: 40, maxBlockSize: 3000, deepSplit: 4, mergeCutHeight: 0.15, pamStage: TRUE, reassignThreshold: 1e-06). The parameter used for soft-thresholding is the exponent β to which the correlation matrix is raised to obtain the adjacency matrix. The standard procedure is to pick the lowest possible β that is high enough to satisfy the scale invariance criterion, defined as the *R*^2^ > 0.8 in the correlation between original and log-transformed values (*34*). Lower β values are often associated with greater network median connectivity. For each network, we selected the parameter β such that connectivity was matched across all networks (see below; table S3).

Exploratory analyses on our datasets revealed that perinatal networks yielded systematically greater connectivity than other age periods, thus undermining comparisons between brain regions and time windows. Outcome variables such as the number of nonclustered genes are influenced by β and by network connectivity; thus, the standard criterion to identify networks generated large differences between networks. To obtain comparable networks, we assessed the median network connectivity for each sample and selected β to meet the scale invariance requirement in all networks while also matching connectivity. To achieve this effect, we used larger β values for early, highly connected networks at a level of connectivity compatible with later life-span periods while still meeting scale invariance. Following this procedure, we obtained comparable graph structures with small variations in connectivity and in number of nonclustered genes (fig. S10).

For each replication dataset, we rank-transformed and normalized expression values to construct a network for each age group. For the construction of networks, genes were matched between all sets and with the main networks that we generated (*n* genes = 14,553). A soft power (β) was first identified for each such that the network was scale invariant (scale invariant index > 0.8) and the median connectivity is not greater than 1.123 (the median connectivity across LIBD network). The BrainSpan juvenile network did not match these criteria under β = 30 (the maximum allowed by the WGCNA package) and was thus not used further. The blockwiseConsensusModules function from the WGCNA package was used to detect consensus modules across perinatal networks polyA_Perinatal (β = 17) and Brainspan_Perinatal (β = 29) and across juvenile networks polyA_Juvenile (β = 22) and UCLA_Juvenile (β = 25).

Other previously published networks were used “as is,” except for the network published by Pergola *et al.* (*13*). This network used BrainCloud, a microarray dataset featuring constitutive and alternative probes. Therefore, we reannotated it to obtain a total gene expression quantification and make it comparable with the other networks, which were all annotated to GENCODE release 25 (GRCh38.p7) to be consistent with the annotation database used in the network identification. For each probeID, first, the corresponding accession number was converted into Ensembl ID using clusterprofiler package (*68*). When an accession number mapped to multiple Ensembl IDs, a single Ensembl ID was randomly selected. If no Ensembl ID was returned via accession number, then the gene symbol corresponding to the probeID was used to derive an Ensembl ID. Only one probeID per Ensembl ID was retained in a preferential order of probe types: constitutive exonic (hHC), then alternative exonic (hHA), and then human mRNA (hHR).

#### Network characterization and association with SCZ risk

##### Definition of SCZ risk genes

We used the PGC3 definition of 120 priority genes as the list called “PGC.” Then, to obtain genomic regions agnostic of PGC3 fine mapping and reflecting a consensus between SCZ risk gene lists, we evaluated enrichment at multiple thresholds of genomic distance from the index PGC3 GWAS-significant SNP. We have previously tested a similar method in another dataset using PGC2 GWAS-significant loci (*14*). The rationale for this choice was to generate lists of genes in the proximity of significant SNPs that survived genome-wide correction for multiple comparisons, assuming that in these genetic regions, the probability of finding causative genes is highest. On the other hand, as we do not know whether the index SNP is causative and gene regulation is also possible at a certain distance from the genes, we evaluated increasing extension thresholds to include increasing numbers of genes. The number of genes included was still much smaller than all genes included in all loci identified by PGC3, which are often larger than 1 million base pairs (Mbp) and thus are proximal to thousands of genes (possibly with the dilution of the probability that each gene is causative). By assessing the consensus between gene lists, we attempted to use a conservative approach in which the choice of parameters does not drive results. We considered nine extension windows at increasing distances in both directions from each PGC3 GWAS significant SNP: PGC [120 genes], 0 kbp [178 genes] (meaning, genes that overlapped with the index SNP), 20 kbp [299 genes] [extension from the loci used in PGC2 (*69*)], 50 kbp [456 genes] [extension from the loci used in PGC3 (*2*)], 100 kbp [705 genes], 150 kbp [963 genes], 200 kbp [1196 genes], 250 kbp [1436 genes], 500 kbp [2475 genes] [500 kbp was the maximum extension where enrichments were found significant by (*14*)].

The ancestry composition of our expression data included European and African American individuals, hence different from the *PGC* core cohort (130,644 European and 30,761 Asian ancestry individuals), as well as from the *PGC* primary cohort including European, Asian, African, and Latino ancestries (*2*). The addition of African and Latino ancestry in the *PGC* primary results has modest effects on the overall summary statistics because the sample size is below 10% of the *PGC* core dataset but has a sizable effect on the clumping, arguably because ancestry in the genome largely takes the form of LD blocks. As the purpose of our analysis was only to identify index SNPs associated with SCZ and not for genetic trait associations, we based our definition of risk genes on the *PGC* core summary statistics (*2*). These summary statistics are well powered, genetically more homogeneous than the *PGC* primary results, and largely consistent with previous SCZ-GWAS. It is also noteworthy that the sex distribution in our expression data privileged males over females, representing another difference from the *PGC* core cohort (female-to-male ratio = 0.81), in which the overrepresentation of males was less overwhelming. A chi-square test on the proportion of males and females returned a highly significant difference [χ2 (df = 1, *N* = 164,061) = 34, *P* = 5.5 × 10^−9^].

We generated negative control gene sets equal in size to the ones described above used in the enrichment analysis by selecting the 500 least significant SNPs in the PGC3 core summary statistics and defining negative loci at the above-considered distances from each tagging negative SNP. We excluded any region located at ±3 Mbp from SCZ SNPs from this selection to rule out any overlaps between positive and negative gene sets.

#### Coexpression of SCZ risk genes regardless of module membership

To test the hypothesis that putative SCZ risk genes are more highly coexpressed than expected by chance, we took as connectivity measure the median of the gene-wise mean connectivity with all other SCZ risk genes from the adjacency matrix of each network identified and compared it to a null distribution of randomly permuted non-SCZ genes.

As GC gene content and gene length may bias random permutations [SCZ risk genes are GC richer and longer than random genes (*2*)] together with gene expression within each network, we ran 10,000 permutations with the restriction to match both gene set size and GC content, besides gene length and average expression distributions of the SCZ risk genes. The universe from which random genes were pooled consisted of genes expressed in each network, excluding SCZ risk genes (the most extensive list that we generated at 500 kbp from GWAS-significant SNPs was considered for this analysis). Last, we defined the empirical *P* value as the number of occurrences in which the connectivity measure of the random genes exceeded the one of the SCZ risk genes divided by the number of permutations. We set the significance threshold at an empirical *P* value < 0.001. We repeated this procedure for the nine extension windows previously created.

#### MAGMA

To calculate MAGMA and H-MAGMA, we used the MAGMA tool v1.09b (*39*), PGC3 summary statistics as SNP *P* value data (*2*) and 1000G European as the reference data file for a European ancestry population to estimate linkage disequilibrium between SNPs. We took the following steps: (i) We mapped 1000G SNPs to genes encompassed in each network module [a window of 35 kb upstream and 10 kb downstream of each gene; for H-MAGMA, we used adult brain Hi-C annotation files already computed in the H-MAGMA publication (*28*)], (ii) calculated gene *P* values on the basis of PGC3 SNPs *P* values, and (iii) performed gene set enrichment analysis using the modules detected in the network that assigns a gene set *P* value (universe used by MAGMA consists of all network genes with at least one SNP mapped). We computed this analysis for each network for both all biotypes and protein-coding genes and corrected for the number of modules in each network using Bonferroni’s rule.

### Enrichment methods

We identified modules in each network. Then, we assessed the overrepresentation of SCZ risk genes for each module except gray in each network relative to a universe composed of all non-gray genes. We corrected results for multiple comparisons via Bonferroni (number of non-gray modules in each network). This statistic is independent of the underrepresentation in the gray module and represents the competitive enrichment of one module relative to the background. We labeled as SCZ risk modules those significantly enriched in at least three of the nine extension windows (hypergeometric test, Bonferroni-corrected *P* value <0.05). In addition to hypergeometric tests, we also computed permutation statistics (creating for each module in each network a null distribution of 10,000 sets of randomly sampled genes using network-specific genes as the universe) to obtain an unbiased empirical *P* value. We performed these same analyses in networks from published reports to provide a benchmark (*5*, *10*–*14*, *26*, *27*, *31*, *35*, *36*). Moreover, we tested whether SCZ risk genes were significantly underrepresented in the gray module of nonclustered genes relative to a universe represented by all genes expressed in a network. Significant underrepresentation suggests that SCZ risk genes tend to be coexpressed rather than not (see results in fig. S2).

Using the same methods with other GWAS summary statistics, we computed gene enrichment for several psychiatric, neurologic, and immune disorders [attention deficit hyperactivity disorder (*70*), autism spectrum disorder (*71*), bipolar disorder (*72*), major depressive disorder (*73*), obsessive compulsive disorder (*74*), posttraumatic stress disorder (*75*), suicide attempt (*76*), Alzheimer’s disease (*77*), amyotrophic lateral sclerosis (*78*), multiple sclerosis (*79*), Parkinson’s disease (*80*), Crohn’s disease and ulcerative colitis (*81*), and rheumatoid arthritis (*82*)].

To mitigate the risk of biased enrichments due to the inclusion of many genes mapping onto few loci, which may depend on RNA-seq read annotation for transcripts overlapping in the DNA sequence or due to common regulatory sequence elements between close genes, we also performed a permutation test of loci overrepresentation. In this test, all genes within a module located in the same locus (defined according to the genes located in the proximity of GWAS-significant SNPs) were counted as a single hit.

For the cell type enrichment analysis, we used gene sets reported by Skene and colleagues (*83*) including cell type specificity indices available at www.hjerling-leffler-lab.org/data/scz_singlecell/. They computed specificity indices for each gene and range between 1 (high specificity for a given cell type) and 0 (low specificity). We used specificity indices derived from single-nuclei RNA-seq of human brains [DroNc-seq in prefrontal cortex and hippocampus (*84*)]. We used mean-rank gene set test in the limma R package (*85*) to evaluate the enrichment of our components for the specificity indices of each cell type.

We characterized the modules of interest using the Gene Ontology Database (PANTHER) (*86*) and clusterProfiler package (annotation package: org.Hs.eg.db 3.16). We also computed enrichments for cell specificity (*83*); transcriptome-wide association study (TWAS) genes obtained from caudate nucleus (*52*), hippocampus (*61*), and DLPFC data (*7*, *61*, *87*); DEGs obtained from caudate nucleus (*52*), hippocampus, and DLPFC data (*61*); DEGs in each brain region (*5*, *6*, *52*, *61*, *88*), genes proximal to differentially methylated CpG islands (DMGs) in PFC, and blood (*89*–*94*); DEGs in humans relative to great apes (*88*); SCZ drug target genes (*95*–*98*); and loss-of-function intolerant genes (*99*) in the selected modules. For TWAS and DEGs, we performed a brain region–specific enrichment using the appropriate gene list of each tissue. The “universe” of TWAS enrichment was adjusted by intersecting the list of genes associated with a cis-eQTL, called heritable genes, with the brain region–specific list of expressed genes. Moreover, for DLPFC TWAS and DMGs, as well as for drug target enrichment, we computed a meta-analysis of the papers that we retrieved target genes from to obtain module-wise enrichment *P* values (sum-log Fisher’s method, agnostic to developmental stage).

#### MAGMA linear models

To reduce biases associated with genes uniquely expressed in a single brain region or age period, in this analysis, we only considered autosomal genes included in all Age study networks, i.e., the intersection of genes expressed in the DLPFC, hippocampus, and caudate nucleus. In particular, we performed the gene analysis using MAGMA annotation files (35-kb upstream and 10-kb downstream window was used), PGC3 summary statistics as SNP *P* value data, and 1000G European as the reference data file for a European ancestry population, as reported above. Thus, we considered the gene-wise *z* scores obtained in this analysis as the MAGMA importance score (i.e., the measure of gene annotation to SCZ) and assessed the association of coexpression in different brain regions and age periods with gene importance for SCZ derived by MAGMA. Because H-MAGMA Hi-C annotation was performed by authors of the paper using hg19/GRCh37 as genomic reference, we only kept SNP and genes locations consistent with H-MAGMA reference when generating H-MAGMA scores for the consensus genes.

We computed linear models to associate this score with coexpression. In these analyses, the outcome variables were represented by the MAGMA importance score obtained for each gene, previously marginalized by gene position, number of transcripts, GC content, and gene. We assessed the improvement in model fit deriving from the inclusion of coexpression variables in the model relative to a null model only, including confounders, via maximum likelihood estimation. This analysis implied an analysis of variance (ANOVA) comparing the null model with the test model. We then tested the following hypotheses: (i) Age-parsed networks explain more variance in gene importance for SCZ than using the same samples to compute a single network per tissue; we tested this hypothesis with the Vuong test for non-nested linear models (comparing full sample versus age-parsed networks). (ii) SCZ risk convergence is region and time specific, i.e., networks computed at critical age periods are reproducibly related to SCZ risk importance.

We considered as confounders chromosome location, gene start (to index location within the chromosome), gene width, GC content, strand, number of isoforms, and gene type. These variables are potentially related to SCZ risk importance, but they do not necessarily reflect coexpression-related signal, which is why their biological association with SCZ is considered confounding here. The poorly represented gene types in our dataset were excluded to avoid biasing the models; the minimum WGCNA module size served as the threshold for exclusion (40). We thus excluded genes annotated as 3prime_overlapping_ncRNA (4), polymorphic_pseudogene (3), IG_V_gene (2), non_coding (1), rybozime (2), scaRNA (15), scRNA (1), TR_V_pseudogene (1), transcribed_unitary_pseudogene (15), and unitary_pseudogene (15). The remaining gene types had over 41 entries each. The dataset included 17,996 genes.

To test the second hypothesis, we compared prediction performances for the same machine learning workflow on parsed and nonparsed datasets. The two datasets differed in expression attributes and coexpression properties, specifically median gene expression (3 features in nonparsed and 11 in age-parsed) and kTotal (connectivity of a gene to all other genes in the network) and KME (module membership of a gene) connectivity variables for the modules of the coexpression networks considered (3 kTotal + 146 KME features in nonparsed and 11 kTotal + 409 KME features in age-parsed). The workflow of the analysis is described in Fig. 4A and in detail in the methods S2.2. Briefly, we used a cross-validation approach stratified by chromosome location to identify statistically important features for each gene using Boruta’s algorithm (*100*). Such features were then used to train an XGBoost regressor and evaluate its performance using *R*^2^. Reiterating the pipeline for 200 runs of fivefold stratified cross-validation resulted in a distribution of 1000 *R*^2^ values (Fig. 4B). Figure 4 (C and D) shows the features selected by Boruta in the absolute majority of runs for the two datasets. The dataset included 21,751 genes because the algorithm was able to handle missing entries.

#### SCZ risk gene coexpression within-set connectivity

We subset the original adjacency matrix for each SCZ risk gene set to include only the in-set genes. For each gene in a set, we then computed the mean connectivity (connectivity having been defined as gene coexpression raised to an exponent β) to all other in-set genes. Last, the median of the gene-wise mean connectivity was computed for each set, as a measure of set-wise within set gene connectivity/coexpression. Connectivity values were compared to a null distribution of gene sets of equal set size, gene length, GC content, and average expression.

#### Risk gene flow

We constructed Sankey plots illustrating the flow of risk across ages. We considered the extension threshold of 200 kbp because it captured most of the enriched modules compared to all other extension windows (Fig. 5). We studied the composition of gray modules of nonclustered genes and of SCZ risk modules obtained considering all biotypes, not just protein-coding genes, to maintain the networks comparable with each other without using multiple criteria (DLPFC perinatal black, juvenile blue, adult pink, older adult greenyellow; hippocampal perinatal red, juvenile turquoise, and adult turquoise and brown). For additional results about the composition and ontologies of risk gene flow, see the Supplementary Materials (note S1.5).

#### JI analyses

In the analysis of module continuity across age periods, JIs were computed as the intersection/union of the considered sets. To draw statistical inferences on the dissimilarity between gray modules across age periods, we pooled all genes that at least in one of the age-parsed networks belonged to the gray module and resampled them 10,000 times to create a null distribution for each network. Then, using the earlier network as a reference, we computed a distribution of JIs with the null distribution and an empirical *P* value. Figure 7C thus represents the null distribution of JIs for each transition, holding the earlier network as a reference.

### Sliding windows

In the work outlined above, we examined age periods that necessarily include quite diverse biological processes. For example, the perinatal window ranged up to 6 years of age, thus reflecting different biology compared with fetal samples (although only a handful of samples were available between 0 and 6 years). In addition, keeping age windows fixed does not allow us to estimate to what extent results are an attribute of the age period considered and not an attribute of the specific samples analyzed or rather of sample size available. Furthermore, in the context of the age study, a comparison between patients and controls cannot be made because diagnosed patients are adults. Study 2 (sliding windows) was therefore aimed at addressing these points in two main steps. The first consisted in ruling out that the findings about convergence of SCZ risk genes across the life span are related to the specific samples analyzed. The second was to obtain a comparison between patients and controls. We identified networks in chronological sequential windows of 40 subjects to control for these potential confounding effects. This way, we could observe the continuous variation of parameters of interest (like fold-enrichment mean and variance and others) across different tissues. For this analysis, we added patients with SCZ to the previously used neurotypical individuals and dentate gyrus data (table S1).

We first compared neuronal proportion estimates between patients with SCZ and controls. Neuronal proportion was estimated using the BRETIGEA package using the BRETIGEA::brainCells function [BRETIGEA parameters: nmarkers = 50 (default) and method = “svd” (default), species = “human,” scaling = “F”] and then marginalized by total assigned genes, RNA_rate, and mitoRate. Residualized neuronal proportions were compared between age-matched diagnoses for each region using two-sided *t* tests. Neuronal proportion estimates were significantly lower in patients with SCZ than in controls across all regions except the caudate (CN *t*_264_ = −1.07, *P* = 0.284; DG *t*_132_ = 4.27, *P* = 0.000038; DLPFC *t*_287_ = 3.74, *P* = 0.00022; HP *t*_243_ = 3.53, *P* = 0.00049).

To compute sliding window networks, we sorted subjects by increasing age for each tissue and identified the first network with the 1st to 40th subjects, the second with the 2nd to 41st, and so on. We examined primarily the extension window at 200 kbp that in the age period analyses provided the strongest convergence (Fig. 5). For plotting, we used the ggplot2 package in R with smoothing method “loess” and span parameter 0.5, i.e., using 50% of the points to fit the local regressions (for dentate, the span parameter was 0.65 to accommodate for the lower number of windows compared to other tissues). For caudate NC windows, the first window is not plotted, as it is far from the second window in terms of median age.

Gene-level mRNA expression was quantified as RPKMs and annotated as total gene expression separately for each brain region, regardless of alternative transcript quantification (*61*) using GENCODE release 25 (GRCh38.p7). We selected genes above median RPKM ≥ 0.1 and free of floor effects (maximum 20% of zeroes per gene) and log-transformed RPKM values with an offset of 1, i.e., log_2_(RPKM + 1). We used inter-array distance to identify outlier subjects deviating more than 3 SDs from the mean (*14*). Outliers were identified relative to their age period in the fixed period analysis. Mitochondrial genes were removed. In addition, we performed an intersample comparison on the rank-normalized expression data of each window to further identify potential outlier samples. Visual inspection of scale invariance index (*R*^2^ of the correlation between raw and log-transformed connectivity values) and β values across windows revealed that specific samples caused disproportionate effects on these parameters, hence influencing power and connectivity. Only the SCZ group had samples with such an impact, and therefore, we removed three SCZ samples to reach the desired median connectivity with the minimum number of excluded individuals.

We also found that specific windows deviated from the proximal windows in the WGCNA parameter that we assessed. Note that consecutive sliding windows have 95% of the subjects in common; hence, there is a considerable redundancy to accommodate a slight decrease in the number of windows. For each window of 40 subjects, scale invariance index and median connectivity were estimated for β values ranging from 1 to 25. We selected β for each network such that the scale invariance index was greater than 80, and the connectivity was matched to the connectivity value (1.123) found in the age study. We tolerated connectivity values between one-third and three times the target connectivity. Windows where no β value returned connectivity values within these boundaries were removed (nine windows in NC [CN: 1, DLPFC: 3, HP: 5] and one in SCZ [HP: 1]). Thus, we could match NC and SCZ connectivity with the same value used in the age study. Notice that there was no outlier sample removal in the NC group; therefore, the only difference between studies relates to the windowing procedure.

Moreover, we wanted to assess the association of SCZ with functional biology along sliding window networks. Therefore, we computed an across module association of cell type and gene ontology with MAGMA enrichment for each network. The mean rank gene set test-based geneSetTest (alternative = “up”, type = “t”, ranks.only = T) function from the limma package was used to derive *P* values for cell type and MAGMA enrichment for all modules in all sliding window networks. For each sliding window network, the rlm function was used to predict MAGMA enrichment [−log_10_(*p_MAGMAenr_module*)] from cell type enrichment [−log_10_(*p_MAGMAenr_module*)], accounting for module size (*n_genes_module*) using robust linear modeling. See below

$$\mathrm{rlm}[-\log_{10}(p\_\mathrm{MAGMAenr}\_\mathrm{module})\;∼\;-\log_{10}(p\_\mathrm{CellTypeEnr}\_\mathrm{module})+n\_\mathrm{genes}\_\mathrm{module}]$$

For GO term enrichment, the ratios of genes found in specific GO term lists were computed for all modules in all networks. As with cell type enrichment, this ratio (ratio_GOterm_module) was used to predict MAGMA enrichment using rlm, accounting for module size. See below

$$\mathrm{rlm}[-\log_{10}(p\_\mathrm{MAGMAenr}\_\mathrm{module})\;∼\mathrm{ratio}\_\mathrm{GOterm}\_\mathrm{module}+\;n\_\mathrm{genes}\_\mathrm{module}]$$

The *Z*.stats of cell type or GO term enrichment for predicting MAGMA enrichment was extracted with their respective robust linear models and are depicted on the *y* axis of Fig. 8 (C and D).

#### Consensus

For each DLPFC or published network featuring SCZ risk modules according to the criteria that we defined, as shown in Figs. 2 and 5, we pooled SCZ risk genes from all SCZ risk modules. As our SCZ risk modules were identified using protein-coding gene lists and the report by Li *et al.* (*31*) had no module significantly enriched, we also considered for this analysis modules enriched for all biotype genes. For the negative lists, which were generated at 200 kbp from the negative SNP list, several networks had no significant enrichment; thus, we used the most significant module to generate the consensus, although it was not significant. Note that this procedure makes the test conservative, as it goes to the “advantage” of the negative list. We then intersected lists across networks to count how many times each gene appeared in an enriched module (minimum three modules). To compute the fraction of overlapping networks over the total for each gene, we divided the number of overlapping modules for each gene by the number of networks in which that gene was included: If a gene had not been annotated in a particular network, then that network did not count toward reaching this ratio. We only considered genes annotated in at least three networks (six for the postnatal consensus).

To obtain a *P* value for each consensus gene identified, we randomly permuted the network-specific SCZ risk modules and repeated the aforementioned procedure. The universe from which random genes were pooled consisted of genes expressed in the specific DLPFC or published networks. Last, we obtained a list of random consensus genes and defined the empirical *P* value as the number of occurrences in which the real consensus gene appeared in the random consensus list divided by the number of permutations (10,000). We set the significance threshold at an empirical *P* value < 0.05. We repeated this procedure for peri- and postnatal consensus lists separately.

Gene *pLI* scores were downloaded from the ExAC consortium release (*99*). A gene is generally considered likely to be loss-of-function intolerant if its *pLI* score is 0.9 or higher.

### Regulomic analysis

For the regulomic analysis, we used the gProfileR package to search for transcription factors and miRNA whose potential targets were enriched in the postnatal consensus list. We restricted the universe of transcription factors to those expressed above the minimum threshold in at least six of the networks considered. We corrected results for multiple comparisons using false discovery rate.

### iPSC data

The iPSC generation pipeline has been described in detail elsewhere (*33*). Ninety-five human neuronal samples with 56 to 70 days in vitro belonging to 26 male participants of European ancestry were available. The original report included 15 NC and 13 patients with SCZ. Of these subjects, RNA-seq data were available for 14 NC and 12 patients with SCZ. NC had a relatively low polygenic risk score for SCZ (bottom 37% of the European ancestry male distribution), and patients with SCZ had relatively high polygenic risk score (top 45% of the European ancestry male distribution). Using the criteria described above for gene filtering and outlier detection, we removed one outlier sample (inter-array correlation for this sample exceeded 3 SD; hence, we processed 94 samples). Next, keeping only the human genes, we gene-wise averaged all samples corresponding to the same subject (logRPKM expression) to obtain a robust estimate of gene expression and remove within-dataset interdependency. Similarly, the variables rRNA rate ratio of all reads aligned to rRNA regions to total reads, total assigned gene (proportion of aligned reads that were assigned to genes during read counting), and mitochondrial rate for human genes (proportion of reads that aligned to chrM) were also averaged. We regressed out along with the first principal component (PC1) the effect of these confounders, whereas we protected the effect of diagnosis, which, in these iPSCs, simply reflects the difference in terms of genetic risk for SCZ between patients and controls. We identified a network using WGCNA with the settings described above [except that we used the minimum beta granting scale invariance (beta = 7) as per the standard WGCNA settings, as we did not have multiple networks to compare]. Then, for each module, we computed the hypergeometric overrepresentation of SCZ genes at various extension windows and 22 non-GWAS consensus genes (5 consensus genes of 28 that were already in the PGC3 list were excluded to avoid double dipping, whereas 1 gene was not expressed in iPSC) as target gene lists.

To test the hypothesis that the non-GWAS consensus genes are more highly coexpressed with SCZ risk genes than expected by chance, we took as connectivity measure the median of the gene-wise mean connectivity of consensus genes with all SCZ risk genes from the adjacency matrix of the iPSC network identified and compared it to a null distribution of randomly permuted non-SCZ and nonconsensus genes, after matching for gene set size, GC content, gene length, and average expression as previously described.

The universe from which random genes were pooled consisted of genes expressed in the iPSC network, excluding SCZ risk genes (the most extensive list that we generated at 500 kbp from GWAS-significant SNPs was considered for this analysis) and consensus genes. Last, we defined the empirical *P* value as the number of occurrences in which the connectivity measure of the random genes exceeded the one of the consensus genes with SCZ risk genes divided by the number of permutations (10,000). We set the significance threshold at a one-sided empirical *P* value <0.05. We repeated this procedure for the nine extension windows previously created.

## Supplementary Materials

### This PDF file includes:

Figs. S1 to S11 Table S1 to S5 References

## Notes

View/request a protocol for this paper from *Bio-protocol*.
