# Supplementary material for: Consensus molecular environment of schizophrenia risk genes in coexpression networks shifting across age and brain regions
Source: Sci Adv. 2023 Apr 14;9(15):eade2812. doi: 10.1126/sciadv.ade2812 (PMC10104472; doi:10.1126/sciadv.ade2812)
Supplement: Supplementary file 1 — Figs. S1 to S11 Table S1 to S5 References [file sciadv.ade2812_sm.pdf]

Science Advances
AAAS

Supplementary Materials for

Consensus molecular environment of schizophrenia risk genes in coexpression networks shifting across age and brain regions

Giulio Pergola *et al.*

Corresponding author: Daniel R. Weinberger, daniel.weinberger@libd.org;
Alessandro Bertolino, alessandro.bertolino@uniba.it; Giulio Pergola, giulio.pergola@uniba.it

Sci. Adv. **9**, eade2812 (2023)
DOI: 10.1126/sciadv.ade2812

This PDF file includes:

Figs. S1 to S11
Table S1 to S5
References

1. SI Notes

1.1 Parameter setting and regional co-expression

We found that when imposing a criterion of four significant (Bonferroni-corrected p-value < 0.05) windows, only eight modules across 13 published networks survived (Table S2). This low number matched the most stringent individual window between 0 and 500 kbp from GWAS-significant SNPs and obscured the variability evident in the enrichment of different genomic extension windows. Because such a stringent consensus would hinder detection sensitivity, we opted to prioritize modules significant in at least three windows. Only one Hartl et al (*11*) subnetwork (BRNCTX) was considered for the downstream analysis based on brain region similarity with the DLPFC we analyzed from the LIBD repository. A parameter search for the other 20 subnetworks (not used in the downstream analysis) is shown in Supplementary Figure S11.

Genes differentially expressed in humans relative to apes, as well as druggable and loss of function sensitive genes, were generally underrepresented in *grey* modules (Supplementary Fig. S2). *Grey* genes were generally less expressed than *non-grey* genes (Supplementary Fig. S3), although there was a large overlap between distributions, with several modules expressed to a lower extent than *grey*.

1.2 Dentate gyrus granule cell layer specific enrichment for schizophrenia risk genes

Based on the cell specificity results in Figure 5, we hypothesized that data obtained via laser capture microdissection (LCM) - overrepresenting neurons relative to bulk tissue data - would show greater schizophrenia (SCZ) enrichment compared to matched-age bulk tissue hippocampus networks. A subset of 73 adult neurotypical controls (NC) of the hippocampus tissue homogenate dataset (see Table S1) also had RNAseq data from a study on the dentate gyrus granule cell layer (DG)(*23*). We found that the DG-based *SCZ risk module* was significant (Bonferroni-corrected p-value < 0.05) in more bins than the hippocampus *SCZ risk module* and included more *SCZ risk genes* in absolute terms, although enrichment strength was similar (Supplementary Fig. S5). Using two preprocessing procedures for bulk hippocampus data (with/without quantitative Surrogate Variable Analysis [qSVA]) (*66*), we probed the role of preprocessing in these results – we found that qSVA is a superior approach to generating biologically faithful networks which closely resemble networks derived from more cell-specific tissue collection.

When <u>not</u> using qSVA, we found one *SCZ risk module* in the DG and <u>none</u> in the bulk hippocampus. Interestingly, when we preprocessed bulk hippocampus data <u>using</u> qSVA, we found the two *SCZ risk module turquoise* and *darkorange*. Module turquoise in hippocampus with qSVA preprocessing allowed to neatly reproduce the results obtained in the same subjects by means of LCM (Supplementary Fig. S2). This result after qSVA supports the likelihood that co-expression, particularly without qSVA, is confounded by mRNA degradation. Even with qSVA, the module identified in the bulk tissue hippocampus was smaller than the one in DG and only clustered up to 318 *SCZ risk genes* in the most comprehensive list we used. In contrast, the DG aggregated 45% more *SCZ risk genes* in *turquoise*. Both DG and bulk hippocampus qSVA modules showed cell specificity for DG cells, supporting that WGCNA modules may capture cell type information (11), but preprocessing also plays a role. As is evident from Supplementary Figure S2, bulk hippocampus tissue processed without qSVA shows poor cell specificity relative to qSVA processing. These findings suggest that both biological and preprocessing features contribute to the observed co-expression patterns.

When considering DG and qSVA-processed bulk hippocampus modules, we found the Jaccard Index (JI = intersection/union of the sets considered) between the SCZ-enriched modules to be relatively low (0.21); the 893 shared genes were a minority and were enriched for synaptic, plasticity, and associative learning

ontologies. The 1659 genes specific to DG *turquoise* were overrepresented with functions related to cell projection organization (102 genes, 1.6-fold enrichment, q_{FDR} = .0078) and neurogenesis (131 genes, 1.46-fold enrichment, q_{FDR} = .02). In summary, qSVA successfully identifies modules with risk and functional profiles reminiscent of those accessible via biological cell population enrichment; however, cell population enriched data provide a greater degree of *SCZ risk gene* convergence on co-expression patterns and an insight to the context dependent functions of these genes otherwise unavailable.

1.3 MAGMA linear models

The model derived from the entire cohort networks was significant ($F_{[188,17807]}$ = 7.34, p-value < 2.2e-16, Adjusted R^2: 0.062). This model was superior to a "null model" that only included genetic covariates of no interest (maximum likelihood estimation obtained via anova, p-value = 3.03e-14), suggesting an association of co-expression variables with MAGMA-derived gene importance for SCZ. Module assignments were significant in all networks with higher significance for dorsolateral prefrontal cortex (DLPFC) and Caudate nucleus (Caudate nucleus $F_{[49, 17807]}$ = 2.6, p-value = 1.03e-08; DLPFC $F_{[42, 17807]}$ = 2.4, p-value = 8.8e-07).

The age-parsed model included the outcome of 11 networks (three for the Caudate nucleus and four each for hippocampus and DLPFC). The association of network features with MAGMA scores was significant ($F_{[451, 17544]}$ = 4.03, p-value < 2.2e-16, Adjusted R^2: 0.07). This model was significantly superior to the "null model" that only included genetic covariates of no interest (p-value < 2.2e-16). Significant module assignments included all four DLPFC networks (**perinatal**: $F_{[17, 17544]}$ = 2.9, p-value = 5.4e-05; **juvenile**: $F_{[33, 17544]}$ = 3.4, p-value = 1.4e-10; **adult**: $F_{[38, 17544]}$ = 1.6, p-value = .013; **older adult**: $F_{[40, 17544]}$ = 1.5, p-value = .017), the **perinatal** and **juvenile** hippocampus network (**perinatal**: $F_{[27, 17544]}$= 2.1, p-value = .0005; **juvenile**: $F_{[41, 17544]}$= 1.6, p-value = .009), and the all three Caudate nucleus networks (**juvenile**: $F_{[29, 17544]}$ = 2.2, p-value = .0002; **adult**: $F_{[51, 17544]}$ = 1.8, p-value = .0024; **older adult**: $F_{[41, 17544]}$ = 1.7, p-value = .0014). Additionally, we found that **perinatal** hippocampus total gene connectivity was negatively associated with MAGMA ($F_{[1, 17544]}$ = 17.4, t_{17544} = -3.9, p-value = 2.9e-05). In summary, these analyses revealed that module membership and connectivity in networks were related to MAGMA scores for SCZ. The largest differences between modules across age period parsed networks were observed in the **perinatal** and **juvenile** DLPFC; compounding this result, SCZ genes scoring high in terms of MAGMA tended to be peripheral in the **perinatal** hippocampal network, which was not the case in DLPFC networks.

1.4 Convergence of schizophrenia risk genes – additional results

Like gene sets enriched for *SCZ risk genes*, also other gene sets associated with SCZ by virtue of proximity to differentially methylated CpG islands, overall GWAS association (MAGMA in DLPFC), and differentially expressed genes (in Caudate nucleus) were underrepresented in the *grey* modules of the age-parsed networks we identified. In contrast, a negative control gene set not associated with SCZ (see Methods '*Network characterization and association with schizophrenia risk'*) was seldom depleted in the *grey*, and generally not overrepresented in *SCZ risk modules*. The underrepresentation in *grey* may relate at least in part to gene expression levels in brain, *per se (*Supplementary Fig. S*3)*. No *SCZ risk module* across the age period parsed networks overrepresented TWAS genes, although they were depleted in the *grey* modules of DLPFC networks. MAGMA enrichments, based on gene location rather than on eQTLs, were significantly overrepresented in *SCZ risk modules*. A permutation test controlling for counting a risk locus multiple times returned consistent results for our *SCZ risk modules* in the DLPFC and few others (Supplementary Fig. S4). Notably, enrichments detected in hippocampal modules were not significant when controlling for multiple counts from the same genetic locus, whereas DLPFC networks were less affected by this factor.

1.5 Risk gene flow – additional results

In DLPFC, 254 *SCZ risk genes* transitioned from non-grey **perinatal** modules into **juvenile** *grey*, whereas 234 **perinatal** *grey* genes clustered into other modules in the **juvenile** period (JI of *SCZ risk genes* between *grey* modules: 0.44; JI of all genes: 0.49; JIs when using prenatal samples: SCZ: 0.45; all genes: 0.51). Similar JIs were found in the replication **perinatal** to **juvenile** transition (JI of *SCZ risk genes* between *grey* modules: 0.63; JI of all genes: 0.64). The majority of **juvenile** *grey* genes remained non-clustered in the **adult** and **older adult** time windows; further, only a minority of genes in the **adult** and **older adult** *grey* modules were not already in the **juvenile** *grey* (JI between *grey* modules: **juvenile** to **adult**, 0.69 for SCZ, 0.72 for all genes; **adult** to **older adult**, 0.71 for SCZ, 0.75 for all genes).

In hippocampus, we identified two relevant transitions, one from the **perinatal** to **juvenile** period, and a subtler one in the **adult** to **older adult** period. In the first transition, 252 *SCZ risk genes* expressed in various **perinatal** modules ended up into **juvenile** *grey*; in contrast, 197 **perinatal** *grey* genes clustered in other modules in the **juvenile** period (JI of *SCZ risk genes* between *grey* modules: 0.53; JI of all genes: 0.57; JIs when using prenatal samples: SCZ: 0.51; all genes: 0.56). There was more continuity between the **juvenile** and **adult** time windows, close to that observed in the DLPFC (JI SCZ: 0.66; JI of all genes: 0.70). In the second relevant transition, 190 of the 713 **older adult** *grey* genes were clustered in other modules in the **adult** period; in turn, 134 **adult** *grey* genes were clustered in **older adulthood** (JI SCZ: 0.61; JI of all genes: 0.69, note the larger JI drop for SCZ likely associated with the **adult** *turquoise* contribution to **older adult** *grey*. No SCZ enrichment was significant in the **older adult** hippocampus, when a large proportion of *SCZ risk genes* from the **adult** module *turquoise* ended up in the **older adult** *grey*.

In **perinatal** DLPFC, module *black* included 27 *SCZ risk genes*, of which 18 (67%) were also co-expressed in the **juvenile** *blue* module. There was a sizable and significant overlap overall between **perinatal** *black* and **juvenile** *blue* (94 genes; empirical p = 10^{-4}, 10,000 permutations). By contrast, considering genes which were not SCZ *risk genes*, though a minority of 76 out of 159 (48%) *black* genes were still co-expressed in *blue*, this further represented a significant overlap across age periods for these two *SCZ risk modules* (empirical p = 10^{-4}, 10,000 permutations). In prenatal DLPFC, module *red* included 35 SCZ risk genes, of which 17 (46%) were also co-expressed in the **juvenile** *blue* module; the overlap between prenatal *red* and **juvenile** *blue* was 82 genes. In prenatal DLPFC, only 65 out of 206 (32%) *red* genes were still co-expressed in **juvenile** *blue*. In the replication **perinatal** to **juvenile** transition, module **perinatal** *red* included 21 *SCZ risk genes*, of which 12 (57%) were also co-expressed in the replication **juvenile** *blue* module. Instead, only a minority of *SCZ risk genes* in replication **perinatal** *red* genes were still co-expressed in blue (36%). The **perinatal-juvenile** intersection was strongly enriched for gene ontologies related to the regulation of neurodevelopment (23 genes, 5.5-fold enrichment, $q_{FDR} = 3.4\times10^{-7}$) and also to vesicle cytoskeletal trafficking (6 genes, 20-fold enrichment, $q_{FDR} = .0017$).

In **perinatal** hippocampus, module *red* included 85 *SCZ risk genes*. Of these, 39 (46%) were also co-expressed in the larger *turquoise* module of the **juvenile** window (the percentage was 42% considering only non-SCZ risk genes). In prenatal hippocampus, module *green* included 60 *SCZ risk genes*, with 32 (53%) still co-expressed in **juvenile** turquoise (the percentage was 46% considering only non-SCZ risk genes). In turn, **juvenile** *turquoise* shared some *SCZ risk genes* with the *turquoise* module in the **adult** window (JI SCZ risk genes: 0.41; JI of all genes: 0.43). A second stream of genes co-expressed in the **perinatal** *red* module was found in the **adult** *brown* module, via the **juvenile** *green* module and other non-enriched modules. A consensus module in the *red-brown* stream (160 shared genes) was strongly enriched for regulation of neurodevelopment (37 genes, 5.2-fold enrichment, $q_{FDR} = 2.3\times10^{-12}$) and regulation of trans-synaptic signaling (21 genes, 6.7-fold enrichment, $q_{FDR} = 1.8\times10^{-8}$). These ontologies resembled those observed for the early **perinatal/juvenile** intersection in DLPFC (44 genes shared with the DLPFC intersection, JI = 0.21). Instead, the *red-turquoise-*

turquoise stream (203 genes) overrepresented autophagy (16 genes, 5.4-fold enrichment, q_{FDR} = .0014) and catabolic processes (38 genes, 2.2-fold enrichment, q_{FDR} = .016).

1.6 Consensus genes across age periods

Supplementary Table S4 reports a list of the *SCZ risk genes* most frequently co-expressed within the modules shown in Figure 5 across age periods (*SKI*, *MKL1*, *GBF1*, *AP3D1*, *MGRN1*, *LRP1*). All these genes are included in the **perinatal** *SCZ risk module* in the DLPFC and hippocampus. Interestingly, *SCZ risk genes* found in multiple enriched modules were typically co-expressed in the enriched module already during perinatal life in the DLPFC (*black*) and hippocampus (*red*), hence highlighting the neurodevelopmental dimension of SCZ-enriched co-expression. *MKL1* gene is also among the consensus genes, based on DLPFC networks including other published networks.

2. SI Methods

2.1 Cell population enrichment preprocessing

For this analysis, we did as previously described, except that we did not covary for neuronal proportion, as DG-GCL samples were enriched for neurons, and we did not use RIN as confounding variable, because it was not available for all DG-GCL samples. Additionally, here we marginalized rather than protect age in the preprocessing, as these samples all came from adult individuals. To reduce variability between subjects, we quantile-normalized log-transformed RPKM values – something that was not possible when including non-adult samples without compromising the estimation of developmental changes. We also added an alternative pipeline in which we removed qSVA latent variables (*66*). In qSVA, an mRNA degradation experiment serves to obtain degradation components that are marginalized from gene expression data along with explicit confounders. The experiment was performed with adult hippocampus tissue homogenate, hence we applied it to hippocampal bulk tissue. In the qSVA pipeline, we took the degradation count matrix as reported by Jaffe et al. (*23*) and subset it to exactly match the samples available both in the DG-GCL and hippocampus bulk tissue dataset. We derived the appropriate number of surrogate variables (three) using the *sva* R package on the quantile normalized assay. We then added the surrogate variables in the statistical model including the explicit confounding variables to regress out both explicit and surrogate variables.

2.2 Machine Learning Pipeline – MAGMA score prediction

The Machine Learning pipeline processes two datasets: the Full dataset and the Age-parsed dataset. The Full dataset consists of postmortem data for DLPFC, caudate nucleus, and hippocampus, with no age distinction, whereas the Age-parsed dataset organizes the same information as the former in 11 cohorts, considering the three brain regions and four age stages described previously. Both datasets have 39 common features, including intrinsic gene attributes like 'start', 'width', 'NumTx', 'GC content', 'NumEx', 'pLI', 'strand', and 'gene type'. The categorical variables 'strand' (two levels) and 'gene type' (33 levels) were inserted in the model with one-hot encoding. The Full and Age-parsed datasets have instead different expression and co-expression features, including median gene expression levels (three in Full, 11 in Age-parsed) and kTotal and KME connectivity variables for co-expression network modules (three kTotal + 146 KME in Full, 11 kTotal + 409 KME in Age-parsed).

The Full dataset has dimensions of 21751 genes × 191 features, while the Age-parsed dataset has dimensions of 21751 genes × 470 features. Both datasets were randomly split into five groups using a five-fold Stratified Cross Validation algorithm, with the stratification based on the chromosome ('chr') to which each gene belongs. This process was repeated 200 times using a reproducible random seed. For each of these divisions, a five-fold Cross Validation pipeline was implemented, with one group used as the test set in each iteration. In each iteration, the training set (80% of genes) underwent feature selection using the Boruta algorithm (100), which compares the influence of a feature to its "shadow" counterpart obtained by shuffling its values, using a Random Forest prediction algorithm. The selected features are then used to train an XGBoost regressor and evaluate its performance using R^2. XGBoost is an ensemble of decision trees that are trained using an iterative gradient boosting method, with the number of parallel trees set to 100, the maximum depth of each tree set to 2, and the number of runs set to 100, using the squared error as the default objective function.

The described pipeline was repeated 200 times to provide a distribution of 1000 values of R^2 and the features selected by Boruta in the majority of runs for both datasets. The only variable of the kTotal type selected in over 50% of the iterations pertained to the DLPFC network of the Full dataset. It is worth pointing out that the Boruta algorithm, despite being applied to two datasets with very different dimensions, selected on average

comparable amounts of features. The distributions of selected feature numbers in overall 1000 runs are characterized as follows:

- $Median = 28$ (13.21% selection rate) and $IQR = 2.25$ for the Full dataset,
- $Median = 27$ (5.50% selection rate) and $IQR = 6$ for the Age-parsed dataset.

3. SI Figures

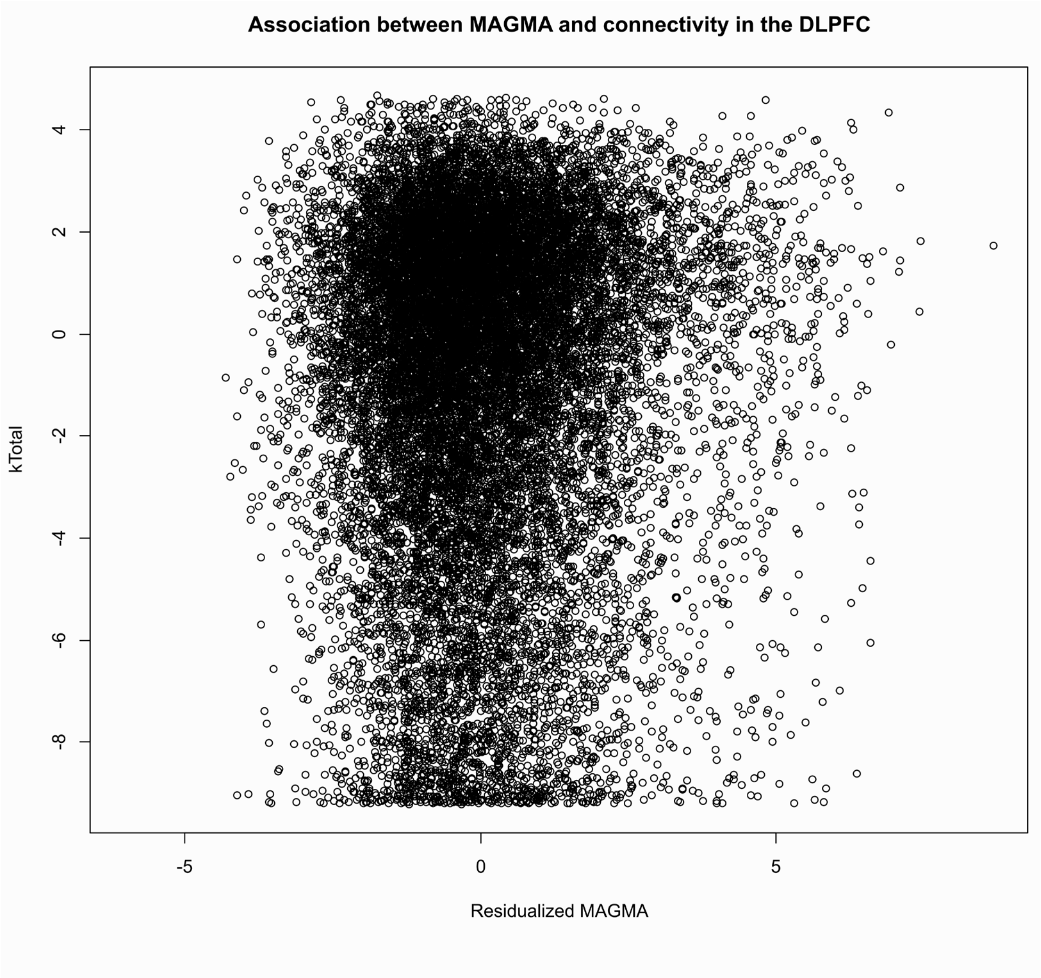

***Supplementary Fig. S1*. *Association between MAGMA and gene network connectivity in the dorsolateral prefrontal cortex*.** *The scatterplot shows a very weak relationship between the two variables. Plots obtained from kTotal of other networks do not fundamentally differ from the one above in this respect.*

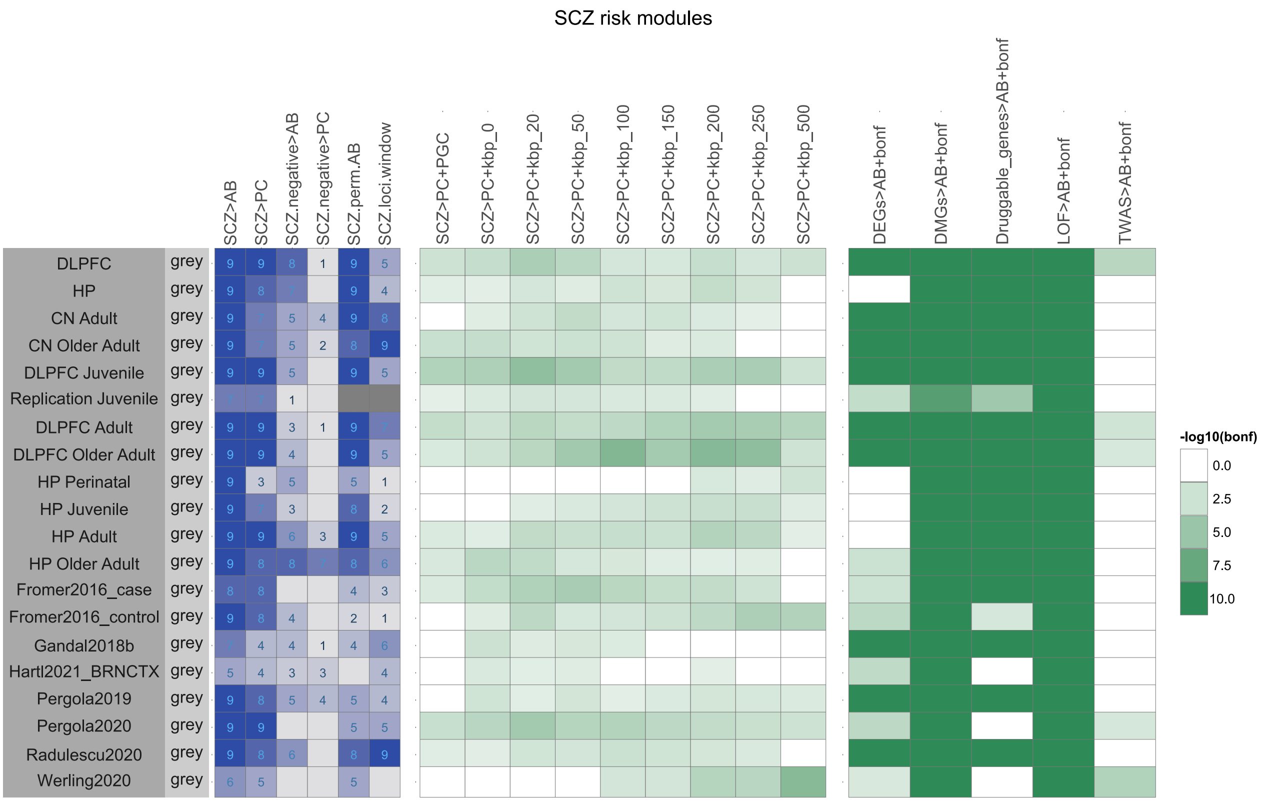

Supplementary Fig. S2. Schizophrenia risk depletion in grey modules. *The grey module was tested for depletion (underrepresentation instead of overrepresentation) of SCZ and other gene sets using the hypergeometric test. Columns with blue color scale report the number of extension windows in which the grey module was significantly depleted (Bonferroni-corrected p-value <0.05). Only networks with grey modules significantly depleted in at least 3 windows are shown. Abbreviations: CN: caudate nucleus; DLPFC: dorsolateral prefrontal cortex bulk tissue data; HP: hippocampus bulk tissue data; AB: all biotypes; PC: protein coding*

SCZ>AB: SCZ gene sets considering all biotypes.

SCZ>PC: SCZ gene sets considering only protein coding genes.

SCZ.negative>AB: negative gene lists not associated with SCZ considering all biotypes.

SCZ.negative>PC: negative gene lists not associated with SCZ considering only protein coding genes.

SCZ.perm>AB: enrichment results for all biotypes assessed via permutation testing (10,000 iterations).

SCZ.loci.window: genes from the same locus within each extension window (all biotypes) were collapsed into a unique hit per module.

DEGS>AB: differentially expressed genes, considering all biotypes

DMGS>AB: differentially methylated genes, considering all biotypes

Druggable_genes>AB: SCZ drug target genes, considering all biotypes.

LOF>AB: loss of function genes, considering all biotypes

TWAS>AB: transcription wide association study, considering all biotypes

A

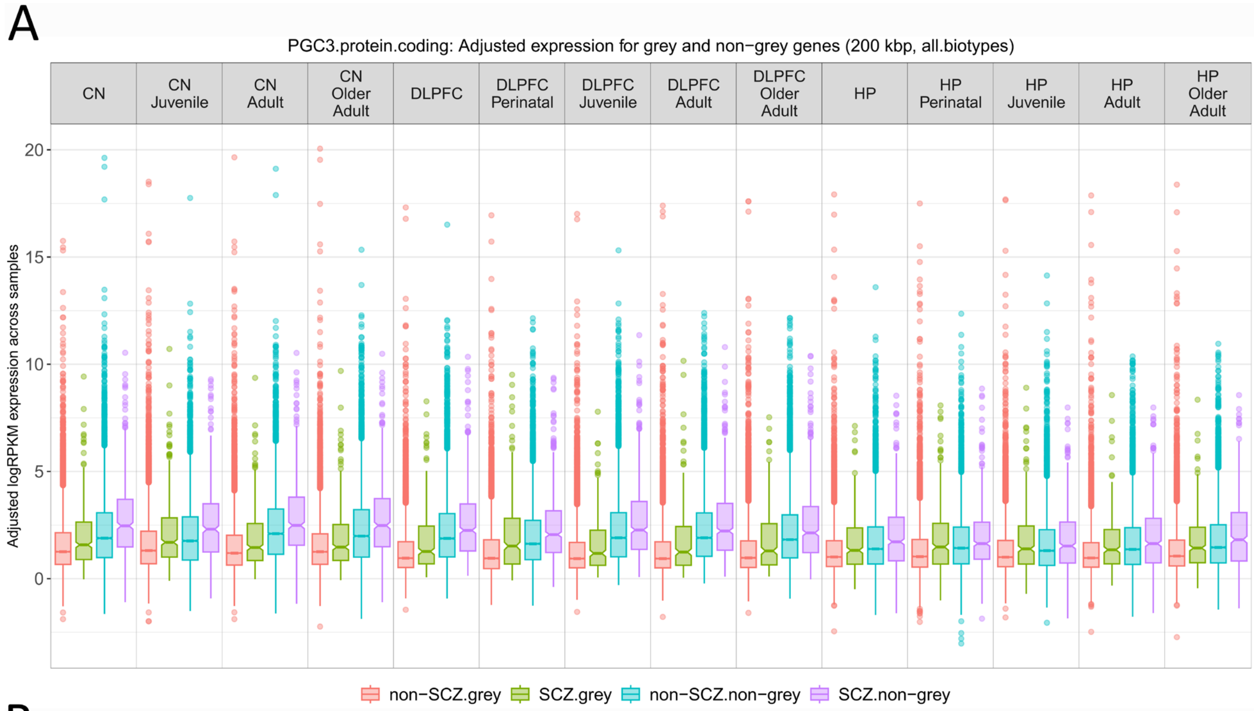

B

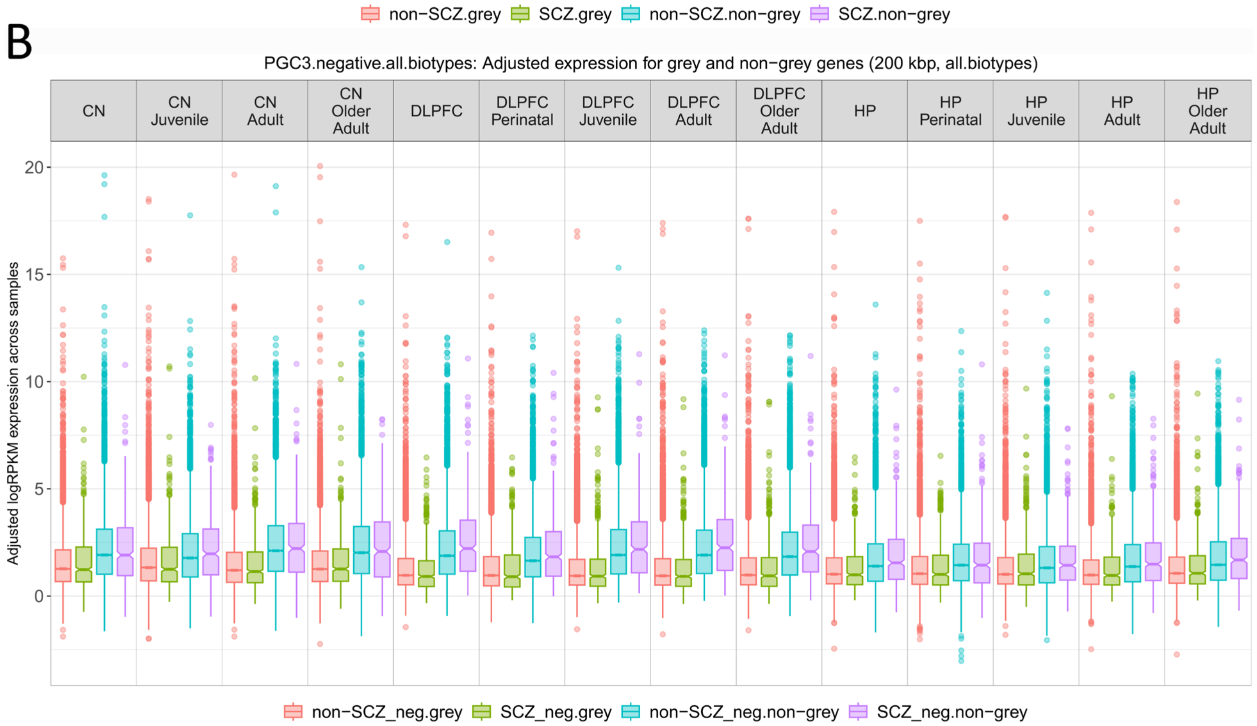

Supplementary Fig. S3. Gene expression of grey and non-grey genes in the schizophrenia and negative lists. *Median gene expression adjusted for confounders for A) Grey and non-grey network genes intersected with SCZ top loci genes (at 200kbp extension). A three-way ANOVA (for tissue, grey/non-grey, diagnosis) indicates that grey genes are more weakly expressed, accounting for most of the variance explained (grey/non-grey effect, eta*^{2} *= 0.0435), gene expression varies moderately between tissues (tissue effect, eta*^{2} *= 0.0094), while SCZ genes tend to be more highly expressed but the effect is relatively weak (diagnosis effect, eta*^{2} *= 0.0012). B) Grey and non-grey network genes intersected with SCZ-negative list (at 200kbp extension). Abbreviations: CN: caudate nucleus; DG: dentate gyrus granule cell layer data; DLPFC: dorsolateral prefrontal cortex; HP: hippocampus bulk tissue data; QSVA, quantitative surrogate variable analysis.*

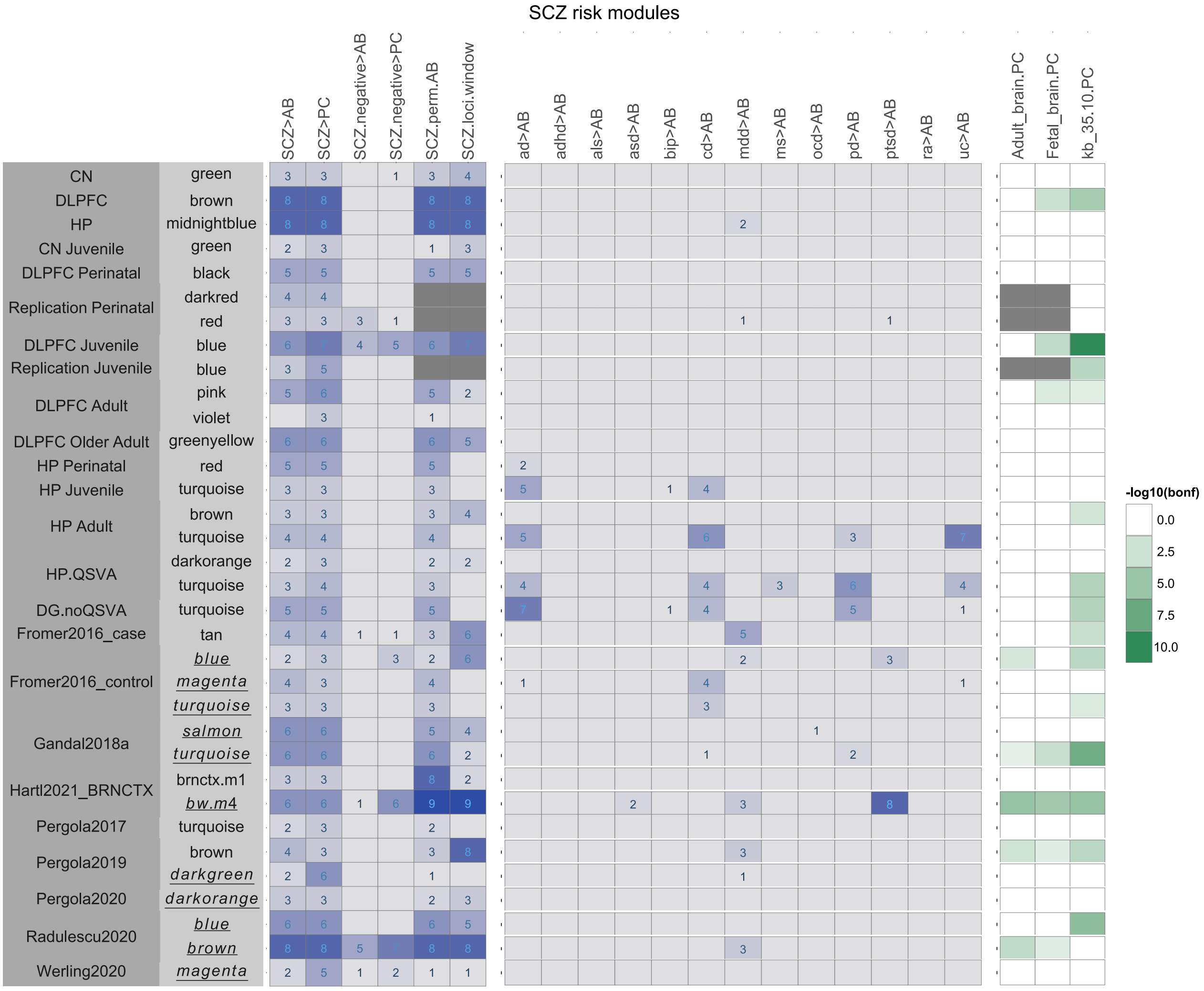

***Supplementary Fig. S4. Pathology specificity of the enrichment findings**. Figure shows enrichments for the SCZ risk modules (identified in Figure 2, Figure 5, and Supplementary Figure S5). Columns with blue color scale report the number of extension windows in which the hypergeometric test was significant. Alzheimer's was enriched at all age periods considered in the hippocampal SCZ risk modules, potentially suggesting co-expression pathways of shared risk. Abbreviations: CN: caudate nucleus; DLPFC: dorsolateral prefrontal cortex bulk tissue data; HP: hippocampus bulk tissue data; QSVA, quantitative surrogate variable analysis; AB: all biotypes; PC: protein coding; ad: Alzheimer disease; adhd: attention deficit hyperactivity disorder; als: amyotrophic lateral sclerosis; asd: autism spectrum disorder; bip: Bipolar disorder; cd: Crohn's disease; mdd: Major depressive disorder; ms: Multiple Sclerosis; ocd: Obsessive-compulsive disorder; pd: Parkinson's disease; ptsd: Post-traumatic stress disorder; ra: Rheumatoid arthritis; uc: Ulcerative colitis.*

SCZ>AB: SCZ gene sets considering all biotypes.

SCZ>PC: SCZ gene sets considering only protein coding genes.

SCZ.negative>AB: negative gene lists not associated with SCZ considering all biotypes.

SCZ.negative>PC: negative gene lists not associated with SCZ considering only protein coding genes.

SCZ.perm>AB: enrichment results for all biotypes assessed via permutation testing (10,000 iterations).

SCZ.loci.window: genes from the same locus within each extension window (all biotypes) were collapsed into a unique hit per module.

MAGMA enrichments (kb_35.10.PC): these tests consider all genetic variants and not just GWAS significant loci, mapping at 35kbp upstream and 10kbp downstream of "protein coding" module genes.
Adult_brain.PC: this test integrates MAGMA enrichment with chromatin accessibility data extracted from adult individuals to derive H-MAGMA competitive enrichment (protein coding).
Fetal_brain.PC: this test integrates MAGMA enrichment with chromatin accessibility data extracted from fetal individuals to derive H-MAGMA competitive enrichment (protein coding).

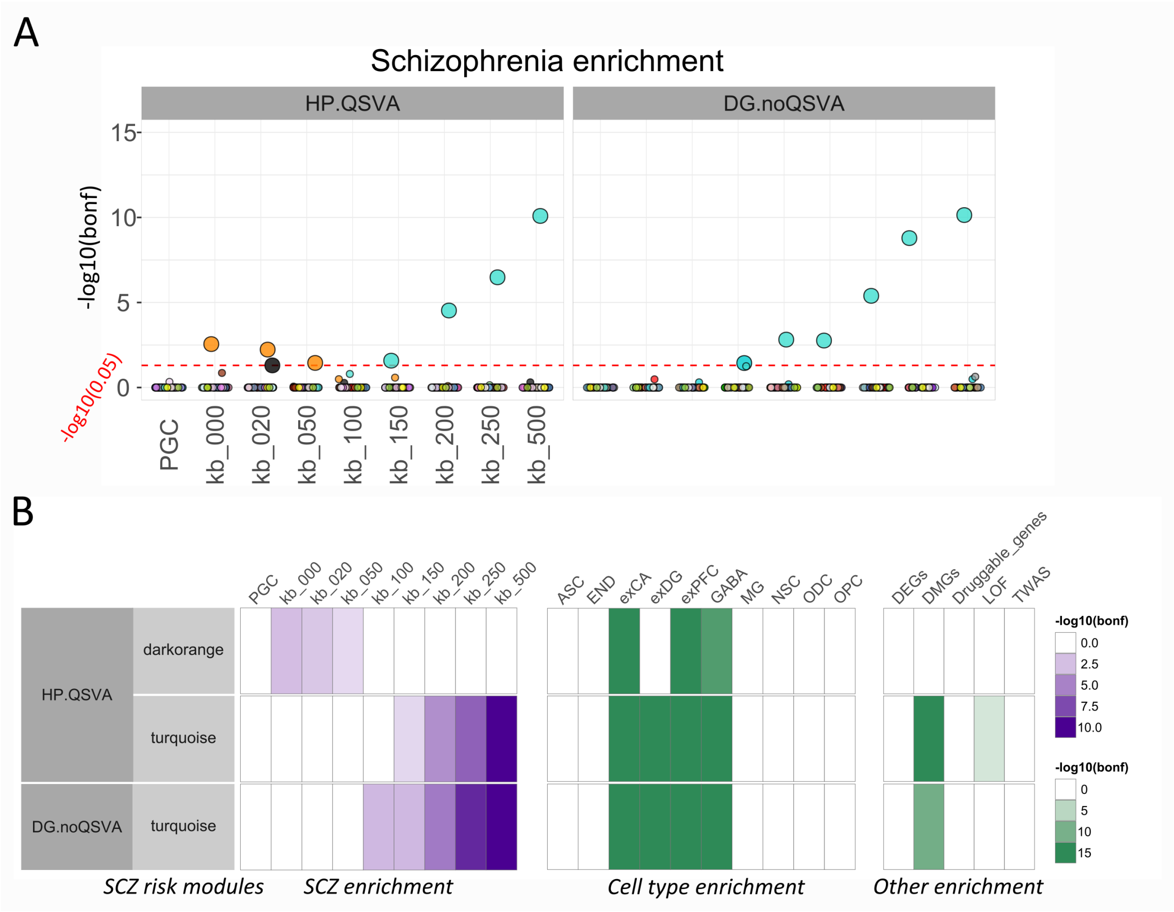

***Supplementary Fig. S5. Schizophrenia risk convergence and module characterization in dentate gyrus laser capture microdissection versus bulk hippocampus-derived networks**. A) Schizophrenia risk genes enrichment in hippocampus homogenate and dentate granule cell layer. B) Risk modules functional characterization. See Figure 2 captions in the main text for the abbreviations used here.*

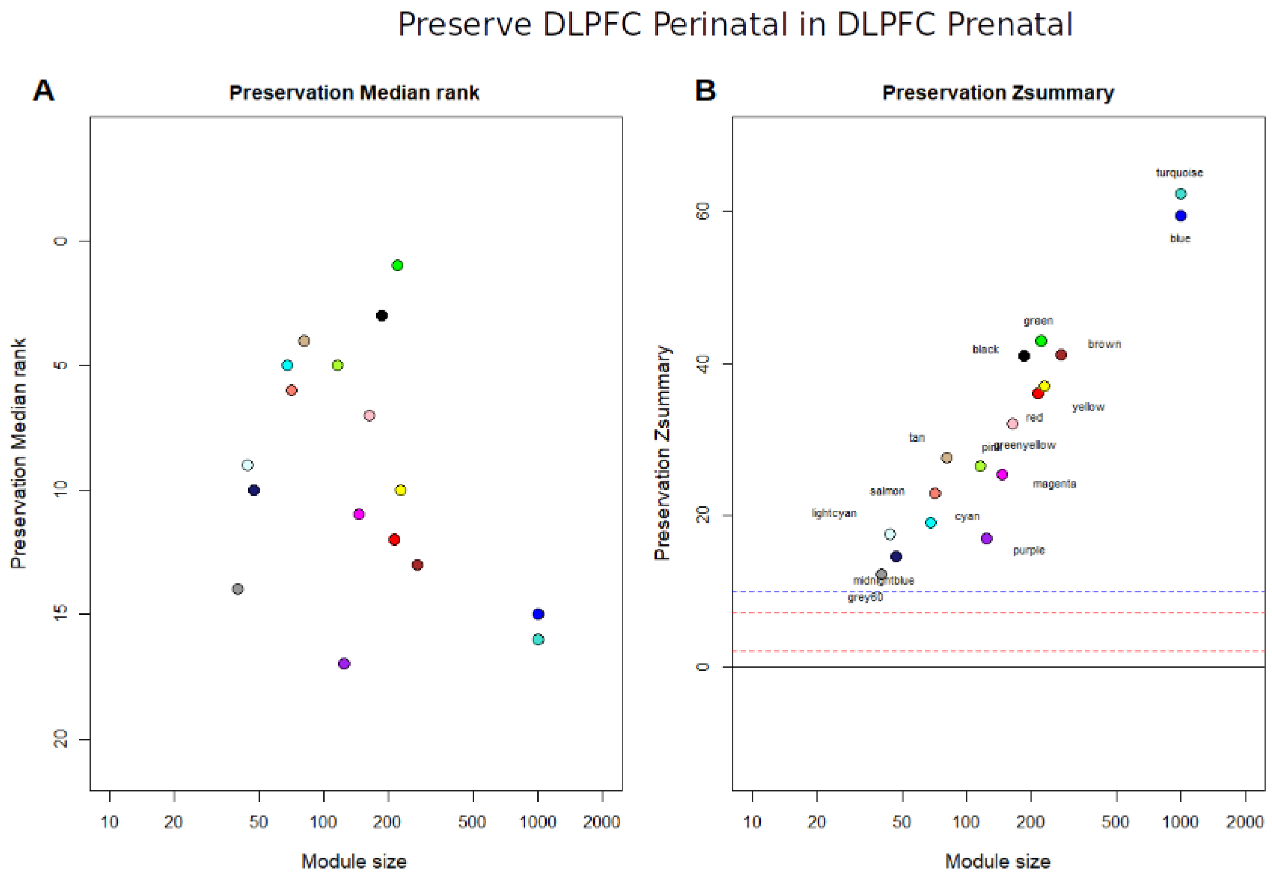

Supplementary Fig. S6. Dorsolateral prefrontal cortex perinatal module preservation in prenatal network. A) *Median preservation rank for the* ***perinatal*** *modules.* ***Perinatal*** *black risk module is ranked among top 5 in the prenatal network.* ***B)*** *Z-summary statistics for the preservation of* ***perinatal*** *modules in the prenatal network. Almost all modules are highly preserved (z-scores > 10). Dashed lines represent Z-score (blue: 10, red: 7, orange: 2).*

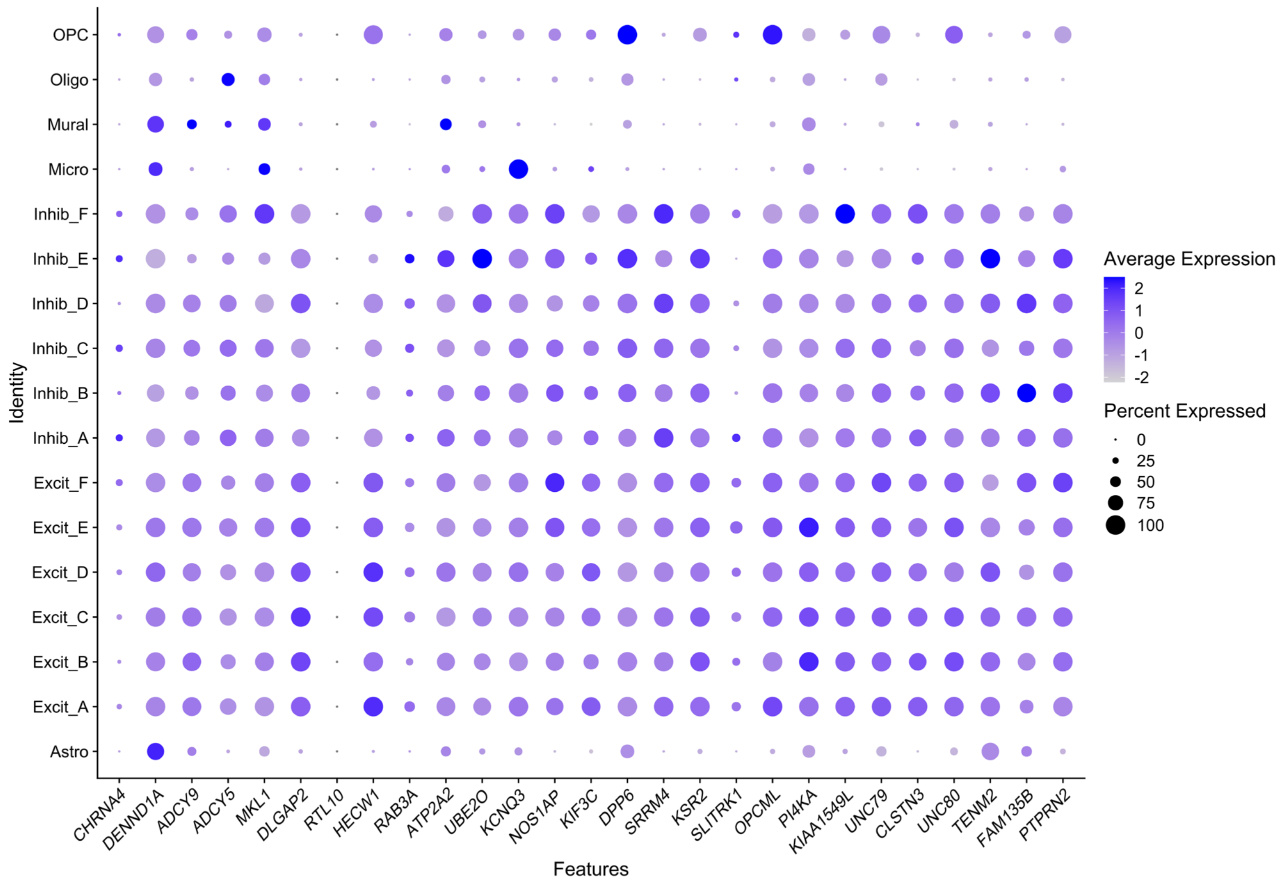

Supplementary Fig. S7. Expression levels of consensus genes in single nuclei cell type data*. The size of a dot encodes the percentage of nuclei expressing the consensus gene within a cluster, while the color encodes the average expression level across all cells within a cluster.*

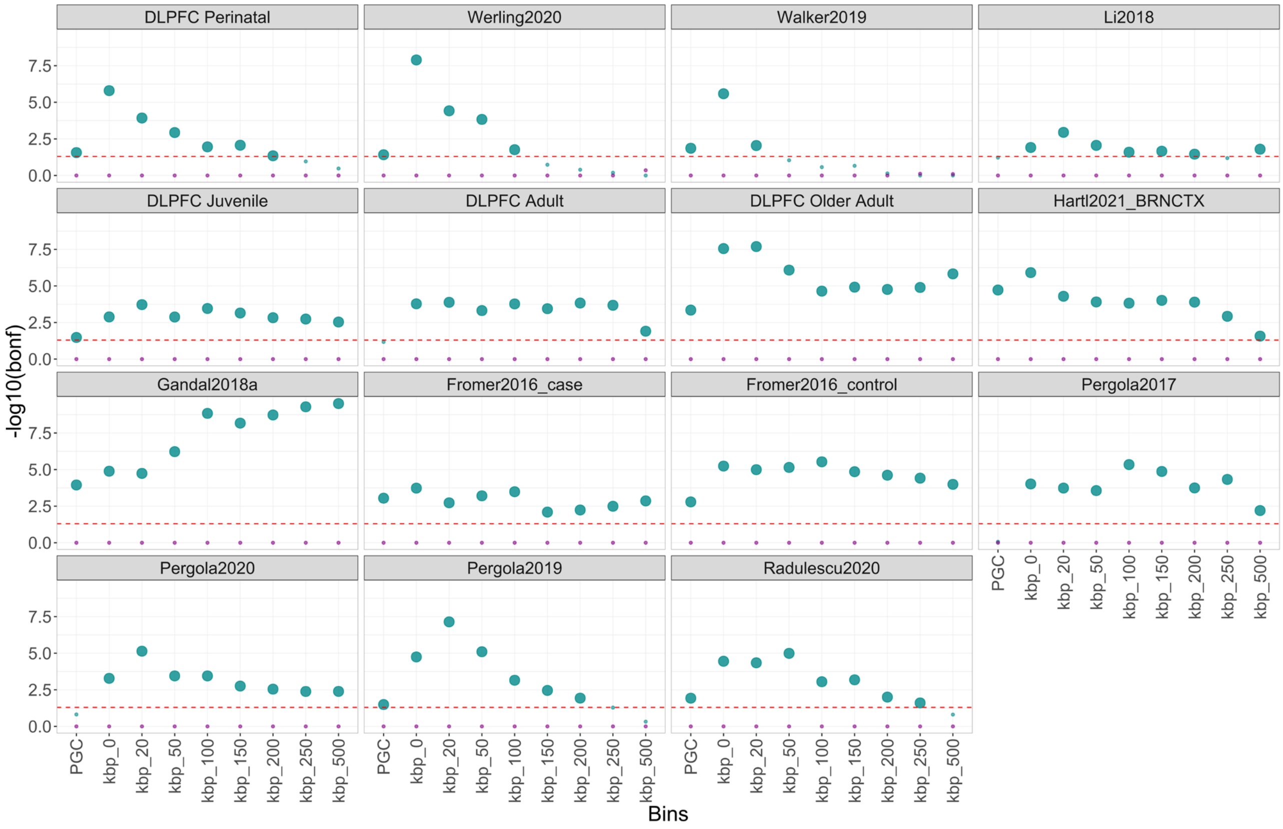

Supplementary Fig. S8*. *Schizophrenia risk gene enrichment in consensus gene module partners: *For each network the union of all module partners of consensus genes was taken (even if that module was not SCZ enriched). SCZ risk was assessed in the resulting gene set of consensus partners in twelve previously published networks. See Figure 2 captions in the main text for details.*

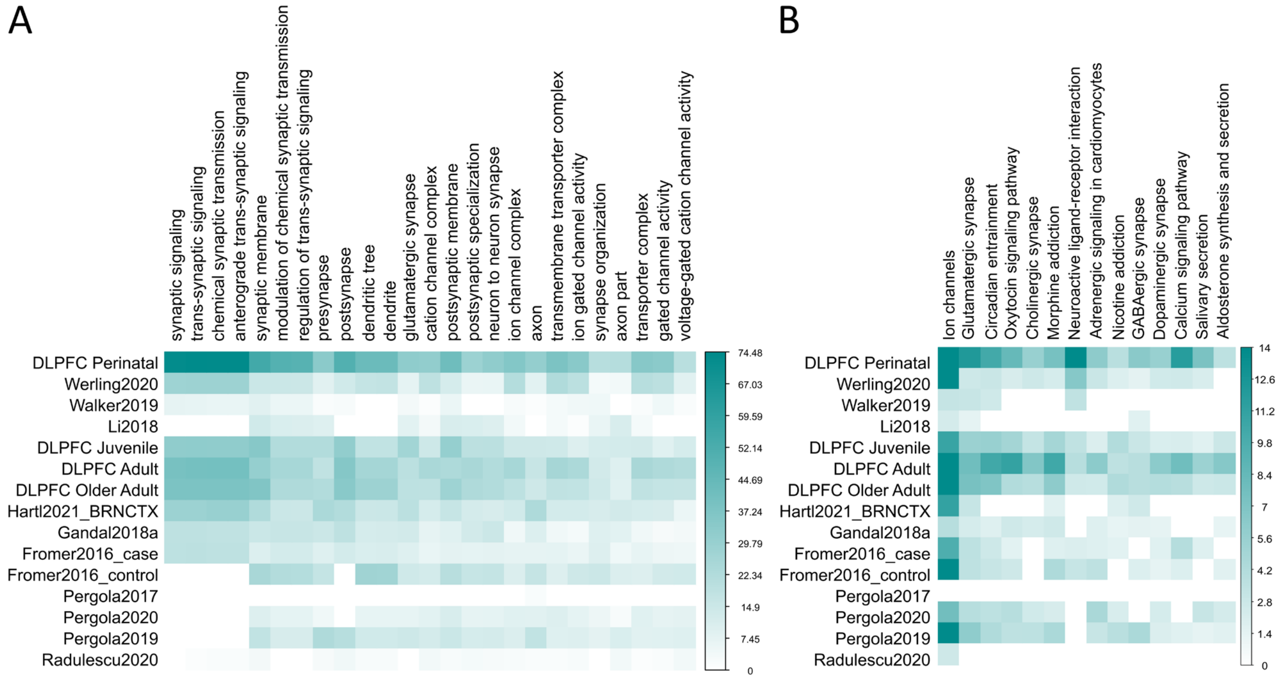

Supplementary Fig. S9*. *Gene ontology enrichment in consensus gene module partners: *For each network the union of all module partners of consensus genes was taken (even if that module was not SCZ enriched). Gene ontology enrichment was assessed using hypergeometric testing with A) GO and B) KEGG.*

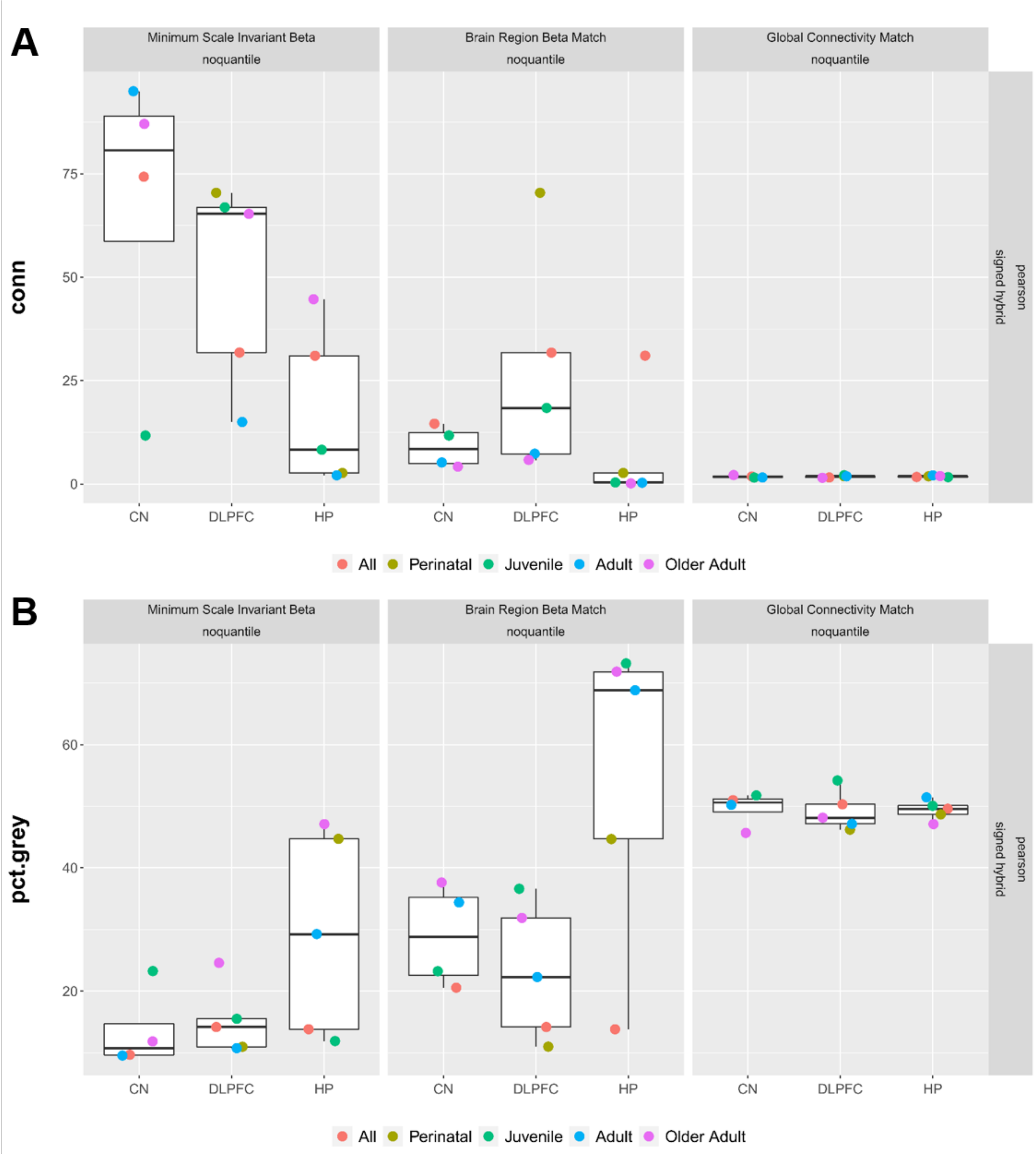

Supplementary Fig. S10. Network comparability. *Between-network variation of (A) median connectivity and (B) percentage of non-clustered genes across different criteria for network*

matching. Plots on the left (Minimum Scale Invariant Beta) used the minimum β to meet the scale invariance criterion for each network, as per the standard use of WGCNA. Middle plots used the minimum β across the networks of a single brain regions that met the scale invariance for all age periods. Plots on the right derive from a downward connectivity match between all networks in which β was selected to obtain comparable connectivity across all networks. Abbreviations: All, all age samples; CN, caudate nucleus; conn, median connectivity (sum of all edges) of network genes; DLPFC, dorsolateral prefrontal cortex; HP, hippocampus; pct.grey, percentage of genes assigned to the grey module.

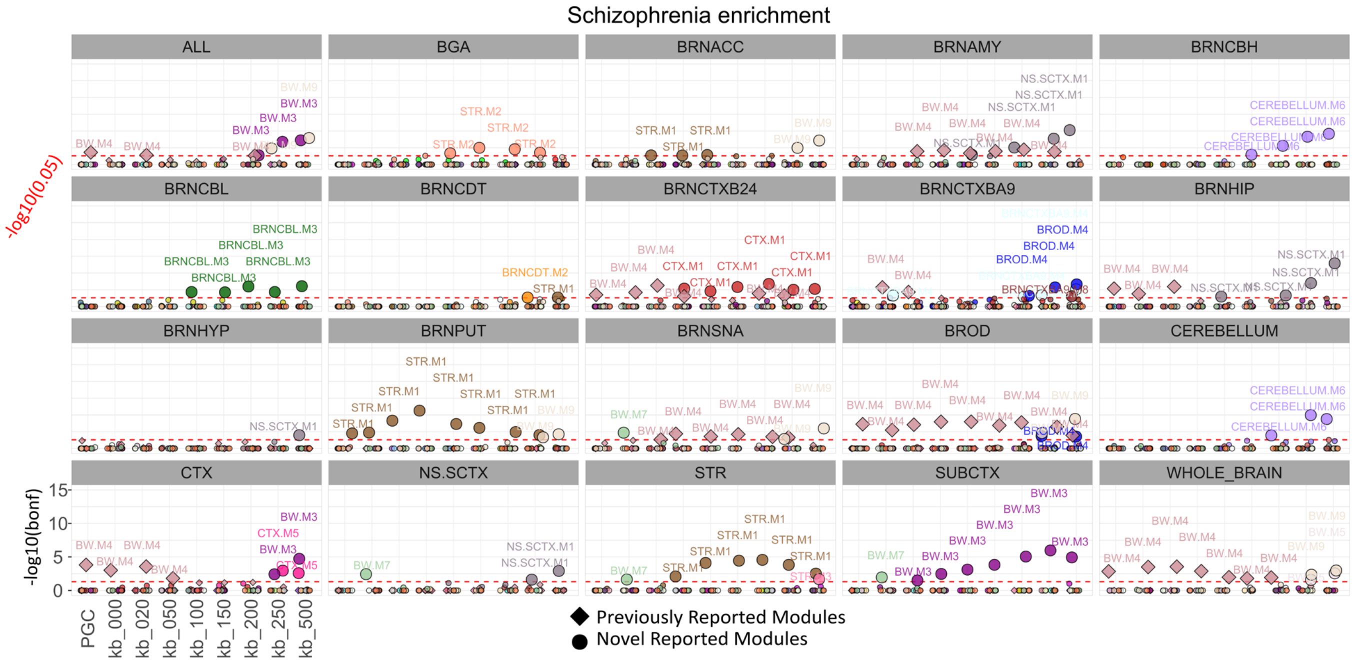

***Supplementary Fig. S11.* Schizophrenia risk convergence in sub-networks in Hartl2021.**
See Figure 2 caption for details on the tests computed and abbreviations.

4. SI Tables

Table S1. Demographics. *Abbreviations: AA: African American; EUR: European; CN: Caudate Nucleus bulk tissue data; DLPFC: dorsolateral prefrontal cortex bulk tissue data; HP: hippocampus bulk tissue data; DG: dentate gyrus granule cell layer data; NC: Neurotypical controls; SCZ: Patients with Schizophrenia.*

Study	Region/age period	NC sample size	SCZ sample size	Ancestry AA/EUR	Female (male) [ratio]	Age mean ± sd (years)	Age range (years)	Number of genes	Overall sample size
**Entire cohort**	CN	259	0	128/131	77 (182)<br>[0.42]	43.9 ± 20.2	0-90	20,888	374
	DLPFC	263	0	150/113	85 (178)<br>[0.48]	34.5 ± 22.3	-1-84	21,129	
	HP	278	0	152/126	87 (191)<br>[0.46]	35.8 ± 21.3	-1-84	20,421	
**Cell population enrichment**	HP/DG	73	0	38/35	19 (54)<br>[0.35]	47.9 ± 15.3	17-84	17,659	73
**Age stage parsed**	CN<br>*Juvenile*	49	0	26/23	17 (32)<br>[0.5]	12.1 ± 9.7	0-25	20,888	374
	CN<br>*Adult*	103	0	54/49	30 (73)<br>[0.41]	40.5 ± 7.3	25-50		
	CN<br>*Older Adult*	107	0	48/59	30 (77)<br>[0.39]	61.8 ± 10.1	50-90		
	DLPFC<br>Prenatal	26	0	22/4	15 (11)<br>[1.36]	-0.41 ± 0.0		20,421	
	HP<br>*Perinatal*	42	0	29/13	22 (20)<br>[1.1]	0.20 ± 1.2	-1-6		
	HP<br>*Juvenile*	49	0	26/23	11 (38)<br>[0.29]	18.2 ± 4.0	8-25		
	HP<br>*Adult*	104	0	57/47	32 (72)<br>[0.44]	40.0 ± 7.4	25-50		
	HP<br>*Older Adult*	83	0	40/43	22 (61)<br>[0.36]	58.9 ± 8.2	50-84		
	Replication DLPFC<br>*Perinatal*	50	0	24/22 (Asian n = 1, Hispanic n = 3)	19 (31)<br>[0.61]	0.17 ± 1.2	-0.6-4	14,553	85
	Replication DLPFC<br>*Juvenile*	35	0	7/12 (no ethnicity data for UCLA)	6 (29)<br>[0.20]	16.7 ± 4.0	8-24	14,553	
**Sliding windows**	CN	259	149	128/131 NC<br>81/68 SCZ	77 (182)<br>[0.42]<br>48 (101)<br>[0.48]	44.0 ± 20.3<br>51.7 ± 15.1	0-90<br>17-97	20,888<br>21,067	376 NC<br>186 SCZ
	DLPFC	263	134	150/113 NC<br>69/65 SCZ	85 (178)<br>[0.48]<br>41 (93)<br>[0.44]	34.6 ± 22.3<br>49.9 ± 16.0	-1-84<br>17-97	21,129<br>21,264	
	HP	278	112	152/126 NC<br>60/52 SCZ	87 (191)<br>[0.46]<br>37 (75)<br>[0.49]	35.8 ± 21.3<br>50.5 ± 15.2	-1-84<br>17-97	20,421<br>20,265	
	DG	85	70	43/42 NC<br>30/40 SCZ	43 (42)<br>[0.39]<br>22 (48)<br>[0.46]	48.1 ± 14.8<br>52.8 ± 15.7	17-84<br>17-84	18,963<br>19,111	

Table S2. Parameter setting. *Counts of significant (Bonferroni-corrected p-values < 0.05) modules for each network assessed for enrichment for protein-coding genes in the proximity of schizophrenia-significant SNPs in each of the nine reference lists considered individually [PGC: 500 kbp]. Three-lists column instead considers significance in at least 3 out of 9 lists for the counts. Four-lists column instead considers significance in at least 4 out of 9 lists for the counts.*

	PGC	0	20	50	100	150	200	250	500	3-lists	4-lists
**Fromer2016_case**	1	1	1	1	-	-	-	-	1	1	1
**Fromer2016_control**	-	1	1	1	-	-	2	2	3	3	-
**Gandal2018a**	1	1	1	2	2	2	1	2	1	2	2
**Gandal2018b**	-	1	1	-	-	-	-	-	-	-	-
**Gandal2018b_cs**	-	-	1	-	1	1	-	-	-	-	-
**Hartl2021_BRNCTX**	1	1	1	1	1	2	1	-	2	2	1
**Li2018**	1	-	-	-	-	-	-	-	-	-	-
**Pergola2017**	-	1	-	-	1	1	-	-	1	1	-
**Pergola2019**	1	1	1	2	1	1	1	1	1	2	1
**Pergola2020**	1	1	1	-	-	-	-	-	1	1	-
**Radulescu2020**	1	1	1	2	2	2	2	2	2	2	2
**Walker2019**	-	1	1	-	-	-	-	1	1	-	-
**Werling2020**	-	2	-	-	1	1	1	2	3	1	1
**Total**	**7**	**12**	**10**	**9**	**9**	**10**	**8**	**10**	**16**	**15**	**8**

Table S3. WGCNA parameters. *All networks were individually assessed to find the minimum exponent used to convert a correlation to an adjacency matrix (beta) that also achieved a scale-free topology network. The criterion to determine that the network was scale free was an R^2 > 0.8 in the correlation between the values in the matrix and their logarithmic transform. We noted the median connectivity for that beta [Connectivity at R^2 criterion]. Across all non-parsed and age-parsed networks, CN had the lowest median connectivity to give a scale free topology. Beta for other networks was set at a value causing their connectivity to match that of CN [Sft beta]. Abbreviations: CN: Caudate nucleus; DLPFC, dorsolateral prefrontal cortex; HP, hippocampus; Pct.grey, percentage of non-clustered grey genes.*

**Study**		**Tissue/age stage**	**Sft beta (used)**	**Sft beta at R^2 criterion (not used)**	**Connectivity at R^2 criterion (not used)**	**Matched connectivity (used)**	**Grey genes**	**Pct. grey**	**Number of modules**
**Age study**	***Entire cohort***	CN	9	9	1.123	1.123148	12349	59.12	50
		DLPFC	11	6	20.354	1.123148	12096	57.25	43
		HP	10	5	30.443	1.123148	11984	58.68	53
	Age stage parsed	CN *Juvenile*	13	6	35.429	1.123148	12442	59.57	30
		CN *Adult*	10	8	2.969	1.123148	12726	60.92	52
		CN *Older Adult*	9	4	52.438	1.123148	12550	60.08	42
		DLPFC Prenatal	19	15	3.867	1.123148	10957	51.86	23
		DLPFC *Perinatal*	16	3	597.623	1.123148	10505	49.72	18
		DLPFC *Juvenile*	12	5	62.116	1.123148	11651	55.14	34
		DLPFC *Adult*	10	6	13.305	1.123148	12125	57.39	39
		DLPFC *Older Adult*	9	4	61.867	1.123148	12123	57.38	41
		HP Prenatal	20	8	38.23	1.123148	12411	60.78	26
		HP *Perinatal*	14	6	51.874	1.123148	11672	57.16	28
		HP *Juvenile*	12	10	2.44	1.123148	12680	62.09	42
		HP *Adult*	11	7	10.527	1.123148	11509	56.36	41
		HP *Older Adult*	11	5	43.184	1.123148	11941	58.47	42
		Replication DLPFC *Perinatal*	BS = 29 polyA = 17	BS = 18 polyA = 10	BS = 7.546 polyA = 10.753	1.123148	11112	76.35	25
		Replication DLPFC *Juvenile*	UCLA = 25 polyA = 22	UCLA = 18 polyA = 12	UCLA = 4.079 polyA = 13.563	1.123148	10758	73.92	23
**Cell population enrichment**	***QSVA***	HP	6	5	14.3	14.1	4308	24.4	62
		DG	5	5	14.1	14.1	5376	30.4	58
	noQSVA	HP	5	5	28.5	25.4	2983	16.9	67
		DG	5	4	25.4	25.4	4343	24.6	42

Table S4. ***SCZ risk genes*** **most consistently co-expressed in enriched modules across age periods.** *Across the eight age-parsed networks considered, we identified six SCZ genes co-expressed within a SCZ risk module in at least six networks. For comparison, the maximum overlap when considering the negative gene list was three out of eight networks. Abbreviations: Chr, chromosome; DLPFC, dorsolateral prefrontal cortex; HP, hippocampus. Modules enriched for SCZ are in bold font.*

**EnsemblID**	**Symbol**	**Chr**	**Caudate juvenile**	**DLPFC perinatal**	**DLPFC juvenile**	**DLPFC adult**	**DLPFC older adult**	**HP perinatal**	**HP juvenile**	**HP adult**
**ENSG00000157933**	***SKI***	1	grey	**black**	**blue**	**pink**	**greenyellow**	**red**	grey	**turquoise**
**ENSG00000196588**	***MKL1***	22	grey	**black**	**blue**	**pink**	**greenyellow**	**red**	cyan	**brown**
**ENSG00000107862**	***GBF1***	10	grey	**black**	**blue**	**pink**	green	**red**	**turquoise**	**brown**
**ENSG00000065000**	***AP3D1***	19	grey	**black**	**blue**	blue	**greenyellow**	**red**	**turquoise**	**brown**
**ENSG00000102858**	***MGRN1***	16	grey	**black**	lightcyan	**pink**	**greenyellow**	**red**	**turquoise**	**turquoise**
**ENSG00000123384**	***LRP1***	12	grey	**black**	lightcyan	**pink**	**greenyellow**	**red**	**turquoise**	**turquoise**

5. Key Resource Table

DATASET USED	SOURCE	IDENTIFIER
Post-mortem brain tissue samples	Jaffe et al. (*6*)<br><br>Collado-Torres et al. (*61*)<br><br>Benjamin et al. (*52*)<br><br>Jaffe et al. (*23*)<br><br>Dataset: Allen Institute for Brain Science (2010). Allen Developing Human Brain Atlas: Developmental Transcriptome [dataset]. Available from brainspan.org. RRID:SCR_008083 | Primary publication: Miller et al. (*101*)<br><br>Gandal et al. (*36*)<br><br>Tran et al. (*41*)	**DLPFC and HIPPOCAMPUS:** http://eqtl.brainseq.org/phase2/<br><br>https://github.com/LieberInstitute/brainseq_phase2<br><br>**CAUDATE:** https://erwinpaquolalab.libd.org/caudate_eqtl/<br><br>https://github.com/LieberInstitute/BrainSeqPhase3Caudate<br><br>**DENTATE GYRUS:** https://github.com/LieberInstitute/dg_hippo_paper<br><br>**DLPFC Replication:** https://www.brainspan.org/static/download.html<br><br>https://www.synapse.org/#!Synapse:syn4587609<br><br>**DLPFC 10x snRNAseq:** https://github.com/LieberInstitute/10xPilot_snRNAseq-human
Degradation matrix	Jaffe et al. (*23*)	https://jaffe-nat-neuro-dggcl.s3.us-east-2.amazonaws.com/DataS1_DGGCL_eQTLs_plusHippo.csv.gz<br><br>https://research.libd.org/dg_hippo_paper/data.html

European Ancestry data	Collado-Torres et al. (*61*)	
Hartl2021	Hartl et al. (*11*)	Supplementary Table 1
Pergola2017	Pergola et al. (*13*)	https://www.ncbi.nlm.nih.gov/projects/gap/cgi-bin/study.cgi?study_id=phs000417.v1.p1<br><br>https://www.ncbi.nlm.nih.gov/geo/query/acc.cgi?acc=GSE30272
Pergola2019	Pergola et al. (*14*)	
Pergola2020	Pergola et al. (*35*)	Common Mind Consortium: https://www.synapse.org/#!Synapse:syn2759792/wiki/69613
Radulescu2020	Radulescu et al. (*12*)	
Gandal2018a	Gandal et al. (*10*)	
Gandal2018b<br>Gandal2018b_cs	Gandal et al. *(36)*	
Fromer2016_case<br>Fromer2016_control	Fromer et al. (*5*)	Common Mind Consortium: https://www.synapse.org/#!Synapse:syn2759792/wiki/69613
Werling2020	Werling et al. (*27*)	
Li2018	Li et al. (*31*)	
Walker2019	Walker et al. (*26*)	
iPSC dataset	Page et al. (3*3*)	https://stemcell.libd.org/schizophrenia/RNAseq/dataset001/
PGC3 reference list. Pathology reference list. Other enrichment reference lists(Cell Specificity, TWAS, DEGs, DMGs, LoF)	Papers cited in correspondence with data mentions.	

DATASET GENERATED	SOURCE	IDENTIFIER
Preprocessed datasets generated in this manuscript	Zenodo	https://doi.org/10.5281/zenodo.5676480
WGCNA network outputs for this manuscript	Zenodo	https://doi.org/10.5281/zenodo.5676480
Code generated	Github	https://github.com/LieberInstitute/Brain_WGCNA
For future analysis and research expansion		https://nets.libd.org/age_wgcna/
**SOFTWARE USED**	**SOURCE**	**IDENTIFIER**
ComplexHeatmap R package	Gu et al. (10*2*)	https://bioconductor.org/packages/release/bioc/html/ComplexHeatmap.html
WGCNA	Zhang et al. (*34*)<br>Langfelder et al. (*103*)	https://cran.r-project.org/web/packages/WGCNA/index.html
clusterProfiler R package	Yu et al. (*68*)	https://bioconductor.org/packages/release/bioc/html/clusterProfiler.html
MAGMA	de Leeuw et al. (*104*)	https://ctg.cncr.nl/software/magma
biomaRt R package	Durinck et al. (*105*)	https://bioconductor.org/packages/release/bioc/html/biomaRt.html
tidyverse R package		https://cran.r-project.org/web/packages/tidyverse/index.html
R (v4.X.X)	R Development Core (10*6*)	https://www.r-project.org/
ggplot2 R package		https://cran.r-project.org/web/packages/ggplot2/index.html

sankeyD3 R package		https://github.com/fbreitwieser/sankeyD3
gg4hx R package		https://teunbrand.github.io/ggh4x/

REFERENCES AND NOTES

1. I. I. Gottesman, J. Shields, A polygenic theory of schizophrenia. *Proc. Natl. Acad. Sci. U.S.A.* **58**, 199–205 (1967).

2. V. Trubetskoy, A. F. Pardinas, T. Qi, G. Panagiotaropoulou, S. Awasthi, T. B. Bigdeli, J. Bryois, C. Y. Chen, C. A. Dennison, L. S. Hall, M. Lam, K. Watanabe, O. Frei, T. Ge, J. C. Harwood, F. Koopmans, S. Magnusson, A. L. Richards, J. Sidorenko, Y. Wu, J. Zeng, J. Grove, M. Kim, Z. Li, G. Voloudakis, W. Zhang, M. Adams, I. Agartz, E. G. Atkinson, E. Agerbo, M. Al Eissa, M. Albus, M. Alexander, B. Z. Alizadeh, K. Alptekin, T. D. Als, F. Amin, V. Arolt, M. Arrojo, L. Athanasiu, M. H. Azevedo, S. A. Bacanu, N. J. Bass, M. Begemann, R. A. Belliveau, J. Bene, B. Benyamin, S. E. Bergen, G. Blasi, J. Bobes, S. Bonassi, A. Braun, R. A. Bressan, E. J. Bromet, R. Bruggeman, P. F. Buckley, R. L. Buckner, J. Bybjerg-Grauholm, W. Cahn, M. J. Cairns, M. E. Calkins, V. J. Carr, D. Castle, S. V. Catts, K. D. Chambert, R. C. K. Chan, B. Chaumette, W. Cheng, E. F. C. Cheung, S. A. Chong, D. Cohen, A. Consoli, Q. Cordeiro, J. Costas, C. Curtis, M. Davidson, K. L. Davis, L. de Haan, F. Degenhardt, L. E. DeLisi, D. Demontis, F. Dickerson, D. Dikeos, T. Dinan, S. Djurovic, J. Duan, G. Ducci, F. Dudbridge, J. G. Eriksson, L. Fananas, S. V. Faraone, A. Fiorentino, A. Forstner, J. Frank, N. B. Freimer, M. Fromer, A. Frustaci, A. Gadelha, G. Genovese, E. S. Gershon, M. Giannitelli, I. Giegling, P. Giusti-Rodriguez, S. Godard, J. I. Goldstein, J. Gonzalez Penas, A. Gonzalez-Pinto, S. Gopal, J. Gratten, M. F. Green, T. A. Greenwood, O. Guillin, S. Guloksuz, R. E. Gur, R. C. Gur, B. Gutierrez, E. Hahn, H. Hakonarson, V. Haroutunian, A. M. Hartmann, C. Harvey, C. Hayward, F. A. Henskens, S. Herms, P. Hoffmann, D. P. Howrigan, M. Ikeda, C. Iyegbe, I. Joa, A. Julia, A. K. Kahler, T. Kam-Thong, Y. Kamatani, S. Karachanak-Yankova, O. Kebir, M. C. Keller, B. J. Kelly, A. Khrunin, S. W. Kim, J. Klovins, N. Kondratiev, B. Konte, J. Kraft, M. Kubo, V. Kucinskas, Z. A. Kucinskiene, A. Kusumawardhani, H. Kuzelova-Ptackova, S. Landi, L. C. Lazzeroni, P. H. Lee, S. E. Legge, D. S. Lehrer, R. Lencer, B. Lerer, M. Li, J. Lieberman, G. A. Light, S. Limborska, C. M. Liu, J. Lonnqvist, C. M. Loughland, J. Lubinski, J. J. Luykx, A. Lynham, M. Macek, Jr., A. Mackinnon, P. K. E. Magnusson, B. S. Maher, W. Maier, D. Malaspina, J. Mallet, S. R. Marder, S. Marsal, A. R. Martin, L. Martorell, M. Mattheisen, R. W. McCarley, C. McDonald, J. J. McGrath, H. Medeiros, S. Meier, B. Melegh, I. Melle, R. I. Mesholam-

Gately, A. Metspalu, P. T. Michie, L. Milani, V. Milanova, M. Mitjans, E. Molden, E. Molina, M. D. Molto, V. Mondelli, C. Moreno, C. P. Morley, G. Muntane, K. C. Murphy, I. Myin-Germeys, I. Nenadic, G. Nestadt, L. Nikitina-Zake, C. Noto, K. H. Nuechterlein, N. L. O'Brien, F. A. O'Neill, S. Y. Oh, A. Olincy, V. K. Ota, C. Pantelis, G. N. Papadimitriou, M. Parellada, T. Paunio, R. Pellegrino, S. Periyasamy, D. O. Perkins, B. Pfuhlmann, O. Pietilainen, J. Pimm, D. Porteous, J. Powell, D. Quattrone, D. Quested, A. D. Radant, A. Rampino, M. H. Rapaport, A. Rautanen, A. Reichenberg, C. Roe, J. L. Roffman, J. Roth, M. Rothermundt, B. P. F. Rutten, S. Saker-Delye, V. Salomaa, J. Sanjuan, M. L. Santoro, A. Savitz, U. Schall, R. J. Scott, L. J. Seidman, S. I. Sharp, J. Shi, L. J. Siever, E. Sigurdsson, K. Sim, N. Skarabis, P. Slominsky, H. C. So, J. L. Sobell, E. Soderman, H. J. Stain, N. E. Steen, A. A. Steixner-Kumar, E. Stogmann, W. S. Stone, R. E. Straub, F. Streit, E. Strengman, T. S. Stroup, M. Subramaniam, C. A. Sugar, J. Suvisaari, D. M. Svrakic, N. R. Swerdlow, J. P. Szatkiewicz, T. M. T. Ta, A. Takahashi, C. Terao, F. Thibaut, D. Toncheva, P. A. Tooney, S. Torretta, S. Tosato, G. B. Tura, B. I. Turetsky, A. Ucok, A. Vaaler, T. van Amelsvoort, R. van Winkel, J. Veijola, J. Waddington, H. Walter, A. Waterreus, B. T. Webb, M. Weiser, N. M. Williams, S. H. Witt, B. K. Wormley, J. Q. Wu, Z. Xu, R. Yolken, C. C. Zai, W. Zhou, F. Zhu, F. Zimprich, E. C. Atbasoglu, M. Ayub, C. Benner, A. Bertolino, D. W. Black, N. J. Bray, G. Breen, N. G. Buccola, W. F. Byerley, W. J. Chen, C. R. Cloninger, B. Crespo-Facorro, G. Donohoe, R. Freedman, C. Galletly, M. J. Gandal, M. Gennarelli, D. M. Hougaard, H. G. Hwu, A. V. Jablensky, S. A. McCarroll, J. L. Moran, O. Mors, P. B. Mortensen, B. Muller-Myhsok, A. L. Neil, M. Nordentoft, M. T. Pato, T. L. Petryshen, M. Pirinen, A. E. Pulver, T. G. Schulze, J. M. Silverman, J. W. Smoller, E. A. Stahl, D. W. Tsuang, E. Vilella, S. H. Wang, S. Xu; Indonesia Schizophrenia Consortium; PsychENCODE; Psychosis Endophenotypes International Consortium; The SynGO Consortium, R. Adolfsson, C. Arango, B. T. Baune, S. I. Belangero, A. D. Borglum, D. Braff, E. Bramon, J. D. Buxbaum, D. Campion, J. A. Cervilla, S. Cichon, D. A. Collier, A. Corvin, D. Curtis, M. D. Forti, E. Domenici, H. Ehrenreich, V. Escott-Price, T. Esko, A. H. Fanous, A. Gareeva, M. Gawlik, P. V. Gejman, M. Gill, S. J. Glatt, V. Golimbet, K. S. Hong, C. M. Hultman, S. E. Hyman, N. Iwata, E. G. Jonsson, R. S. Kahn, J. L. Kennedy, E. Khusnutdinova, G. Kirov, J. A. Knowles, M. O. Krebs, C. Laurent-Levinson, J. Lee, T. Lencz, D. F. Levinson, Q. S. Li, J. Liu, A. K. Malhotra, D. Malhotra, A. McIntosh, A. McQuillin, P. R. Menezes, V. A. Morgan,

D. W. Morris, B. J. Mowry, R. M. Murray, V. Nimgaonkar, M. M. Nothen, R. A. Ophoff, S. A. Paciga, A. Palotie, C. N. Pato, S. Qin, M. Rietschel, B. P. Riley, M. Rivera, D. Rujescu, M. C. Saka, A. R. Sanders, S. G. Schwab, A. Serretti, P. C. Sham, Y. Shi, D. St Clair, H. Stefansson, K. Stefansson, M. T. Tsuang, J. van Os, M. P. Vawter, D. R. Weinberger, T. Werge, D. B. Wildenauer, X. Yu, W. Yue, P. A. Holmans, A. J. Pocklington, P. Roussos, E. Vassos, M. Verhage, P. M. Visscher, J. Yang, D. Posthuma, O. A. Andreassen, K. S. Kendler, M. J. Owen, N. R. Wray, M. J. Daly, H. Huang, B. M. Neale, P. F. Sullivan, S. Ripke, J. T. R. Walters, M. C. O'Donovan; Schizophrenia Working Group of the Psychiatric Genomics Consortium, Mapping genomic loci implicates genes and synaptic biology in schizophrenia. *Nature* **604**, 502–508 (2022).

3. E. A. Boyle, Y. I. Li, J. K. Pritchard, An expanded view of complex traits: From polygenic to omnigenic. *Cell* **169**, 1177–1186 (2017).

4. M. Li, A. E. Jaffe, R. E. Straub, R. Tao, J. H. Shin, Y. Wang, Q. Chen, C. Li, Y. Jia, K. Ohi, B. J. Maher, N. J. Brandon, A. Cross, J. G. Chenoweth, D. J. Hoeppner, H. Wei, T. M. Hyde, R. McKay, J. E. Kleinman, D. R. Weinberger, A human-specific AS3MT isoform and BORCS7 are molecular risk factors in the 10q24.32 schizophrenia-associated locus. *Nat. Med.* **22**, 649–656 (2016).

5. M. Fromer, P. Roussos, S. K. Sieberts, J. S. Johnson, D. H. Kavanagh, T. M. Perumal, D. M. Ruderfer, E. C. Oh, A. Topol, H. R. Shah, L. L. Klei, R. Kramer, D. Pinto, Z. H. Gumus, A. E. Cicek, K. K. Dang, A. Browne, C. Lu, L. Xie, B. Readhead, E. A. Stahl, J. Xiao, M. Parvizi, T. Hamamsy, J. F. Fullard, Y. C. Wang, M. C. Mahajan, J. M. Derry, J. T. Dudley, S. E. Hemby, B. A. Logsdon, K. Talbot, T. Raj, D. A. Bennett, P. L. De Jager, J. Zhu, B. Zhang, P. F. Sullivan, A. Chess, S. M. Purcell, L. A. Shinobu, L. M. Mangravite, H. Toyoshiba, R. E. Gur, C. G. Hahn, D. A. Lewis, V. Haroutunian, M. A. Peters, B. K. Lipska, J. D. Buxbaum, E. E. Schadt, K. Hirai, K. Roeder, K. J. Brennand, N. Katsanis, E. Domenici, B. Devlin, P. Sklar, Gene expression elucidates functional impact of polygenic risk for schizophrenia. *Nat. Neurosci.* **19**, 1442–1453 (2016).

6. A. E. Jaffe, R. E. Straub, J. H. Shin, R. Tao, Y. Gao, L. Collado-Torres, T. Kam-Thong, H. S. Xi, J. Quan, Q. Chen, C. Colantuoni, W. S. Ulrich, B. J. Maher, A. Deep-Soboslay, C. BrainSeq, A. J. Cross, N. J. Brandon, J. T. Leek, T. M. Hyde, J. E. Kleinman, D. R. Weinberger, Developmental and genetic regulation of the human cortex transcriptome illuminate schizophrenia pathogenesis. *Nat. Neurosci.* **21**, 1117–1125 (2018).

7. A. Gusev, N. Mancuso, H. Won, M. Kousi, H. K. Finucane, Y. Reshef, L. Song, A. Safi; Schizophrenia Working Group of the Psychiatric Genomics Consortium, S. McCarroll, B. M. Neale, R. A. Ophoff, M. C. O'Donovan, G. E. Crawford, D. H. Geschwind, N. Katsanis, P. F. Sullivan, B. Pasaniuc, A. L. Price, Transcriptome-wide association study of schizophrenia and chromatin activity yields mechanistic disease insights. *Nat. Genet.* **50**, 538–548 (2018).

8. L. M. Huckins, A. Dobbyn, D. M. Ruderfer, G. Hoffman, W. Wang, A. F. Pardinas, V. M. Rajagopal, T. D. Als, H. T. Nguyen, K. Girdhar, J. Boocock, P. Roussos, M. Fromer, R. Kramer, E. Domenici, E. R. Gamazon, S. Purcell; CommonMind Consortium; The Schizophrenia Working Group of the Psychiatric Genomics Consortium; iPSYCH-GEMS Schizophrenia Working Group, D. Demontis, A. D. Borglum, J. T. R. Walters, M. C. O'Donovan, P. Sullivan, M. J. Owen, B. Devlin, S. K. Sieberts, N. J. Cox, H. K. Im, P. Sklar, E. A. Stahl, Gene expression imputation across multiple brain regions provides insights into schizophrenia risk. *Nat. Genet.* **51**, 659–674 (2019).

9. N. Schrode, S. M. Ho, K. Yamamuro, A. Dobbyn, L. Huckins, M. R. Matos, E. Cheng, P. J. M. Deans, E. Flaherty, N. Barretto, A. Topol, K. Alganem, S. Abadali, J. Gregory, E. Hoelzli, H. Phatnani, V. Singh, D. Girish, B. Aronow, R. McCullumsmith, G. E. Hoffman, E. A. Stahl, H. Morishita, P. Sklar, K. J. Brennand, Synergistic effects of common schizophrenia risk variants. *Nat. Genet.* **51**, 1475–1485 (2019).

10. M. J. Gandal, J. R. Haney, N. N. Parikshak, V. Leppa, G. Ramaswami, C. Hartl, A. J. Schork, V. Appadurai, A. Buil, T. M. Werge, C. Liu, K. P. White; CommonMind Consortium; PsychENCODE Consortium; iPSYCH-BROAD Working Group, S. Horvath, D. H. Geschwind, Shared molecular neuropathology across major psychiatric disorders parallels polygenic overlap. *Science* **359**, 693–697 (2018).

11. C. L. Hartl, G. Ramaswami, W. G. Pembroke, S. Muller, G. Pintacuda, A. Saha, P. Parsana, A. Battle, K. Lage, D. H. Geschwind, Coexpression network architecture reveals the brain-wide and multiregional basis of disease susceptibility. *Nat. Neurosci.* **24**, 1313–1323 (2021).

12. E. Radulescu, A. E. Jaffe, R. E. Straub, Q. Chen, J. H. Shin, T. M. Hyde, J. E. Kleinman, D. R. Weinberger, Identification and prioritization of gene sets associated with schizophrenia risk by co-expression network analysis in human brain. *Mol. Psychiatry* **25**, 791–804 (2020).

13. G. Pergola, P. Di Carlo, E. D'Ambrosio, B. Gelao, L. Fazio, M. Papalino, A. Monda, G. Scozia, B. Pietrangelo, M. Attrotto, J. A. Apud, Q. Chen, V. S. Mattay, A. Rampino, G. Caforio, D. R. Weinberger, G. Blasi, A. Bertolino, DRD2 co-expression network and a related polygenic index predict imaging, behavioral and clinical phenotypes linked to schizophrenia. *Transl. Psychiatry* **7**, e1006 (2017).

14. G. Pergola, P. Di Carlo, A. E. Jaffe, M. Papalino, Q. Chen, T. M. Hyde, J. E. Kleinman, J. H. Shin, A. Rampino, G. Blasi, D. R. Weinberger, A. Bertolino, Prefrontal coexpression of schizophrenia risk genes is associated with treatment response in patients. *Biol. Psychiatry* **86**, 45–55 (2019).

15. U. Braun, A. Harneit, G. Pergola, T. Menara, A. Schafer, R. F. Betzel, Z. Zang, J. I. Schweiger, X. Zhang, K. Schwarz, J. Chen, G. Blasi, A. Bertolino, D. Durstewitz, F. Pasqualetti, E. Schwarz, A. Meyer-Lindenberg, D. S. Bassett, H. Tost, Brain network dynamics during working memory are modulated by dopamine and diminished in schizophrenia. *Nat. Commun.* **12**, 3478 (2021).

16. D. R. Weinberger, Implications of normal brain development for the pathogenesis of schizophrenia. *Arch. Gen. Psychiatry* **44**, 660–669 (1987).

17. E. K. Lambe, L. S. Krimer, P. S. Goldman-Rakic, Differential postnatal development of catecholamine and serotonin inputs to identified neurons in prefrontal cortex of rhesus monkey. *J. Neurosci.* **20**, 8780–8787 (2000).

18. E. Walker, R. J. Lewine, Prediction of adult-onset schizophrenia from childhood home movies of the patients. *Am. J. Psychiatry* **147**, 1052–1056 (1990).

19. D. R. Weinberger, P. J. Harrison, *Schizophrenia* (Wiley-Blackwell, 2011).

20. Q. Chen, G. Ursini, A. L. Romer, A. R. Knodt, K. Mezeivtch, E. Xiao, G. Pergola, G. Blasi, R. E. Straub, J. H. Callicott, K. F. Berman, A. R. Hariri, A. Bertolino, V. S. Mattay, D. R. Weinberger, Schizophrenia polygenic risk score predicts mnemonic hippocampal activity. *Brain* **141**, 1218–1228 (2018).

21. O. D. Howes, R. McCutcheon, M. J. Owen, R. M. Murray, The role of genes, stress, and dopamine in the development of schizophrenia. *Biol. Psychiatry* **81**, 9–20 (2017).

22. A. Abi-Dargham, From "bedside" to "bench" and back: A translational approach to studying dopamine dysfunction in schizophrenia. *Neurosci. Biobehav. Rev.* **110**, 174–179 (2020).

23. A. E. Jaffe, D. J. Hoeppner, T. Saito, L. Blanpain, J. Ukaigwe, E. E. Burke, L. Collado-Torres, R. Tao, K. Tajinda, K. R. Maynard, M. N. Tran, K. Martinowich, A. Deep-Soboslay, J. H. Shin, J. E. Kleinman, D. R. Weinberger, M. Matsumoto, T. M. Hyde, Profiling gene expression in the human dentate gyrus granule cell layer reveals insights into schizophrenia and its genetic risk. *Nat. Neurosci.* **23**, 510–519 (2020).

24. D. Cameron, D. Mi, N. N. Vinh, C. Webber, M. Li, O. Marin, M. C. O'Donovan, N. J. Bray, Single-Nuclei RNA sequencing of 5 regions of the human prenatal brain implicates developing neuron populations in genetic risk for schizophrenia. *Biol. Psychiatry*, (2022).

25. A. J. Willsey, S. J. Sanders, M. Li, S. Dong, A. T. Tebbenkamp, R. A. Muhle, S. K. Reilly, L. Lin, S. Fertuzinhos, J. A. Miller, M. T. Murtha, C. Bichsel, W. Niu, J. Cotney, A. G. Ercan-Sencicek, J. Gockley, A. R. Gupta, W. Han, X. He, E. J. Hoffman, L. Klei, J. Lei, W. Liu, L. Liu, C. Lu, X. Xu, Y. Zhu, S. M. Mane, E. S. Lein, L. Wei, J. P. Noonan, K. Roeder, B. Devlin, N. Sestan, M. W. State, Coexpression networks implicate human midfetal deep cortical projection neurons in the pathogenesis of autism. *Cell* **155**, 997–1007 (2013).

26. R. L. Walker, G. Ramaswami, C. Hartl, N. Mancuso, M. J. Gandal, L. de la Torre-Ubieta, B. Pasaniuc, J. L. Stein, D. H. Geschwind, Genetic control of expression and splicing in developing human brain informs disease mechanisms. *Cell* **179**, 750–771.e22 (2019).

27. D. M. Werling, S. Pochareddy, J. Choi, J. Y. An, B. Sheppard, M. Peng, Z. Li, C. Dastmalchi, G. Santpere, A. M. M. Sousa, A. T. N. Tebbenkamp, N. Kaur, F. O. Gulden, M. S. Breen, L. Liang, M. C. Gilson, X. Zhao, S. Dong, L. Klei, A. E. Cicek, J. D. Buxbaum, H. Adle-Biassette, J. L. Thomas, K. A. Aldinger, D. R. O'Day, I. A. Glass, N. A. Zaitlen, M. E. Talkowski, K. Roeder, M. W. State, B. Devlin, S. J. Sanders, N. Sestan, Whole-genome and RNA sequencing reveal variation and transcriptomic coordination in the developing human prefrontal cortex. *Cell Rep.* **31**, 107489 (2020).

28. N. Y. A. Sey, B. Hu, W. Mah, H. Fauni, J. C. McAfee, P. Rajarajan, K. J. Brennand, S. Akbarian, H. Won, A computational tool (H-MAGMA) for improved prediction of brain-disorder risk genes by incorporating brain chromatin interaction profiles. *Nat. Neurosci.* **23**, 583–593 (2020).

29. A. E. Jaffe, J. Shin, L. Collado-Torres, J. T. Leek, R. Tao, C. Li, Y. Gao, Y. Jia, B. J. Maher, T. M. Hyde, J. E. Kleinman, D. R. Weinberger, Developmental regulation of human cortex transcription and its clinical relevance at single base resolution. *Nat. Neurosci.* **18**, 154–161 (2015).

30. C. Colantuoni, B. K. Lipska, T. Ye, T. M. Hyde, R. Tao, J. T. Leek, E. A. Colantuoni, A. G. Elkahloun, M. M. Herman, D. R. Weinberger, J. E. Kleinman, Temporal dynamics and genetic control of transcription in the human prefrontal cortex. *Nature* **478**, 519–523 (2011).

31. M. Li, G. Santpere, Y. Imamura Kawasawa, O. V. Evgrafov, F. O. Gulden, S. Pochareddy, S. M. Sunkin, Z. Li, Y. Shin, Y. Zhu, A. M. M. Sousa, D. M. Werling, R. R. Kitchen, H. J. Kang, M. Pletikos, J. Choi, S. Muchnik, X. Xu, D. Wang, B. Lorente-Galdos, S. Liu, P. Giusti-Rodriguez, H. Won, C. A. de Leeuw, A. F. Pardinas; BrainSpan Consortium; PsychENCODE Consortium; PsychENCODE Developmental Subgroup, M. Hu, F. Jin, Y. Li, M. J. Owen, M. C. O'Donovan, J. T. R. Walters, D. Posthuma, M. A. Reimers, P. Levitt, D. R. Weinberger, T. M. Hyde, J. E. Kleinman, D. H. Geschwind, M. J. Hawrylycz, M. W. State, S.

J. Sanders, P. F. Sullivan, M. B. Gerstein, E. S. Lein, J. A. Knowles, N. Sestan, Integrative functional genomic analysis of human brain development and neuropsychiatric risks. *Science* **362**, eaat7615 (2018).

32. D. Wang, S. Liu, J. Warrell, H. Won, X. Shi, F. C. P. Navarro, D. Clarke, M. Gu, P. Emani, Y. T. Yang, M. Xu, M. J. Gandal, S. Lou, J. Zhang, J. J. Park, C. Yan, S. K. Rhie, K. Manakongtreecheep, H. Zhou, A. Nathan, M. Peters, E. Mattei, D. Fitzgerald, T. Brunetti, J. Moore, Y. Jiang, K. Girdhar, G. E. Hoffman, S. Kalayci, Z. H. Gumus, G. E. Crawford; PsychENCODE Consortium, P. Roussos, S. Akbarian, A. E. Jaffe, K. P. White, Z. Weng, N. Sestan, D. H. Geschwind, J. A. Knowles, M. B. Gerstein, Comprehensive functional genomic resource and integrative model for the human brain. *Science* **362**, eaat8464 (2018).

33. S. C. Page, S. R. Sripathy, F. Farinelli, Z. Ye, Y. Wang, D. J. Hiler, E. A. Pattie, C. V. Nguyen, M. Tippani, R. L. Moses, H.-Y. Chen, M. N. Tran, N. J. Eagles, J. M. Stolz, J. L. Catallini, O. R. Soudry, D. Dickinson, K. F. Berman, J. A. Apud, D. R. Weinberger, K. Martinowich, A. E. Jaffe, R. E. Straub, B. J. Maher, Electrophysiological measures from human iPSC-derived neurons are associated with schizophrenia clinical status and predict individual cognitive performance. *Proc. Natl. Acad. Sci. U.S.A.* **119**, e2109395119 (2022).

34. B. Zhang, S. Horvath, A general framework for weighted gene co-expression network analysis. *Stat. Appl. Genet. Mol. Biol.* **4**, Article17 (2005).

35. G. Pergola, A. Rampino, P. Di Carlo, A. Marakhovskaia, T. Quarto, L. Fazio, M. Papalino, S. Torretta, N. Amoroso, M. N. Castro, E. Domenici, J. Dukart, J. Khlghatyan, A. Monaco, T. Popolizio, R. Romano, L. Sportelli, H. Zunuer, G. Blasi, J. M. Beaulieu, A. Bertolino, A miR-137-related biological pathway of risk for schizophrenia is associated with human brain emotion processing. *bioRxiv* 2020.08.03.230227 (2020).

36. M. J. Gandal, P. Zhang, E. Hadjimichael, R. L. Walker, C. Chen, S. Liu, H. Won, H. van Bakel, M. Varghese, Y. Wang, A. W. Shieh, J. Haney, S. Parhami, J. Belmont, M. Kim, P. Moran Losada, Z. Khan, J. Mleczko, Y. Xia, R. Dai, D. Wang, Y. T. Yang, M. Xu, K. Fish, P. R. Hof, J. Warrell, D. Fitzgerald, K. White, A. E. Jaffe, E. C. Psych, M. A. Peters, M.

Gerstein, C. Liu, L. M. Iakoucheva, D. Pinto, D. H. Geschwind, Transcriptome-wide isoform-level dysregulation in ASD, schizophrenia, and bipolar disorder. *Science* **362**, (2018).

37. R. Birnbaum, D. R. Weinberger, Genetic insights into the neurodevelopmental origins of schizophrenia. *Nat. Rev. Neurosci.* **18**, 727–740 (2017).

38. R. Yurko, K. Roeder, B. Devlin, M. G'Sell, H-MAGMA, inheriting a shaky statistical foundation, yields excess false positives. *Ann. Hum. Genet.* **85**, 97–100 (2021).

39. C. de Leeuw, N. Y. A. Sey, D. Posthuma, H. Won, A response to Yurko et al: H-MAGMA, inheriting a shaky statistical foundation, yields excess false positives. *bioRxiv*, 2020.09.25.310722 (2020).

40. T. Singh, T. Poterba, D. Curtis, H. Akil, M. Al Eissa, J. D. Barchas, N. Bass, T. B. Bigdeli, G. Breen, E. J. Bromet, P. F. Buckley, W. E. Bunney, J. Bybjerg-Grauholm, W. F. Byerley, S. B. Chapman, W. J. Chen, C. Churchhouse, N. Craddock, C. M. Cusick, L. DeLisi, S. Dodge, M. A. Escamilla, S. Eskelinen, A. H. Fanous, S. V. Faraone, A. Fiorentino, L. Francioli, S. B. Gabriel, D. Gage, S. A. Gagliano Taliun, A. Ganna, G. Genovese, D. C. Glahn, J. Grove, M. H. Hall, E. Hamalainen, H. O. Heyne, M. Holi, D. M. Hougaard, D. P. Howrigan, H. Huang, H. G. Hwu, R. S. Kahn, H. M. Kang, K. J. Karczewski, G. Kirov, J. A. Knowles, F. S. Lee, D. S. Lehrer, F. Lescai, D. Malaspina, S. R. Marder, S. A. McCarroll, A. M. McIntosh, H. Medeiros, L. Milani, C. P. Morley, D. W. Morris, P. B. Mortensen, R. M. Myers, M. Nordentoft, N. L. O'Brien, A. M. Olivares, D. Ongur, W. H. Ouwehand, D. S. Palmer, T. Paunio, D. Quested, M. H. Rapaport, E. Rees, B. Rollins, F. K. Satterstrom, A. Schatzberg, E. Scolnick, L. J. Scott, S. I. Sharp, P. Sklar, J. W. Smoller, J. L. Sobell, M. Solomonson, E. A. Stahl, C. R. Stevens, J. Suvisaari, G. Tiao, S. J. Watson, N. A. Watts, D. H. Blackwood, A. D. Borglum, B. M. Cohen, A. P. Corvin, T. Esko, N. B. Freimer, S. J. Glatt, C. M. Hultman, A. McQuillin, A. Palotie, C. N. Pato, M. T. Pato, A. E. Pulver, D. St Clair, M. T. Tsuang, M. P. Vawter, J. T. Walters, T. M. Werge, R. A. Ophoff, P. F. Sullivan, M. J. Owen, M. Boehnke, M. C. O'Donovan, B. M. Neale, M. J. Daly, Rare coding variants in ten genes confer substantial risk for schizophrenia. *Nature* **604**, 509–516 (2022).

41. M. N. Tran, K. R. Maynard, A. Spangler, L. A. Huuki, K. D. Montgomery, V. Sadashivaiah, M. Tippani, B. K. Barry, D. B. Hancock, S. C. Hicks, J. E. Kleinman, T. M. Hyde, L. Collado-Torres, A. E. Jaffe, K. Martinowich, Single-nucleus transcriptome analysis reveals cell-type-specific molecular signatures across reward circuitry in the human brain. *Neuron* **109**, 3088–3103.e5 (2021).

42. D. R. Weinberger, From neuropathology to neurodevelopment. *Lancet* **346**, 552–557 (1995).

43. D. R. Weinberger, Thinking about schizophrenia in an era of genomic medicine. *Am. J. Psychiatry* **176**, 12–20 (2019).

44. H. Tost, A. Meyer-Lindenberg, Puzzling over schizophrenia: Schizophrenia, social environment and the brain. *Nat. Med.* **18**, 211–213 (2012).

45. Y. Zhu, A. M. M. Sousa, T. Gao, M. Skarica, M. Li, G. Santpere, P. Esteller-Cucala, D. Juan, L. Ferrandez-Peral, F. O. Gulden, M. Yang, D. J. Miller, T. Marques-Bonet, Y. Imamura Kawasawa, H. Zhao, N. Sestan, Spatiotemporal transcriptomic divergence across human and macaque brain development. *Science* **362**, (2018).

46. L. de la Torre-Ubieta, J. L. Stein, H. Won, C. K. Opland, D. Liang, D. Lu, D. H. Geschwind, The dynamic landscape of open chromatin during human cortical neurogenesis. *Cell* **172**, 289–304.e18 (2018).

47. H. Won, L. de la Torre-Ubieta, J. L. Stein, N. N. Parikshak, J. Huang, C. K. Opland, M. J. Gandal, G. J. Sutton, F. Hormozdiari, D. Lu, C. Lee, E. Eskin, I. Voineagu, J. Ernst, D. H. Geschwind, Chromosome conformation elucidates regulatory relationships in developing human brain. *Nature* **538**, 523–527 (2016).

48. G. Ursini, G. Punzi, Q. Chen, S. Marenco, J. F. Robinson, A. Porcelli, E. G. Hamilton, M. Mitjans, G. Maddalena, M. Begemann, J. Seidel, H. Yanamori, A. E. Jaffe, K. F. Berman, M. F. Egan, R. E. Straub, C. Colantuoni, G. Blasi, R. Hashimoto, D. Rujescu, H. Ehrenreich, A. Bertolino, D. R. Weinberger, Convergence of placenta biology and genetic risk for schizophrenia. *Nat. Med.* **24**, 792–801 (2018).

49. J. H. Callicott, A. Bertolino, V. S. Mattay, F. J. Langheim, J. Duyn, R. Coppola, T. E. Goldberg, D. R. Weinberger, Physiological dysfunction of the dorsolateral prefrontal cortex in schizophrenia revisited. *Cereb. Cortex* **10**, 1078–1092 (2000).

50. J. H. Callicott, M. F. Egan, V. S. Mattay, A. Bertolino, A. D. Bone, B. Verchinksi, D. R. Weinberger, Abnormal fMRI response of the dorsolateral prefrontal cortex in cognitively intact siblings of patients with schizophrenia. *Am. J. Psychiatry* **160**, 709–719 (2003).

51. Y. Patel, J. Shin, C. Abe, I. Agartz, C. Alloza, D. Alnaes, S. Ambrogi, L. A. Antonucci, C. Arango, V. Arolt, G. Auzias, R. Ayesa-Arriola, N. Banaj, T. Banaschewski, C. Bandeira, Z. Basgoze, R. B. Cupertino, C. H. D. Bau, J. Bauer, S. Baumeister, F. Bernardoni, A. Bertolino, C. D. M. Bonnin, D. Brandeis, S. Brem, J. Bruggemann, R. Bulow, J. R. Bustillo, S. Calderoni, R. Calvo, E. J. Canales-Rodriguez, D. M. Cannon, S. Carmona, V. J. Carr, S. V. Catts, S. Chenji, Q. H. Chew, D. Coghill, C. G. Connolly, A. Conzelmann, A. R. Craven, B. Crespo-Facorro, K. Cullen, A. Dahl, U. Dannlowski, C. G. Davey, C. Deruelle, C. M. Diaz-Caneja, K. Dohm, S. Ehrlich, J. Epstein, T. Erwin-Grabner, L. T. Eyler, J. Fedor, J. Fitzgerald, W. Foran, J. M. Ford, L. Fortea, P. Fuentes-Claramonte, J. Fullerton, L. Furlong, L. Gallagher, B. Gao, S. Gao, J. M. Goikolea, I. Gotlib, R. Goya-Maldonado, H. J. Grabe, M. Green, E. H. Grevet, N. A. Groenewold, D. Grotegerd, O. Gruber, J. Haavik, T. Hahn, B. J. Harrison, W. Heindel, F. Henskens, D. J. Heslenfeld, E. Hilland, P. J. Hoekstra, S. Hohmann, N. Holz, F. M. Howells, J. C. Ipser, N. Jahanshad, B. Jakobi, A. Jansen, J. Janssen, R. Jonassen, A. Kaiser, V. Kaleda, J. Karantonis, J. A. King, T. Kircher, P. Kochunov, S. M. Koopowitz, M. Landen, N. I. Landro, S. Lawrie, I. Lebedeva, B. Luna, A. J. Lundervold, F. P. MacMaster, L. A. Maglanoc, D. H. Mathalon, C. McDonald, A. McIntosh, S. Meinert, P. T. Michie, P. Mitchell, A. Moreno-Alcazar, B. Mowry, F. Muratori, L. Nabulsi, I. Nenadic, R. O'Gorman Tuura, J. Oosterlaan, B. Overs, C. Pantelis, M. Parellada, J. C. Pariente, P. Pauli, G. Pergola, F. M. Piarulli, F. Picon, F. Piras, E. Pomarol-Clotet, C. Pretus, Y. Quide, J. Radua, J. A. Ramos-Quiroga, P. E. Rasser, A. Reif, A. Retico, G. Roberts, S. Rossell, D. L. Rovaris, K. Rubia, M. Sacchet, J. Salavert, R. Salvador, S. Sarro, A. Sawa, U. Schall, R. Scott, P. Selvaggi, T. Silk, K. Sim, A. Skoch, G. Spalletta, F. Spaniel, D. J. Stein, O. Steinstrater, A. Stolicyn, Y. Takayanagi, L. Tamm, M. Tavares, A. Teumer, K. Thiel, S. I. Thomopoulos, D. Tomecek, A. S. Tomyshev, D. Tordesillas-Gutierrez, M. Tosetti, A. Uhlmann, T. Van

Rheenen, J. Vazquez-Bourgon, M. W. Vernooij, E. Vieta, O. Vilarroya, C. Weickert, T. Weickert, L. T. Westlye, H. Whalley, D. Willinger, A. Winter, K. Wittfeld, T. T. Yang, Y. Yoncheva, J. L. Zijlmans, M. Hoogman, B. Franke, D. van Rooij, J. Buitelaar, C. R. K. Ching, O. A. Andreassen, E. Pozzi, D. Veltman, L. Schmaal, T. G. M. van Erp, J. Turner, F. X. Castellanos, Z. Pausova, P. Thompson, T. Paus, Virtual ontogeny of cortical growth preceding mental illness. *Biol. Psychiat.* **92**, 299–313 (2022).

52. K. J. M. Benjamin, Q. Chen, A. E. Jaffe, J. M. Stolz, L. Collado-Torres, L. A. Huuki-Myers, E. E. Burke, R. Arora, A. S. Feltrin, A. R. Barbosa, E. Radulescu, G. Pergola, J. H. Shin, W. S. Ulrich, A. Deep-Soboslay, R. Tao, C. BrainSeq, T. M. Hyde, J. E. Kleinman, J. A. Erwin, D. R. Weinberger, A. C. M. Paquola, Analysis of the caudate nucleus transcriptome in individuals with schizophrenia highlights effects of antipsychotics and new risk genes. *Nat. Neurosci.* **25**, 1559–1568 (2022).

53. E. D'Ambrosio, G. Pergola, A. F. Pardinas, T. Dahoun, M. Veronese, L. Sportelli, P. Taurisano, K. Griffiths, S. Jauhar, M. Rogdaki, M. A. P. Bloomfield, S. Froudist-Walsh, I. Bonoldi, J. T. R. Walters, G. Blasi, A. Bertolino, O. D. Howes, A polygenic score indexing a *DRD2*-related co-expression network is associated with striatal dopamine function. *Sci. Rep.* **12**, 12610 (2022).

54. P. Taurisano, G. Pergola, A. Monda, L. A. Antonucci, P. Di Carlo, F. Piarulli, R. Passiatore, M. Papalino, R. Romano, A. Monaco, A. Rampino, A. Bonvino, A. Porcelli, T. Popolizio, R. Bellotti, A. Bertolino, G. Blasi, The interaction between cannabis use and a CB1-related polygenic co-expression index modulates dorsolateral prefrontal activity during working memory processing. *Brain Imaging Behav.* **15**, 288–299 (2021).

55. S. Torretta, A. Rampino, M. Basso, G. Pergola, P. Di Carlo, J. H. Shin, J. E. Kleinman, T. M. Hyde, D. R. Weinberger, R. Masellis, G. Blasi, M. Pennuto, A. Bertolino, NURR1 and ERR1 modulate the expression of genes of a DRD2 coexpression network enriched for schizophrenia risk. *J. Neurosci.* **40**, 932–941 (2020).

56. L. A. Antonucci, P. Di Carlo, R. Passiatore, M. Papalino, A. Monda, N. Amoroso, S. Tangaro, P. Taurisano, A. Rampino, F. Sambataro, T. Popolizio, A. Bertolino, G. Pergola, G.

Blasi, Thalamic connectivity measured with fMRI is associated with a polygenic index predicting thalamo-prefrontal gene co-expression. *Brain Struct. Funct.* **224**, 1331–1344 (2019).

57. P. Selvaggi, G. Pergola, B. Gelao, P. Di Carlo, M. A. Nettis, G. Amico, L. Fazio, A. Rampino, F. Sambataro, G. Blasi, A. Bertolino, Genetic variation of a DRD2 co-expression network is associated with changes in prefrontal function after D2 receptors stimulation. *Cereb. Cortex* **29**, 1162–1173 (2019).

58. L. Fazio, G. Pergola, M. Papalino, P. Di Carlo, A. Monda, B. Gelao, N. Amoroso, S. Tangaro, A. Rampino, T. Popolizio, A. Bertolino, G. Blasi, Transcriptomic context of DRD1 is associated with prefrontal activity and behavior during working memory. *Proc. Natl. Acad. Sci. U.S.A.* **115**, 5582–5587 (2018).

59. A. Monaco, A. Monda, N. Amoroso, A. Bertolino, G. Blasi, P. Di Carlo, M. Papalino, G. Pergola, S. Tangaro, R. Bellotti, A complex network approach reveals a pivotal substructure of genes linked to schizophrenia. *PLOS ONE* **13**, e0190110 (2018).

60. G. Pergola, N. Penzel, L. Sportelli, A. Bertolino, Lessons learned from parsing genetic risk for schizophrenia into biological pathways. *Biol. Psychiatry,* in press (2022)

61. L. Collado-Torres, E. E. Burke, A. Peterson, J. Shin, R. E. Straub, A. Rajpurohit, S. A. Semick, W. S. Ulrich, C. BrainSeq, A. J. Price, C. Valencia, R. Tao, A. Deep-Soboslay, T. M. Hyde, J. E. Kleinman, D. R. Weinberger, A. E. Jaffe, Regional heterogeneity in gene expression, regulation, and coherence in the frontal cortex and hippocampus across development and schizophrenia. *Neuron* **103**, 203–216.e8 (2019).

62. P. Parsana, C. Ruberman, A. E. Jaffe, M. C. Schatz, A. Battle, J. T. Leek, Addressing confounding artifacts in reconstruction of gene co-expression networks. *Genome Biol.* **20**, 94 (2019).

63. S. Freytag, J. Gagnon-Bartsch, T. P. Speed, M. Bahlo, Systematic noise degrades gene co-expression signals but can be corrected. *BMC Bioinformatics* **16**, 309 (2015).

64. A. E. Jaffe, R. Tao, A. L. Norris, M. Kealhofer, A. Nellore, J. H. Shin, D. Kim, Y. Jia, T. M. Hyde, J. E. Kleinman, R. E. Straub, J. T. Leek, D. R. Weinberger, qSVA framework for RNA quality correction in differential expression analysis. *Proc. Natl. Acad. Sci. U.S.A.* **114**, 7130–7135 (2017).

65. A. T. McKenzie, M. Wang, M. E. Hauberg, J. F. Fullard, A. Kozlenkov, A. Keenan, Y. L. Hurd, S. Dracheva, P. Casaccia, P. Roussos, B. Zhang, Brain cell type specific gene expression and co-expression network architectures. *Sci. Rep.* **8**, 8868 (2018).

66. L. Collado-Torres, A. Jaffe, E. Burke, LieberInstitute/jaffelab: Zenodo integration (v0.99.23), Zenodo (2019); https://doi.org/10.5281/zenodo.3376221.

67. S. Kumari, J. Nie, H. S. Chen, H. Ma, R. Stewart, X. Li, M. Z. Lu, W. M. Taylor, H. Wei, Evaluation of gene association methods for coexpression network construction and biological knowledge discovery. *PLOS ONE* **7**, e50411 (2012).

68. G. Yu, L. G. Wang, Y. Han, Q. Y. He, clusterProfiler: An R package for comparing biological themes among gene clusters. *OMICS* **16**, 284–287 (2012).

69. C. Schizophrenia Working Group of the Psychiatric Genomics, Biological insights from 108 schizophrenia-associated genetic loci. *Nature* **511**, 421–427 (2014).

70. D. Demontis, R. K. Walters, J. Martin, M. Mattheisen, T. D. Als, E. Agerbo, G. Baldursson, R. Belliveau, J. Bybjerg-Grauholm, M. Baekvad-Hansen, F. Cerrato, K. Chambert, C. Churchhouse, A. Dumont, N. Eriksson, M. Gandal, J. I. Goldstein, K. L. Grasby, J. Grove, O. O. Gudmundsson, C. S. Hansen, M. E. Hauberg, M. V. Hollegaard, D. P. Howrigan, H. Huang, J. B. Maller, A. R. Martin, N. G. Martin, J. Moran, J. Pallesen, D. S. Palmer, C. B. Pedersen, M. G. Pedersen, T. Poterba, J. B. Poulsen, S. Ripke, E. B. Robinson, F. K. Satterstrom, H. Stefansson, C. Stevens, P. Turley, G. B. Walters, H. Won, M. J. Wright, ADHD Working Group of the Psychiatric Genomics Consortium (PGC); Early Lifecourse & Genetic Epidemiology (EAGLE) Consortium; 23andMe Research Team, O. A. Andreassen, P. Asherson, C. L. Burton, D. I. Boomsma, B. Cormand, S. Dalsgaard, B. Franke, J. Gelernter, D. Geschwind, H. Hakonarson, J. Haavik, H. R. Kranzler, J. Kuntsi, K. Langley, K. P. Lesch,

C. Middeldorp, A. Reif, L. A. Rohde, P. Roussos, R. Schachar, P. Sklar, E. J. S. Sonuga-Barke, P. F. Sullivan, A. Thapar, J. Y. Tung, I. D. Waldman, S. E. Medland, K. Stefansson, M. Nordentoft, D. M. Hougaard, T. Werge, O. Mors, P. B. Mortensen, M. J. Daly, S. V. Faraone, A. D. Borglum, B. M. Neale, Discovery of the first genome-wide significant risk loci for attention deficit/hyperactivity disorder. *Nat. Genet.* **51**, 63–75 (2019).

71. J. Grove, S. Ripke, T. D. Als, M. Mattheisen, R. K. Walters, H. Won, J. Pallesen, E. Agerbo, O. A. Andreassen, R. Anney, S. Awashti, R. Belliveau, F. Bettella, J. D. Buxbaum, J. Bybjerg-Grauholm, M. Baekvad-Hansen, F. Cerrato, K. Chambert, J. H. Christensen, C. Churchhouse, K. Dellenvall, D. Demontis, S. De Rubeis, B. Devlin, S. Djurovic, A. L. Dumont, J. I. Goldstein, C. S. Hansen, M. E. Hauberg, M. V. Hollegaard, S. Hope, D. P. Howrigan, H. Huang, C. M. Hultman, L. Klei, J. Maller, J. Martin, A. R. Martin, J. L. Moran, M. Nyegaard, T. Naerland, D. S. Palmer, A. Palotie, C. B. Pedersen, M. G. Pedersen, T. dPoterba, J. B. Poulsen, B. S. Pourcain, P. Qvist, K. Rehnstrom, A. Reichenberg, J. Reichert, E. B. Robinson, K. Roeder, P. Roussos, E. Saemundsen, S. Sandin, F. K. Satterstrom, G. D. Smith, H. Stefansson, S. Steinberg, C. R. Stevens, P. F. Sullivan, P. Turley, G. B. Walters, X. Xu, Autism Spectrum Disorder Working Group of the Psychiatric Genomics Consortium; BUPGEN; Major Depressive Disorder Working Group of the Psychiatric Genomics Consortium; 23andMe Research Team, K. Stefansson, D. H. Geschwind, M. Nordentoft, D. M. Hougaard, T. Werge, O. Mors, P. B. Mortensen, B. M. Neale, M. J. Daly, A. D. Borglum, Identification of common genetic risk variants for autism spectrum disorder. *Nat. Genet.* **51**, 431–444 (2019).

72. E. A. Stahl, G. Breen, A. J. Forstner, A. McQuillin, S. Ripke, V. Trubetskoy, M. Mattheisen, Y. Wang, J. R. I. Coleman, H. A. Gaspar, C. A. de Leeuw, S. Steinberg, J. M. W. Pavlides, M. Trzaskowski, E. M. Byrne, T. H. Pers, P. A. Holmans, A. L. Richards, L. Abbott, E. Agerbo, H. Akil, D. Albani, N. Alliey-Rodriguez, T. D. Als, A. Anjorin, V. Antilla, S. Awasthi, J. A. Badner, M. Baekvad-Hansen, J. D. Barchas, N. Bass, M. Bauer, R. Belliveau, S. E. Bergen, C. B. Pedersen, E. Boen, M. P. Boks, J. Boocock, M. Budde, W. Bunney, M. Burmeister, J. Bybjerg-Grauholm, W. Byerley, M. Casas, F. Cerrato, P. Cervantes, K. Chambert, A. W. Charney, D. Chen, C. Churchhouse, T. K. Clarke, W. Coryell, D. W. Craig, C. Cruceanu, D. Curtis, P. M. Czerski, A. M. Dale, S. de Jong, F. Degenhardt, J. Del-Favero, J. R. DePaulo, S.

Djurovic, A. L. Dobbyn, A. Dumont, T. Elvsashagen, V. Escott-Price, C. C. Fan, S. B. Fischer, M. Flickinger, T. M. Foroud, L. Forty, J. Frank, C. Fraser, N. B. Freimer, L. Frisen, K. Gade, D. Gage, J. Garnham, C. Giambartolomei, M. G. Pedersen, J. Goldstein, S. D. Gordon, K. Gordon-Smith, E. K. Green, M. J. Green, T. A. Greenwood, J. Grove, W. Guan, J. Guzman-Parra, M. L. Hamshere, M. Hautzinger, U. Heilbronner, S. Herms, M. Hipolito, P. Hoffmann, D. Holland, L. Huckins, S. Jamain, J. S. Johnson, A. Jureus, R. Kandaswamy, R. Karlsson, J. L. Kennedy, S. Kittel-Schneider, J. A. Knowles, M. Kogevinas, A. C. Koller, R. Kupka, C. Lavebratt, J. Lawrence, W. B. Lawson, M. Leber, P. H. Lee, S. E. Levy, J. Z. Li, C. Liu, S. Lucae, A. Maaser, D. J. MacIntyre, P. B. Mahon, W. Maier, L. Martinsson, S. McCarroll, P. McGuffin, M. G. McInnis, J. D. McKay, H. Medeiros, S. E. Medland, F. Meng, L. Milani, G. W. Montgomery, D. W. Morris, T. W. Muhleisen, N. Mullins, H. Nguyen, C. M. Nievergelt, A. N. Adolfsson, E. A. Nwulia, C. O'Donovan, L. M. O. Loohuis, A. P. S. Ori, L. Oruc, U. Osby, R. H. Perlis, A. Perry, A. Pfennig, J. B. Potash, S. M. Purcell, E. J. Regeer, A. Reif, C. S. Reinbold, J. P. Rice, F. Rivas, M. Rivera, P. Roussos, D. M. Ruderfer, E. Ryu, C. Sanchez-Mora, A. F. Schatzberg, W. A. Scheftner, N. J. Schork, C. Shannon Weickert, T. Shehktman, P. D. Shilling, E. Sigurdsson, C. Slaney, O. B. Smeland, J. L. Sobell, C. Soholm Hansen, A. T. Spijker, D. St Clair, M. Steffens, J. S. Strauss, F. Streit, J. Strohmaier, S. Szelinger, R. C. Thompson, T. E. Thorgeirsson, J. Treutlein, H. Vedder, W. Wang, S. J. Watson, T. W. Weickert, S. H. Witt, S. Xi, W. Xu, A. H. Young, P. Zandi, P. Zhang, S. Zollner; eQTLGen Consortium; BIOS Consortium, R. Adolfsson, I. Agartz, M. Alda, L. Backlund, B. T. Baune, F. Bellivier, W. H. Berrettini, J. M. Biernacka, D. H. R. Blackwood, M. Boehnke, A. D. Borglum, A. Corvin, N. Craddock, M. J. Daly, U. Dannlowski, T. Esko, B. Etain, M. Frye, J. M. Fullerton, E. S. Gershon, M. Gill, F. Goes, M. Grigoroiu-Serbanescu, J. Hauser, D. M. Hougaard, C. M. Hultman, I. Jones, L. A. Jones, R. S. Kahn, G. Kirov, M. Landen, M. Leboyer, C. M. Lewis, Q. S. Li, J. Lissowska, N. G. Martin, F. Mayoral, S. L. McElroy, A. M. McIntosh, F. J. McMahon, I. Melle, A. Metspalu, P. B. Mitchell, G. Morken, O. Mors, P. B. Mortensen, B. Muller-Myhsok, R. M. Myers, B. M. Neale, V. Nimgaonkar, M. Nordentoft, M. M. Nothen, M. C. O'Donovan, K. J. Oedegaard, M. J. Owen, S. A. Paciga, C. Pato, M. T. Pato, D. Posthuma, J. A. Ramos-Quiroga, M. Ribases, M. Rietschel, G. A. Rouleau, M. Schalling, P. R. Schofield, T. G. Schulze, A. Serretti, J. W. Smoller, H. Stefansson, K. Stefansson, E. Stordal, P. F. Sullivan, G. Turecki, A. E. Vaaler, E. Vieta, J. B.

Vincent, T. Werge, J. I. Nurnberger, N. R. Wray, A. Di Florio, H. J. Edenberg, S. Cichon, R. A. Ophoff, L. J. Scott, O. A. Andreassen, J. Kelsoe, P. Sklar; Bipolar Disorder Working Group of the Psychiatric Genomics Consortium, Genome-wide association study identifies 30 loci associated with bipolar disorder. *Nat. Genet.* **51**, 793–803 (2019).

73. D. M. Howard, M. J. Adams, T. K. Clarke, J. D. Hafferty, J. Gibson, M. Shirali, J. R. I. Coleman, S. P. Hagenaars, J. Ward, E. M. Wigmore, C. Alloza, X. Shen, M. C. Barbu, E. Y. Xu, H. C. Whalley, R. E. Marioni, D. J. Porteous, G. Davies, I. J. Deary, G. Hemani, K. Berger, H. Teismann, R. Rawal, V. Arolt, B. T. Baune, U. Dannlowski, K. Domschke, C. Tian, D. A. Hinds; 23andMe Research Team; Major Depressive Disorder Working Group of the Psychiatric Genomics Consortium, M. Trzaskowski, E. M. Byrne, S. Ripke, D. J. Smith, P. F. Sullivan, N. R. Wray, G. Breen, C. M. Lewis, A. M. McIntosh, Genome-wide meta-analysis of depression identifies 102 independent variants and highlights the importance of the prefrontal brain regions. *Nat. Neurosci.* **22**, 343–352 (2019).

74. International Obsessive Compulsive Disorder Foundation Genetics Collaborative (IOCDF-GC) and OCD Collaborative Genetics Association Studies (OCGAS), Revealing the complex genetic architecture of obsessive-compulsive disorder using meta-analysis. *Mol. Psychiatry* **23**, 1181–1188 (2018).

75. C. M. Nievergelt, A. X. Maihofer, T. Klengel, E. G. Atkinson, C. Y. Chen, K. W. Choi, J. R. I. Coleman, S. Dalvie, L. E. Duncan, J. Gelernter, D. F. Levey, M. W. Logue, R. Polimanti, A. C. Provost, A. Ratanatharathorn, M. B. Stein, K. Torres, A. E. Aiello, L. M. Almli, A. B. Amstadter, S. B. Andersen, O. A. Andreassen, P. A. Arbisi, A. E. Ashley-Koch, S. B. Austin, E. Avdibegovic, D. Babic, M. Baekvad-Hansen, D. G. Baker, J. C. Beckham, L. J. Bierut, J. I. Bisson, M. P. Boks, E. A. Bolger, A. D. Borglum, B. Bradley, M. Brashear, G. Breen, R. A. Bryant, A. C. Bustamante, J. Bybjerg-Grauholm, J. R. Calabrese, J. M. Caldas-de-Almeida, A. M. Dale, M. J. Daly, N. P. Daskalakis, J. Deckert, D. L. Delahanty, M. F. Dennis, S. G. Disner, K. Domschke, A. Dzubur-Kulenovic, C. R. Erbes, A. Evans, L. A. Farrer, N. C. Feeny, J. D. Flory, D. Forbes, C. E. Franz, S. Galea, M. E. Garrett, B. Gelaye, E. Geuze, C. Gillespie, A. G. Uka, S. D. Gordon, G. Guffanti, R. Hammamieh, S. Harnal, M. A. Hauser, A. C. Heath, S. M. J. Hemmings, D. M. Hougaard, M. Jakovljevic, M. Jett, E. O. Johnson, I.

Jones, T. Jovanovic, X. J. Qin, A. G. Junglen, K. I. Karstoft, M. L. Kaufman, R. C. Kessler, A. Khan, N. A. Kimbrel, A. P. King, N. Koen, H. R. Kranzler, W. S. Kremen, B. R. Lawford, L. A. M. Lebois, C. E. Lewis, S. D. Linnstaedt, A. Lori, B. Lugonja, J. J. Luykx, M. J. Lyons, J. Maples-Keller, C. Marmar, A. R. Martin, N. G. Martin, D. Maurer, M. R. Mavissakalian, A. McFarlane, R. E. McGlinchey, K. A. McLaughlin, S. A. McLean, S. McLeay, D. Mehta, W. P. Milberg, M. W. Miller, R. A. Morey, C. P. Morris, O. Mors, P. B. Mortensen, B. M. Neale, E. C. Nelson, M. Nordentoft, S. B. Norman, M. O'Donnell, H. K. Orcutt, M. S. Panizzon, E. S. Peters, A. L. Peterson, M. Peverill, R. H. Pietrzak, M. A. Polusny, J. P. Rice, S. Ripke, V. B. Risbrough, A. L. Roberts, A. O. Rothbaum, B. O. Rothbaum, P. Roy-Byrne, K. Ruggiero, A. Rung, B. P. F. Rutten, N. L. Saccone, S. E. Sanchez, D. Schijven, S. Seedat, A. V. Seligowski, J. S. Seng, C. M. Sheerin, D. Silove, A. K. Smith, J. W. Smoller, S. R. Sponheim, D. J. Stein, J. S. Stevens, J. A. Sumner, M. H. Teicher, W. K. Thompson, E. Trapido, M. Uddin, R. J. Ursano, L. L. van den Heuvel, M. Van Hooff, E. Vermetten, C. H. Vinkers, J. Voisey, Y. Wang, Z. Wang, T. Werge, M. A. Williams, D. E. Williamson, S. Winternitz, C. Wolf, E. J. Wolf, J. D. Wolff, R. Yehuda, R. M. Young, K. A. Young, H. Zhao, L. A. Zoellner, I. Liberzon, K. J. Ressler, M. Haas, K. C. Koenen, International meta-analysis of PTSD genome-wide association studies identifies sex- and ancestry-specific genetic risk loci. *Nat. Commun.* **10**, 4558 (2019).

76. A. Erlangsen, V. Appadurai, Y. Wang, G. Turecki, O. Mors, T. Werge, P. B. Mortensen, A. Starnawska, A. D. Borglum, A. Schork, R. Nudel, M. Baekvad-Hansen, J. Bybjerg-Grauholm, D. M. Hougaard, W. K. Thompson, M. Nordentoft, E. Agerbo, Genetics of suicide attempts in individuals with and without mental disorders: a population-based genome-wide association study. *Mol. Psychiatry* **25**, 2410–2421 (2020).

77. I. E. Jansen, J. E. Savage, K. Watanabe, J. Bryois, D. M. Williams, S. Steinberg, J. Sealock, I. K. Karlsson, S. Hagg, L. Athanasiu, N. Voyle, P. Proitsi, A. Witoelar, S. Stringer, D. Aarsland, I. S. Almdahl, F. Andersen, S. Bergh, F. Bettella, S. Bjornsson, A. Braekhus, G. Brathen, C. de Leeuw, R. S. Desikan, S. Djurovic, L. Dumitrescu, T. Fladby, T. J. Hohman, P. V. Jonsson, S. J. Kiddle, A. Rongve, I. Saltvedt, S. B. Sando, G. Selbaek, M. Shoai, N. G. Skene, J. Snaedal, E. Stordal, I. D. Ulstein, Y. Wang, L. R. White, J. Hardy, J. Hjerling-Leffler, P. F. Sullivan, W. M. van der Flier, R. Dobson, L. K. Davis, H. Stefansson, K.

Stefansson, N. L. Pedersen, S. Ripke, O. A. Andreassen, D. Posthuma, Genome-wide meta-analysis identifies new loci and functional pathways influencing Alzheimer's disease risk. *Nat. Genet.* **51**, 404–413 (2019).

78. W. van Rheenen, A. Shatunov, A. M. Dekker, R. L. McLaughlin, F. P. Diekstra, S. L. Pulit, R. A. van der Spek, U. Vosa, S. de Jong, M. R. Robinson, J. Yang, I. Fogh, P. T. van Doormaal, G. H. Tazelaar, M. Koppers, A. M. Blokhuis, W. Sproviero, A. R. Jones, K. P. Kenna, K. R. van Eijk, O. Harschnitz, R. D. Schellevis, W. J. Brands, J. Medic, A. Menelaou, A. Vajda, N. Ticozzi, K. Lin, B. Rogelj, K. Vrabec, M. Ravnik-Glavac, B. Koritnik, J. Zidar, L. Leonardis, L. D. Groselj, S. Millecamps, F. Salachas, V. Meininger, M. de Carvalho, S. Pinto, J. S. Mora, R. Rojas-Garcia, M. Polak, S. Chandran, S. Colville, R. Swingler, K. E. Morrison, P. J. Shaw, J. Hardy, R. W. Orrell, A. Pittman, K. Sidle, P. Fratta, A. Malaspina, S. Topp, S. Petri, S. Abdulla, C. Drepper, M. Sendtner, T. Meyer, R. A. Ophoff, K. A. Staats, M. Wiedau-Pazos, C. Lomen-Hoerth, V. M. Van Deerlin, J. Q. Trojanowski, L. Elman, L. McCluskey, A. N. Basak, C. Tunca, H. Hamzeiy, Y. Parman, T. Meitinger, P. Lichtner, M. Radivojkov-Blagojevic, C. R. Andres, C. Maurel, G. Bensimon, B. Landwehrmeyer, A. Brice, C. A. Payan, S. Saker-Delye, A. Durr, N. W. Wood, L. Tittmann, W. Lieb, A. Franke, M. Rietschel, S. Cichon, M. M. Nothen, P. Amouyel, C. Tzourio, J. F. Dartigues, A. G. Uitterlinden, F. Rivadeneira, K. Estrada, A. Hofman, C. Curtis, H. M. Blauw, A. J. van der Kooi, M. de Visser, A. Goris, M. Weber, C. E. Shaw, B. N. Smith, O. Pansarasa, C. Cereda, R. Del Bo, G. P. Comi, S. D'Alfonso, C. Bertolin, G. Soraru, L. Mazzini, V. Pensato, C. Gellera, C. Tiloca, A. Ratti, A. Calvo, C. Moglia, M. Brunetti, S. Arcuti, R. Capozzo, C. Zecca, C. Lunetta, S. Penco, N. Riva, A. Padovani, M. Filosto, B. Muller, R. J. Stuit, PARALS Registry; SLALOM Group; SLAP Registry; FALS Sequencing Consortium; SLAGEN Consortium; NNIPPS Study Group, I. Blair, K. Zhang, E. P. McCann, J. A. Fifita, G. A. Nicholson, D. B. Rowe, R. Pamphlett, M. C. Kiernan, J. Grosskreutz, O. W. Witte, T. Ringer, T. Prell, B. Stubendorff, I. Kurth, C. A. Hubner, P. N. Leigh, F. Casale, A. Chio, E. Beghi, E. Pupillo, R. Tortelli, G. Logroscino, J. Powell, A. C. Ludolph, J. H. Weishaupt, W. Robberecht, P. Van Damme, L. Franke, T. H. Pers, R. H. Brown, J. D. Glass, J. E. Landers, O. Hardiman, P. M. Andersen, P. Corcia, P. Vourc'h, V. Silani, N. R. Wray, P. M. Visscher, P. I. de Bakker, M. A. van Es, R. J. Pasterkamp, C. M. Lewis, G. Breen, A. Al-Chalabi, L. H. van

den Berg, J. H. Veldink, Genome-wide association analyses identify new risk variants and the genetic architecture of amyotrophic lateral sclerosis. *Nat. Genet.* **48**, 1043–1048 (2016).

79. T. F. Andlauer, D. Buck, G. Antony, A. Bayas, L. Bechmann, A. Berthele, A. Chan, C. Gasperi, R. Gold, C. Graetz, J. Haas, M. Hecker, C. Infante-Duarte, M. Knop, T. Kumpfel, V. Limmroth, R. A. Linker, V. Loleit, F. Luessi, S. G. Meuth, M. Muhlau, S. Nischwitz, F. Paul, M. Putz, T. Ruck, A. Salmen, M. Stangel, J. P. Stellmann, K. H. Sturner, B. Tackenberg, F. Then Bergh, H. Tumani, C. Warnke, F. Weber, H. Wiendl, B. Wildemann, U. K. Zettl, U. Ziemann, F. Zipp, J. Arloth, P. Weber, M. Radivojkov-Blagojevic, M. O. Scheinhardt, T. Dankowski, T. Bettecken, P. Lichtner, D. Czamara, T. Carrillo-Roa, E. B. Binder, K. Berger, L. Bertram, A. Franke, C. Gieger, S. Herms, G. Homuth, M. Ising, K. H. Jockel, T. Kacprowski, S. Kloiber, M. Laudes, W. Lieb, C. M. Lill, S. Lucae, T. Meitinger, S. Moebus, M. Muller-Nurasyid, M. M. Nothen, A. Petersmann, R. Rawal, U. Schminke, K. Strauch, H. Volzke, M. Waldenberger, J. Wellmann, E. Porcu, A. Mulas, M. Pitzalis, C. Sidore, I. Zara, F. Cucca, M. Zoledziewska, A. Ziegler, B. Hemmer, B. Muller-Myhsok, Novel multiple sclerosis susceptibility loci implicated in epigenetic regulation. *Sci. Adv.* **2**, e1501678 (2016).

80. M. A. Nalls, C. Blauwendraat, C. L. Vallerga, K. Heilbron, S. Bandres-Ciga, D. Chang, M. Tan, D. A. Kia, A. J. Noyce, A. Xue, J. Bras, E. Young, R. von Coelln, J. Simon-Sanchez, C. Schulte, M. Sharma, L. Krohn, L. Pihlstrom, A. Siitonen, H. Iwaki, H. Leonard, F. Faghri, J. R. Gibbs, D. G. Hernandez, S. W. Scholz, J. A. Botia, M. Martinez, J. C. Corvol, S. Lesage, J. Jankovic, L. M. Shulman, M. Sutherland, P. Tienari, K. Majamaa, M. Toft, O. A. Andreassen, T. Bangale, A. Brice, J. Yang, Z. Gan-Or, T. Gasser, P. Heutink, J. M. Shulman, N. W. Wood, D. A. Hinds, J. A. Hardy, H. R. Morris, J. Gratten, P. M. Visscher, R. R. Graham, A. B. Singleton; 23andMe Research Team; System Genomics of Parkinson's Disease Consortium; International Parkinson's Disease Genomics Consortium, Identification of novel risk loci, causal insights, and heritable risk for Parkinson's disease: A meta-analysis of genome-wide association studies. *Lancet Neurol.* **18**, 1091–1102 (2019).

81. J. Z. Liu, S. van Sommeren, H. Huang, S. C. Ng, R. Alberts, A. Takahashi, S. Ripke, J. C. Lee, L. Jostins, T. Shah, S. Abedian, J. H. Cheon, J. Cho, N. E. Dayani, L. Franke, Y. Fuyuno, A. Hart, R. C. Juyal, G. Juyal, W. H. Kim, A. P. Morris, H. Poustchi, W. G. Newman, V.

Midha, T. R. Orchard, H. Vahedi, A. Sood, J. Y. Sung, R. Malekzadeh, H. J. Westra, K. Yamazaki, S. K. Yang; International Multiple Sclerosis Genetics Consortium; International IBD Genetics Consortium, J. C. Barrett, B. Z. Alizadeh, M. Parkes, T. B. K, M. J. Daly, M. Kubo, C. A. Anderson, R. K. Weersma, Association analyses identify 38 susceptibility loci for inflammatory bowel disease and highlight shared genetic risk across populations. *Nat. Genet.* **47**, 979–986 (2015).

82. Y. Okada, D. Wu, G. Trynka, T. Raj, C. Terao, K. Ikari, Y. Kochi, K. Ohmura, A. Suzuki, S. Yoshida, R. R. Graham, A. Manoharan, W. Ortmann, T. Bhangale, J. C. Denny, R. J. Carroll, A. E. Eyler, J. D. Greenberg, J. M. Kremer, D. A. Pappas, L. Jiang, J. Yin, L. Ye, D. F. Su, J. Yang, G. Xie, E. Keystone, H. J. Westra, T. Esko, A. Metspalu, X. Zhou, N. Gupta, D. Mirel, E. A. Stahl, D. Diogo, J. Cui, K. Liao, M. H. Guo, K. Myouzen, T. Kawaguchi, M. J. Coenen, P. L. van Riel, M. A. van de Laar, H. J. Guchelaar, T. W. Huizinga, P. Dieude, X. Mariette, S. L. Bridges, Jr., A. Zhernakova, R. E. Toes, P. P. Tak, C. Miceli-Richard, S. Y. Bang, H. S. Lee, J. Martin, M. A. Gonzalez-Gay, L. Rodriguez-Rodriguez, S. Rantapaa-Dahlqvist, L. Arlestig, H. K. Choi, Y. Kamatani, P. Galan, M. Lathrop; RACI consortium; GARNET consortium, S. Eyre, J. Bowes, A. Barton, N. de Vries, L. W. Moreland, L. A. Criswell, E. W. Karlson, A. Taniguchi, R. Yamada, M. Kubo, J. S. Liu, S. C. Bae, J. Worthington, L. Padyukov, L. Klareskog, P. K. Gregersen, S. Raychaudhuri, B. E. Stranger, P. L. De Jager, L. Franke, P. M. Visscher, M. A. Brown, H. Yamanaka, T. Mimori, A. Takahashi, H. Xu, T. W. Behrens, K. A. Siminovitch, S. Momohara, F. Matsuda, K. Yamamoto, R. M. Plenge, Genetics of rheumatoid arthritis contributes to biology and drug discovery. *Nature* **506**, 376–381 (2014).

83. N. G. Skene, J. Bryois, T. E. Bakken, G. Breen, J. J. Crowley, H. A. Gaspar, P. Giusti-Rodriguez, R. D. Hodge, J. A. Miller, A. B. Munoz-Manchado, M. C. O'Donovan, M. J. Owen, A. F. Pardinas, J. Ryge, J. T. R. Walters, S. Linnarsson, E. S. Lein; Major Depressive Disorder Working Group of the Psychiatric Genomics Consortium, P. F. Sullivan, J. Hjerling-Leffler, Genetic identification of brain cell types underlying schizophrenia. *Nat. Genet.* **50**, 825–833 (2018).

84. N. Habib, I. Avraham-Davidi, A. Basu, T. Burks, K. Shekhar, M. Hofree, S. R. Choudhury, F. Aguet, E. Gelfand, K. Ardlie, D. A. Weitz, O. Rozenblatt-Rosen, F. Zhang, A. Regev, Massively parallel single-nucleus RNA-seq with DroNc-seq. *Nat. Methods* **14**, 955–958 (2017).

85. M. E. Ritchie, B. Phipson, D. Wu, Y. Hu, C. W. Law, W. Shi, G. K. Smyth, limma powers differential expression analyses for RNA-sequencing and microarray studies. *Nucleic Acids Res.* **43**, e47 (2015).

86. P. D. Thomas, M. J. Campbell, A. Kejariwal, H. Mi, B. Karlak, R. Daverman, K. Diemer, A. Muruganujan, A. Narechania, PANTHER: A library of protein families and subfamilies indexed by function. *Genome Res.* **13**, 2129–2141 (2003).

87. L. S. Hall, C. W. Medway, O. Pain, A. F. Pardinas, E. G. Rees, V. Escott-Price, A. Pocklington, N. J. Bray, P. A. Holmans, J. T. R. Walters, M. J. Owen, M. C. O'Donovan, A transcriptome-wide association study implicates specific pre- and post-synaptic abnormalities in schizophrenia. *Hum. Mol. Genet.* **29**, 159–167 (2020).

88. A. M. M. Sousa, Y. Zhu, M. A. Raghanti, R. R. Kitchen, M. Onorati, A. T. N. Tebbenkamp, B. Stutz, K. A. Meyer, M. Li, Y. I. Kawasawa, F. Liu, R. G. Perez, M. Mele, T. Carvalho, M. Skarica, F. O. Gulden, M. Pletikos, A. Shibata, A. R. Stephenson, M. K. Edler, J. J. Ely, J. D. Elsworth, T. L. Horvath, P. R. Hof, T. M. Hyde, J. E. Kleinman, D. R. Weinberger, M. Reimers, R. P. Lifton, S. M. Mane, J. P. Noonan, M. W. State, E. S. Lein, J. A. Knowles, T. Marques-Bonet, C. C. Sherwood, M. B. Gerstein, N. Sestan, Molecular and cellular reorganization of neural circuits in the human lineage. *Science* **358**, 1027–1032 (2017).

89. E. Hannon, E. Dempster, J. Viana, J. Burrage, A. R. Smith, R. Macdonald, D. St Clair, C. Mustard, G. Breen, S. Therman, J. Kaprio, T. Toulopoulou, H. E. Hulshoff Pol, M. M. Bohlken, R. S. Kahn, I. Nenadic, C. M. Hultman, R. M. Murray, D. A. Collier, N. Bass, H. Gurling, A. McQuillin, L. Schalkwyk, J. Mill, An integrated genetic-epigenetic analysis of schizophrenia: Evidence for co-localization of genetic associations and differential DNA methylation. *Genome Biol.* **17**, 176 (2016).

90. A. E. Jaffe, Y. Gao, A. Deep-Soboslay, R. Tao, T. M. Hyde, D. R. Weinberger, J. E. Kleinman, Mapping DNA methylation across development, genotype and schizophrenia in the human frontal cortex. *Nat. Neurosci.* **19**, 40–47 (2016).

91. M. Kinoshita, S. Numata, A. Tajima, K. Ohi, R. Hashimoto, S. Shimodera, I. Imoto, M. Takeda, T. Ohmori, Aberrant DNA methylation of blood in schizophrenia by adjusting for estimated cellular proportions. *Neuromolecular Med.* **16**, 697–703 (2014).

92. C. Montano, M. A. Taub, A. Jaffe, E. Briem, J. I. Feinberg, R. Trygvadottir, A. Idrizi, A. Runarsson, B. Berndsen, R. C. Gur, T. M. Moore, R. T. Perry, D. Fugman, S. Sabunciyan, R. H. Yolken, T. M. Hyde, J. E. Kleinman, J. L. Sobell, C. N. Pato, M. T. Pato, R. C. Go, V. Nimgaonkar, D. R. Weinberger, D. Braff, R. E. Gur, M. D. Fallin, A. P. Feinberg, Association of DNA methylation differences with schizophrenia in an epigenome-wide association study. *JAMA Psychiatry* **73**, 506–514 (2016).

93. S. Numata, T. Ye, M. Herman, B. K. Lipska, DNA methylation changes in the postmortem dorsolateral prefrontal cortex of patients with schizophrenia. *Front. Genet.* **5**, 280 (2014).

94. L. F. Wockner, E. P. Noble, B. R. Lawford, R. M. Young, C. P. Morris, V. L. Whitehall, J. Voisey, Genome-wide DNA methylation analysis of human brain tissue from schizophrenia patients. *Transl. Psychiatry* **4**, e339 (2014).

95. C. Finan, A. Gaulton, F. A. Kruger, R. T. Lumbers, T. Shah, J. Engmann, L. Galver, R. Kelley, A. Karlsson, R. Santos, J. P. Overington, A. D. Hingorani, J. P. Casas, The druggable genome and support for target identification and validation in drug development. *Sci. Transl. Med.* **9**, (2017).

96. R. Santos, O. Ursu, A. Gaulton, A. P. Bento, R. S. Donadi, C. G. Bologa, A. Karlsson, B. Al-Lazikani, A. Hersey, T. I. Oprea, J. P. Overington, A comprehensive map of molecular drug targets. *Nat. Rev. Drug Discov.* **16**, 19–34 (2017).

97. T. Sheils, S. L. Mathias, V. B. Siramshetty, G. Bocci, C. G. Bologa, J. J. Yang, A. Waller, N. Southall, D. T. Nguyen, T. I. Oprea, How to illuminate the druggable genome using Pharos. *Curr. Protoc. Bioinformatics* **69**, e92 (2020).

98. Q. Wang, R. Chen, F. Cheng, Q. Wei, Y. Ji, H. Yang, X. Zhong, R. Tao, Z. Wen, J. S. Sutcliffe, C. Liu, E. H. Cook, N. J. Cox, B. Li, A Bayesian framework that integrates multi-omics data and gene networks predicts risk genes from schizophrenia GWAS data. *Nat. Neurosci.* **22**, 691–699 (2019).

99. M. Lek, K. J. Karczewski, E. V. Minikel, K. E. Samocha, E. Banks, T. Fennell, A. H. O'Donnell-Luria, J. S. Ware, A. J. Hill, B. B. Cummings, T. Tukiainen, D. P. Birnbaum, J. A. Kosmicki, L. E. Duncan, K. Estrada, F. Zhao, J. Zou, E. Pierce-Hoffman, J. Berghout, D. N. Cooper, N. Deflaux, M. DePristo, R. Do, J. Flannick, M. Fromer, L. Gauthier, J. Goldstein, N. Gupta, D. Howrigan, A. Kiezun, M. I. Kurki, A. L. Moonshine, P. Natarajan, L. Orozco, G. M. Peloso, R. Poplin, M. A. Rivas, V. Ruano-Rubio, S. A. Rose, D. M. Ruderfer, K. Shakir, P. D. Stenson, C. Stevens, B. P. Thomas, G. Tiao, M. T. Tusie-Luna, B. Weisburd, H. H. Won, D. Yu, D. M. Altshuler, D. Ardissino, M. Boehnke, J. Danesh, S. Donnelly, R. Elosua, J. C. Florez, S. B. Gabriel, G. Getz, S. J. Glatt, C. M. Hultman, S. Kathiresan, M. Laakso, S. McCarroll, M. I. McCarthy, D. McGovern, R. McPherson, B. M. Neale, A. Palotie, S. M. Purcell, D. Saleheen, J. M. Scharf, P. Sklar, P. F. Sullivan, J. Tuomilehto, M. T. Tsuang, H. C. Watkins, J. G. Wilson, M. J. Daly, D. G. MacArthur; Exome Aggregation Consortium, Analysis of protein-coding genetic variation in 60,706 humans. *Nature* **536**, 285–291 (2016).

100. M. B. Kursa, W. R. Rudnicki, Feature selection with the Boruta package. *J. Stat. Softw.* **36**, 1–13 (2010).

101. J. A. Miller, S. L. Ding, S. M. Sunkin, K. A. Smith, L. Ng, A. Szafer, A. Ebbert, Z. L. Riley, J. J. Royall, K. Aiona, J. M. Arnold, C. Bennet, D. Bertagnolli, K. Brouner, S. Butler, S. Caldejon, A. Carey, C. Cuhaciyan, R. A. Dalley, N. Dee, T. A. Dolbeare, B. A. Facer, D. Feng, T. P. Fliss, G. Gee, J. Goldy, L. Gourley, B. W. Gregor, G. Gu, R. E. Howard, J. M. Jochim, C. L. Kuan, C. Lau, C. K. Lee, F. Lee, T. A. Lemon, P. Lesnar, B. McMurray, N. Mastan, N. Mosqueda, T. Naluai-Cecchini, N. K. Ngo, J. Nyhus, A. Oldre, E. Olson, J.

Parente, P. D. Parker, S. E. Parry, A. Stevens, M. Pletikos, M. Reding, K. Roll, D. Sandman, M. Sarreal, S. Shapouri, N. V. Shapovalova, E. H. Shen, N. Sjoquist, C. R. Slaughterbeck, M. Smith, A. J. Sodt, D. Williams, L. Zollei, B. Fischl, M. B. Gerstein, D. H. Geschwind, I. A. Glass, M. J. Hawrylycz, R. F. Hevner, H. Huang, A. R. Jones, J. A. Knowles, P. Levitt, J. W. Phillips, N. Sestan, P. Wohnoutka, C. Dang, A. Bernard, J. G. Hohmann, E. S. Lein, Transcriptional landscape of the prenatal human brain. *Nature* **508**, 199–206 (2014).

102. Z. Gu, R. Eils, M. Schlesner, Complex heatmaps reveal patterns and correlations in multidimensional genomic data. *Bioinformatics* **32**, 2847–2849 (2016).

103. P. Langfelder, S. Horvath, WGCNA: An R package for weighted correlation network analysis. *BMC Bioinformatics* **9**, 559 (2008).

104. C. A. de Leeuw, J. M. Mooij, T. Heskes, D. Posthuma, MAGMA: Generalized gene-set analysis of GWAS data. *PLOS Comput. Biol.* **11**, e1004219 (2015).

105. S. Durinck, P. T. Spellman, E. Birney, W. Huber, Mapping identifiers for the integration of genomic datasets with the R/Bioconductor package biomaRt. *Nat. Protoc.* **4**, 1184–1191 (2009).

106. R Core Team, R: A language and environment for statistical computing (R Foundation for Statistical Computing, Vienna, Austria, 2018); available online at https://www.R-project.org/.
